# Facile Access to Substituted 1,4‐Diaza‐2,3‐Diborinines

**DOI:** 10.1002/chem.201905356

**Published:** 2020-02-21

**Authors:** Torsten Thiess, Moritz Ernst, Thomas Kupfer, Holger Braunschweig

**Affiliations:** ^1^ Institute of Inorganic Chemistry Julius-Maximilians-Universität Würzburg Am Hubland 97074 Würzburg Germany; ^2^ Institute for Sustainable Chemistry & Catalysis with Boron Julius-Maximilians-Universität Würzburg Am Hubland 97074 Würzburg Germany

**Keywords:** azadiboretidines, B,N-heterocycles, diazadiborinines, diboranes, ring expansion

## Abstract

Several bis(dimethylamino)‐substituted 1,4‐diaza‐2,3‐diborinines (DADBs) were synthesized with variable substituents at the backbone nitrogen atoms. By reaction with HCl or BX_3_ (X=Br, I), these species were successfully converted into their synthetically more useful halide congeners. The high versatility of the generated B−X bonds in further functionalization reactions at the boron centers was demonstrated by means of salt elimination (MeLi) and commutation (NMe_2_ DADBs) reactions, thus making the DADB system a general structural motif in diborane(4) chemistry. A total of 18 DADB derivatives were characterized in the solid state by X‐ray diffraction, revealing a strong dependence of the heterocyclic bonding parameters from the exocyclic substitution pattern at boron. According to our experiments towards the realization of a Dipp‐substituted, sterically encumbered DADB, the mechanism of DADB formation proceeds via a transient four‐membered azadiboretidine intermediate that subsequently undergoes ring expansion to afford the six‐membered DADB heterocycle.

## Graphical abstract

**Now established**: Convenient access to 1,4‐diaza‐2,3‐diborinines (DADBs) with variable steric demand of the backbone has been developed. Conversion of the initial bis(dimethylamino)‐substituted DADBs into synthetically more useful halide derivatives offers almost indefinite possibilities for further functionalization of the boron centers aiming at the synthesis of cyclic bis(amino)diboranes(4) with specifically tuned properties.

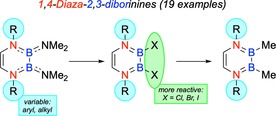

## Introduction

A recent review article by Marder and Westcott entitled diboranes(4) as synthetic workhorses.^1^ This is in fact true when considering the tremendous progress in the development of catalytic diboration and C‐borylation protocols including enantio‐selective variants.^1^, ^2^ Nowadays, diboranes(4) have become an indispensable tool in organic synthesis to prepare borylated substrates that serve as highly valuable building blocks for the construction of more complex molecules by subsequent Suzuki–Miyaura‐type cross‐coupling reactions.^3^ By far the most commonly applied diboron reagents are diboranes B_2_(OR)_4_ such as commercially available B_2_pin_2_ (pin=pinacolato) and B_2_cat_2_ (cat=catecholato), which provide the best compromise of availability, stability and reactivity.^1^, ^2^, ^3^ By contrast, the more reactive halide analogs B_2_X_4_ (X=Cl, Br, I) are difficult to handle and too labile for broad application,^1^, ^4^ whereas the more stable tetra(amino)diboranes B_2_(NR_2_)_4_, such as prototypical B_2_(NMe_2_)_4_, are often not sufficiently reactive to promote such processes. Actually, the effect of the amino groups on the reactivity of the B−B bond is dramatical, and only systems with a maximum of two amino groups were shown to participate in diboration/borylation reactions, that is, i) B_2_Cl_2_(NMe_2_)_2_,^5^ ii) strained [2]borametalloarenophanes,^6^ and (iii) unsymmetrical (OR)_2_B−B(NR_2_)_2_.^7^

**Figure 1:**
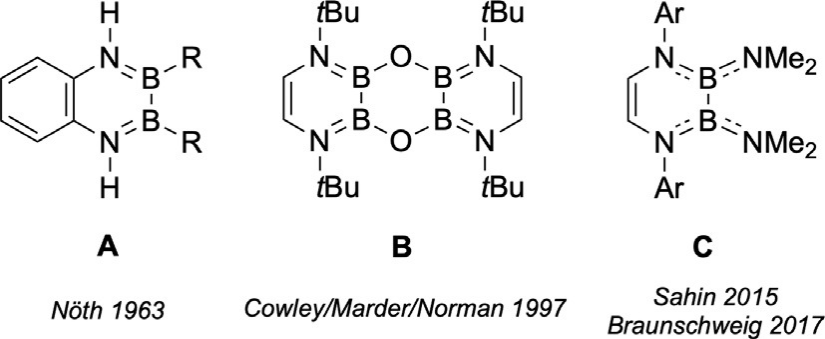
DADBs known in the literature (R=alkyl, Ar=aryl).

Given the fundamental importance of this research area, however, it is rather surprising that the structural diversity of stable (amino)diboranes(4) is still rather low, and dominated by the B(NMe_2_) fragment.^8^ This shortcoming is primarily a consequence of the non‐trivial B−B bond formation process, which is conveniently accomplished on a large scale only for B_2_(NMe_2_)_4_, making it the reagent of choice in diborane(4) chemistry. Thus, the synthesis of B_2_(NMe_2_)_4_ by Brotherton in 1960^9^ is generally recognized as the commencement of diborane(4) chemistry, and it has been established as a versatile and easy to handle reagent that is readily transformed into other diborane(4) species.^4^, ^10^ One strategy to significantly modify the structural appearance and properties of (amino)diboranes is the incorporation of the B‐N units into heterocyclic ring systems to generate for example 1,4‐diaza‐2,3‐diborinines (DADB, Figure 1).^11^ These cyclic (amino)diboranes(4) can be considered as B,N isosteres of benzene, in which two of the C=C bonds have been replaced by isoelectronic and isostructural B−N moieties. In sharp contrast to the well‐established chemistry of azaborinines,^12^ not much is known about the chemistry of DADBs so far, and only few examples have been realized. The first DADBs were isolated in 1963 as benzannulated derivatives by exchange amination of B_2_R_2_(NMe_2_)_2_ with aromatic amines (**A**, R=alkyl, Figure 1),^13^ while it was not until 1997 that a monocyclic oxygen‐bridged DADB dimer was reported as a byproduct (**B**, Figure 1).^14^ Very recently, a general and more selective route to monomeric DADBs (**C**, Figure 1) through salt elimination reactions of Li_2_[dab] salts (dab=1,4‐diazabutadiene) with dihalodiboranes(4) has been established independently by Sahin^15^ and by our group.^16^ So far, reactivity studies on type **C** DADBs are scarce and limited to the transformation of the NMe_2_‐substituted derivative **1 a** to its dihydrido analog and subsequent ring contraction/expansion reactions.^16^, ^17^ In this contribution, we will demonstrate that this route is easily expanded to include other backbones, and more importantly that the NMe_2_ group is very well suited for functionalization reactions without affecting the B_2_N_2_C_2_ heterocyclic core structure. Thus, a wide range of DADBs becomes accessible with high potential for further applications.

## Results and Discussion

**Scheme 1:**
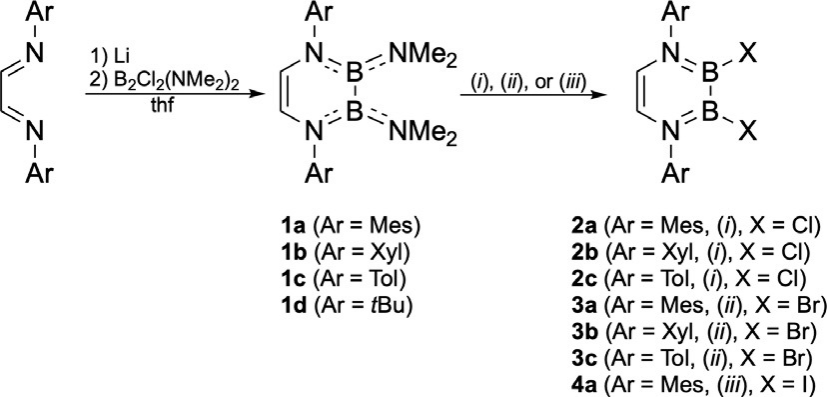
Synthesis of DADBs **1 a**–**d**, and conversion into halide derivatives **2 a**–**c**, **3 a**–**c**, and **4 a**. Reaction conditions: (i) 4 equiv. HCl, Et_2_O, −78 °C; (ii) 2 equiv. BBr_3_, pentane, −78 °C; (iii) 1 equiv. BI_3_, pentane, −78 °C.

To this end, we initially developed a more convenient protocol for the synthesis of mesityl‐substituted DADB **1 a** (Ar=Mes=2,4,6‐Me_3_‐C_6_H_2_), which does not require the isolation and purification of Li_2_[^Mes^dab] (Scheme 1).^16^ Through this, DADB **1 a** was readily obtained in moderate yields of 42 % even on a larger scale. To prove the generality of this approach, we also prepared related colorless DADBs **1 b**–**d** with xylyl (**1 b**; 32 %; Ar=Xyl=2,6‐Me_2_‐C_6_H_3_), *p*‐tolyl (**1 c**; 54 %; Ar=Tol=4‐Me‐C_6_H_4_), and *tert*‐butyl (**1 d**; 26 %; Ar=*t*Bu) groups attached to the backbone nitrogen atoms (Scheme 1).

**Figure 2:**
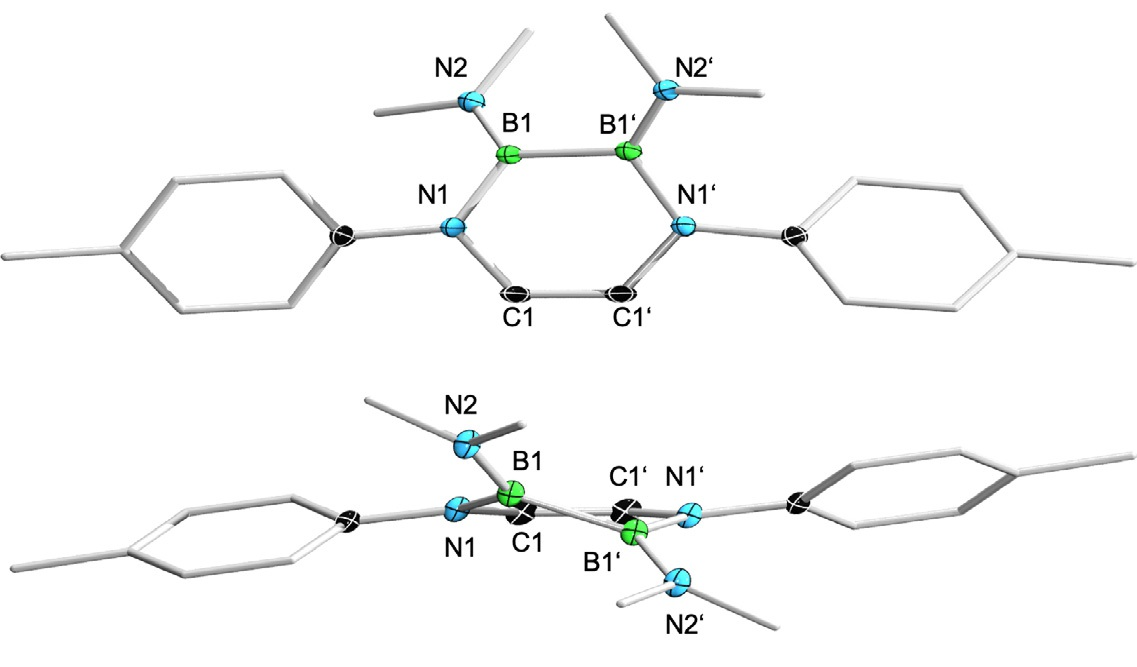
Two alternative views of the molecular structure of **1 c** in the solid state. The ellipsoids of the hydrogen and of some carbon atoms are omitted for clarity.

Compounds **1 b**–**d** are readily identified by their ^11^B NMR chemical shifts of *δ*=33.5 (**1 b**: ^11^B{^1^H}; *ω*_1/2_=1027 Hz), 34.1 (**1 c**: ^11^B{^1^H}; *ω*_1/2_=941 Hz), and 36.7 ppm (**1 d**: *ω*_1/2_=347 Hz) in [D_6_]benzene solutions, which strongly resemble those found for **1 a** (*δ*=33.5 ppm)^16^ and the 2,4‐xylyl derivative of Sahin (*δ*=32 ppm).^15^ In the solid state however, these species feature marked differences with respect to the planarity of the B_2_N_2_C_2_ heterocyclic core, which are caused by the nature and the sterics of the groups attached to the backbone nitrogen atoms. Thus, the molecular structure of **1 b** (Figure S62, Supporting Information) is virtually identical to that of **1 a**,^16^ both showing almost planar B_2_N_2_C_2_ rings (N1‐C1‐C2‐N2: **1 a** 2.7°; **1 b** 2.2(3)°. N1‐B1‐B2‐N2: **1 a** 6.2°; **1 b** 5.0(3)°). By contrast, the B_2_N_2_C_2_ rings of **1 c** (Figure 2) and **1 d** (Figure S64) deviate significantly from planarity (N1‐C1‐C2‐N2: **1 c** 6.7(2)°; **1 d** 8.8(2)°. N1‐B1‐B2‐N2: **1 c** 45.0(2)°; **1 b** 52.0(1)°), similar to the data reported by Sahin (N1‐C10‐C9‐N2: −5.5°. N1‐B1‐B2‐N2: −41.7°).^15^ For **1 a** and **1 b**, steric repulsion between the NMe_2_ and Mes/Xyl units entails twisting of the aryl groups to adopt an orthogonal arrangement with respect to the heterocyclic core structure, which generates sufficient space for the NMe_2_ groups in a planar B_2_N_2_C_2_ environment. In **1 d**, repulsive steric interactions between the NMe_2_ and the *t*Bu groups within a planar geometry would be inevitable, thus the B_2_N_2_C_2_ ring displays a flattened chair conformation. For **1 c** and Sahin's DADB, the planar and chair configurations seem to be close in energy, and the deviations from planarity observed in the solid state might already be caused by crystal packing effects favoring a coplanar arrangement of the aryl rings that necessarily provokes repulsion of the NMe_2_ units.

Short exocyclic B−N (**1 a**: 1.436(2), 1.433(2); **1 b**: 1.449(3), 1.430(3); **1 c**: 1.409(2); **1 d**: 1.406(2), 1.412(2) Å) and rather long B−B bond lengths (**1 a**: 1.719(2); **1 b**: 1.719(3); **1 c**: 1.708(2); **1 d**: 1.704(2) Å; calculated parent DADB B_2_N_2_C_2_H_6_ 1.6514 Å)^18^ are reminiscent of strong conjugative effects arising from the pendant NMe_2_ groups, observations that have been interpreted earlier for **1 a** in terms of aromaticity loss.^16^

**Figure 3:**
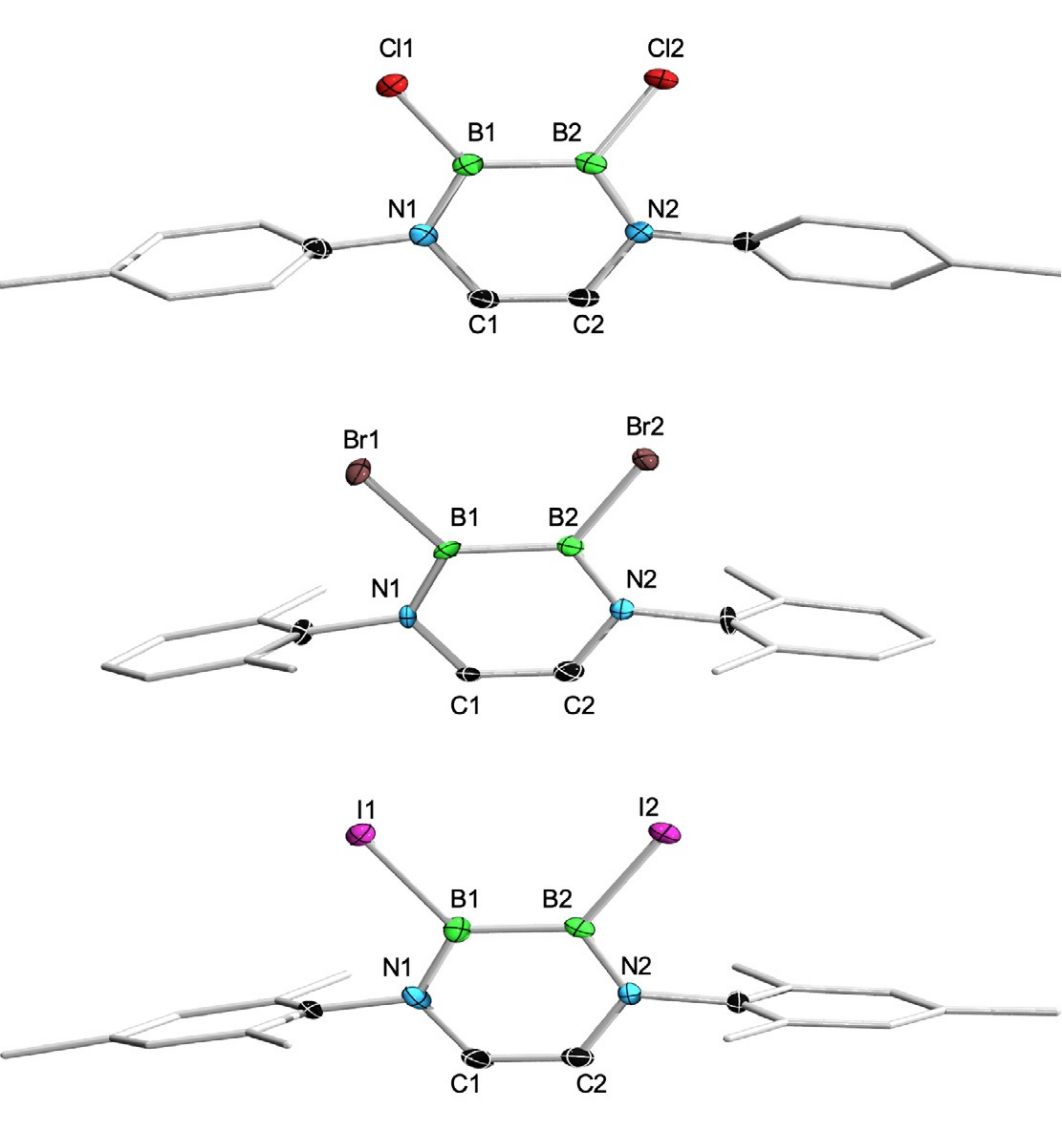
Molecular structures of **2 c** (top), **3 b** (middle), and **4 a** (bottom) in the solid state as representative examples for halide DADBs **2**–**4**. The ellipsoids of the hydrogen and of some carbon atoms are omitted for clarity.

We next wondered if the rather unreactive NMe_2_‐substituted DADBs can be converted into synthetically more useful species, and turned our attention to long‐established (amino)borane chemistry.^4^, ^10^ It is well documented that NMe_2_ groups attached to boron atoms are easily replaced by halides through reaction with HX or through commutation with BX_3_. Thus, we initially reacted **1 a** with either ethereal HCl, or with BBr_3_ and BI_3_ in pentane solutions (Scheme 1). Monitoring the reaction mixtures by NMR spectroscopy indicated smooth conversion into the halide DADB derivatives **2 a**, **3 a**, and **4 a** with concomitant formation of either [H_2_NMe_2_]Cl, or (amino)boranes (NMe_2_)_*n*_BX_3−*n*_. Compounds **2 a** (Cl, 59 %), **3 a** (Br, 81 %), and **4 a** (I, 28 %) were isolated as colorless materials in moderate to good yields depending on the nature of the halide substituent. Similarly, **1 b** and **1 c** afforded the related chloro (**2 b**, 36 %; **2 c**, 60 %) and bromo (**3 b**, 39; **3 c**, 21 %) DADBs under the same reaction conditions (Scheme 1). In solution, **2**–**4** are characterized by broad ^11^B NMR resonances in a narrow range between *δ*=40.5 and *δ*=42.0 ppm (**2 a**/**c**, **3 a**–**c**: ^11^B{^1^H}; *ω*_1/2_=462–1061 Hz. **2 b**, **4**: *ω*_1/2_=822, 1027 Hz). The observed shift of approximately 8 ppm to higher frequencies compared with the starting materials **1 a**–**c** meets the expectations associated with the replacement of the electron‐rich NMe_2_ groups by halide atoms. X‐ray diffraction studies served to validate the structural composition of **2**–**4** as suggested by NMR spectroscopy (Figure 3 and Figures S65–S71, Supporting Information). Accordingly, removal of the strongly electron‐donating NMe_2_ groups of **1 a**‐**c** entails marked structural changes of the B_2_N_2_C_2_ heterocyclic core structures. First of all, the smaller size of the halide substituents dramatically reduces the steric pressure within DADBs **2**–**4**, thus all species feature quasi planar B_2_N_2_C_2_ rings with torsion angles N1‐C1‐C2‐N2 between 0.3(3)° and 2.1(3)°, and torsion angles N1‐B1‐B2‐N2 between −1.8(8)° and 6.0(9)°. More importantly, the missing conjugation of the exocyclic NMe_2_ groups causes adjustment of the central B−B (1.653(3)–1.667(5) Å), B−N (1.375(6)–1.411(4) Å), and C−C (1.337(2)–1.352(6) Å) bond lengths of **2**–**4** to those calculated for parent B_2_N_2_C_2_H_6_ (B−B 1.6514 Å; B−N 1.4034 Å; C−C 1.3677 Å),^18^ indicating an enhancement in aromaticity of the heterocycles going from **1** to **2**–**4**. Furthermore, the aryl units are arranged orthogonal to the B_2_N_2_C_2_ rings in all halide‐substituted DADBs **2**–**4** prepared in this study, which is supportive for our argumentation that the coplanar arrangement found for **1 c** is more likely due to crystal packing than due to sterics.

**Scheme 2:**
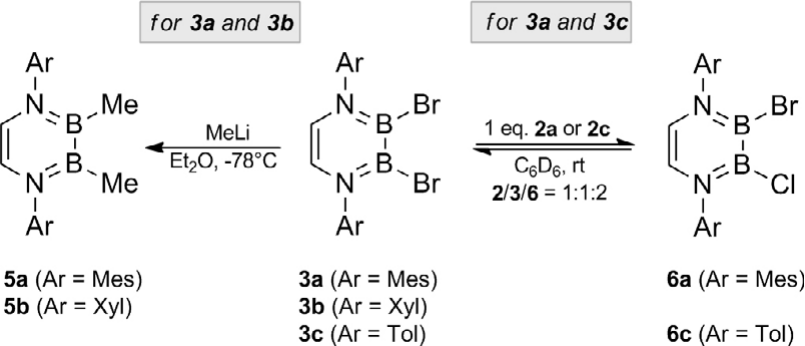
Reactivity of bromo‐substituted DADBs **3**. Left: Salt elimination reactions of **3 a** and **3 b** with MeLi to afford methylated **5 a** and **5 b**. Right: Commutation reactions with their chloro analogs to yield mixed species **6 a** and **6 c** as statistical equilibrium mixtures.

**Figure 4:**
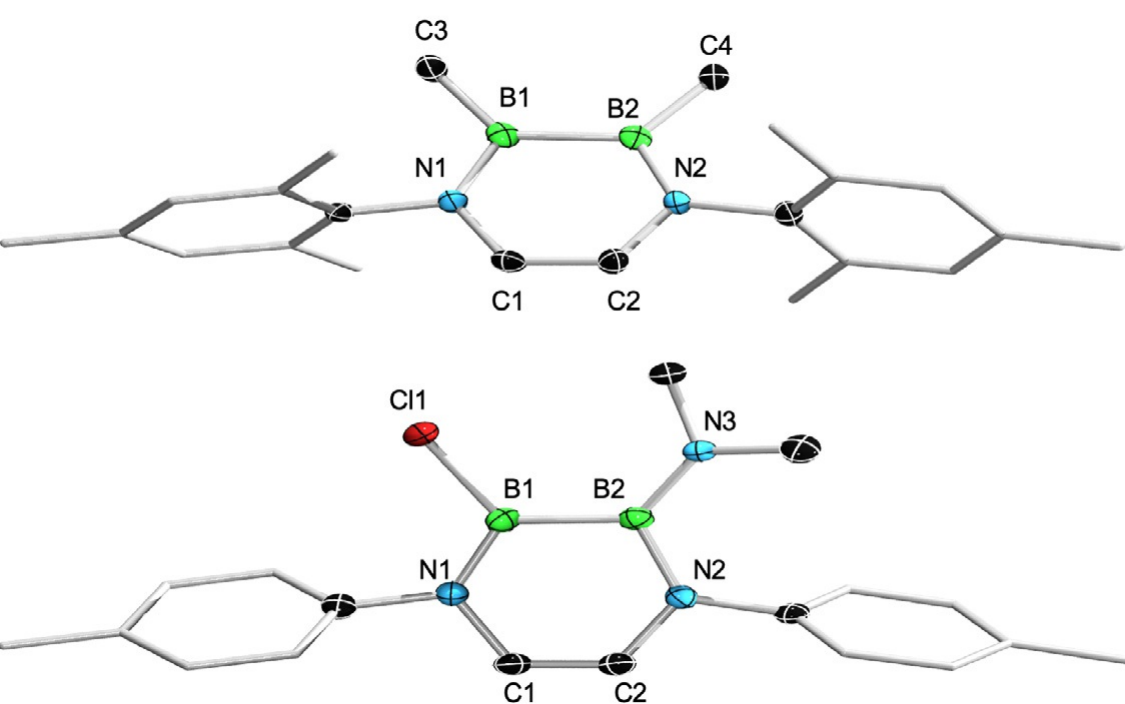
Molecular structures of **5 a** (top) and **7 c** (bottom) in the solid state as representative examples for methylated (**5**) and mixed NMe_2_/X‐substituted (**7**/**8**) DADBs. The ellipsoids of the hydrogen and of some carbon atoms are omitted for clarity.

**Figure 5:**
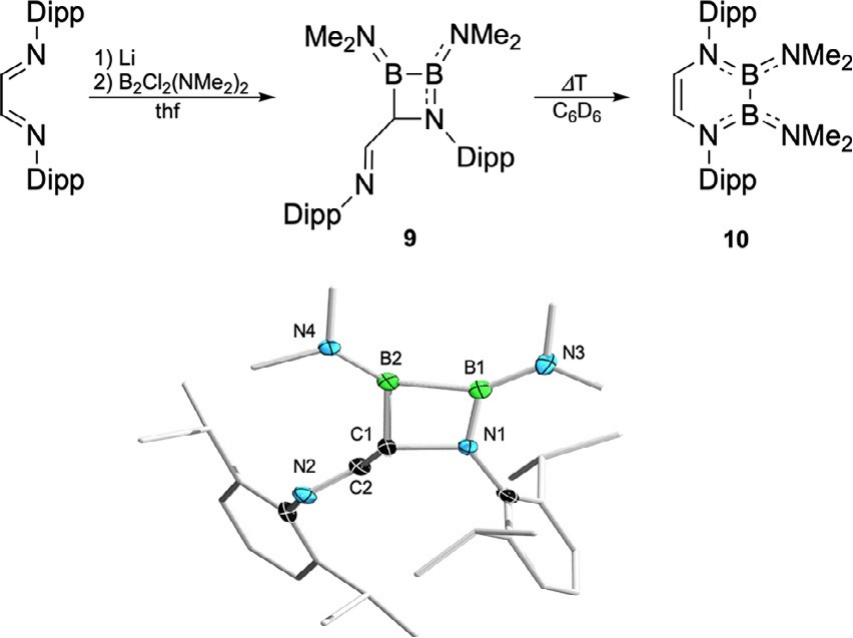
Experimental insights into the mechanism of DADB formation through salt elimination: Formation of azadiboretidine **9** and thermal ring expansion to DADB **10** (top). Molecular structure of **9** in the solid state (bottom). The ellipsoids of the hydrogen and of some carbon atoms are omitted for clarity.

We subsequently tried to assess the synthetic utility of the B−X bonds of DADBs **2**–**4** by studying their reactivity in salt elimination and commutation reactions. When reacted with two equivalents of MeLi in diethyl ether at low temperatures, the bromo derivatives **3 a** and **3 b** are smoothly converted into their methylated analogs **5 a** and **5 b**, respectively (Scheme 2), which are isolated as colorless solids in moderate yields of 52 % (**5 a**) and 41 % (**5 b**) after standard workup. Both species are characterized by broad ^11^B NMR resonances in solution (**5 a**: *δ*=49.0 ppm; *ω*_1/2_=1220 Hz. **5 b**: *δ*=48.5 ppm; *ω*_1/2_=1112 Hz) with chemical shifts at higher frequencies than their NMe_2_‐ (**1 a**,^16^ **1 b**: *δ*=33.5 ppm) and Br‐substituted (**3 a**: *δ*=42.0; **3 b**: *δ*=41.4 ppm) counterparts. The solid‐state structures (X‐ray diffraction) of **5 a** (Figure 4) and **5 b** (Figure S73, Supporting Information) reveal similar metrical parameters and strongly resemble those of their precursor molecules, thus accounting for the similar steric demand of the bromine (**3**) and methyl (**5**) substituents. Hence, **5 a** and **5 b** exhibit conjugated (B−B: **5 a** 1.688(5); **5 b**: 1.684(3). B−N: **5 a** 1.392(4), 1.426(5); **5 b**: 1.400(2), 1.422(2). C−C: **5 a** 1.335(5); **5 b**: 1.331(3) Å) and planar (N1‐C1‐C2‐N2: **5 a** −1.5(5)°; **5 b** 0.7(3)°. N1‐B1‐B2‐N2: **5 a** −4.7(4)°; **5 b** −2.5(2)°) B_2_N_2_C_2_ heterocycles, also reminiscent of the parent diazadiborinine B_2_N_2_C_2_H_6_ (see above).^18^ The B−C bonds to the methyl groups are well within the expected range for such single bonds, and the average B−Me distance of DADBs **5** is measured to be 1.577 Å.^19^

**Scheme 3:**
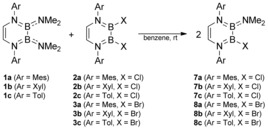
Commutation reactions between NMe_2_‐ (**1**) and halide‐substituted (**2**/**3**) DADBs to afford the corresponding mixed species **7** and **8**.

As outlined in Schemes 2 (right) and 3, the B−X bonds of DADBs **2** and **3** are also amenable to functionalization through classical commutation reactions. For instance, halide exchange is observed upon treatment of the bromo DADBs **3 a** and **3 c** with their chloro analogues **2 a** and **2 c**, respectively (Scheme 2, right). Here, ^1^H NMR spectroscopy clearly showed that statistical equilibrium mixtures are established containing the expected 1:1:2 ratio of chloro (**2 a**/**c**), bromo (**3 a**/**c**), and mixed chloro/bromo (**6 a**/**c**) DADBs. According to variable‐temperature (VT) NMR studies, these equilibria are temperature‐independent, and we were not able to separate **6 a** and **6 c** from the reaction mixtures. Similarly, equimolar reactions of halide derivatives **2 a**–**c** and **3 a**–**c** with their respective NMe_2_ counterparts **1 a**–**c** selectively afforded the mixed NMe_2_/X DADBs **7 a**–**c** (X=Cl) and **8 a**–**c** (X=Br) through amide–halide exchange processes (Scheme 3). In all cases, the conversion proceeded quantitatively at room temperature, and colorless powders were isolated in yields up to 94 %. The solution NMR spectra of **7 a**–**c** and **8 a–c** are very similar, and all compounds feature two distinct ^11^B NMR resonances with chemical shifts characteristic for the B−NMe_2_ (*δ*=30.4–32.0 ppm; *ω*_1/2_=821–1065 Hz) and B−X (*δ*=40.4–41.1 ppm; *ω*_1/2_=873–1207 Hz) boron centers (see DADBs **1**–**4**).

We were also able to verify the unsymmetrical substitution pattern of the diborane moiety for all mixed NMe_2_/X DADBs in the solid state by X‐ray diffraction (Figures 4 and S74–S79, Supporting Information), and the molecular structure of **7 c** is shown as representative example in Figure 4. Again, the key structural parameters of the quasi‐planar B_2_N_2_C_2_ heterocycles (N1‐C1‐C2‐N2: −0.4(6)–2.6(2)°; N1‐B1‐B2‐N2: 1.3(6)–6.6(3)°) are mainly determined by the electronics of the exocyclic substituents at boron. The presence of a strongly electron‐donating NMe_2_ group entails B−N conjugative interactions, which lower the aromaticity of the B_2_N_2_C_2_ ring compared to DADBs **3**–**5** and parent B_2_N_2_C_2_H_6_. Even though this effect is not as dramatic as for the bis(amino)‐substituted precursors **1 a**–**c**, it is well measurable particularly for the B−B bond lengths (1.692(4)–1.699(2) Å), which show values in‐between those of the NMe_2_ (**1 a**–**c**: 1.708(2)–1.719(3) Å) and halide (**2/3 a**–**c**: 1.653(3)–1.667(5) Å) precursors (B_2_N_2_C_2_H_6_: 1.6514 Å).^18^ The unsymmetrical nature of **7 a**–**c** and **8 a**–**c** is also visible in the B1−N1 (1.396(7)–1.408(5) Å) and B2−N2 (1.453(6)–1.457(2) Å) bonds, which are significantly longer for B2 due to additional conjugation with the pendant NMe_2_ group. Similar findings have already been described for a mixed NMe_2_/H DADB (B−N: 1.398(6) and 1.449(6) Å).^16^

As part of our efforts to prepare the sterically demanding DADB **10** with bulky Dipp (Dipp=2,6‐*i*Pr_2_‐C_6_H_3_) groups at the backbone nitrogen atoms, we caught unexpected insights into the mechanism of DADB formation (Figure 5). Accordingly, reactions of Li_2_[dab] with B_2_Cl_2_(NMe_2_)_2_ most likely do not directly afford the six‐membered B_2_N_2_C_2_ heterocyclic DADBs, but rather proceed via transient four‐membered azadiboretidine intermediates that subsequently undergo thermal ring‐expansion reactions to yield DADBs. Obviously, the immense steric demand of the Dipp group provides sufficient kinetic stabilization for azadiboretidine **9** to efficiently hamper DADB formation at room temperature, and to allow for the isolation of **9** as colorless crystals (26 %) (Figure 5, top). Compound **9** is not indefinitely stable at ambient conditions and slowly converts to DADB **10** both in solution and in the solid state. Nevertheless, NMR spectroscopic and X‐ray diffraction studies (Figure 5, bottom) clearly verified the classification of **9** as 1,2,3‐azadi‐boretidine, a very rare class of boron‐containing heterocycles. Although a small number of isomeric 1,2,4‐azadiboretidines are known in the literature,^20^ only one 1,2,3‐azadiboretidine has been mentioned so far, the identity of which, however, remains questionable.^21^ In solution, **9** exhibits two distinct ^11^B NMR signals at *δ*=33.1 (B1; *ω*_1/2_=864 Hz) and *δ*=48.6 ppm (B2; *ω*_1/2_=1049 Hz) with chemical shifts in the expected region for such substitution patterns.

**Figure 6:**
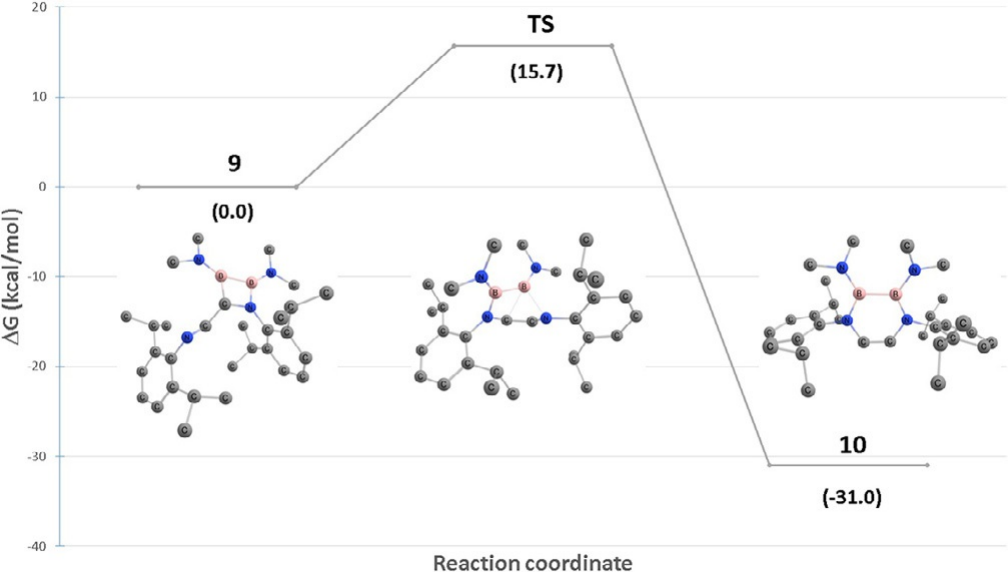
Calculated mechanism for the conversion of **9** to **10** via transition state **TS**. Energies are given as Δ*G* in kcal mol^−1^ at the *ω*B97xd/6‐31G* level of theory.

In the solid state, the central four‐membered B_2_NC ring of **9** is almost perfectly planar with a torsion angle C1‐B2‐B1‐N1 of 1.8(1)°, which contrasts the butterfly type structures of 1,2,4‐azadiboretidines.^20^ The bonds B1−B2 (1.720(3) Å), B2−C1 (1.632(3) Å), and C1−N1 (1.494(2) Å) show values typically encountered for such single bonds. The B1−N1 bond length of 1.441(3) Å suggests some degree of conjugation, which effects a noticeable elongation of the exocyclic B1−N3 bond (1.401(3) Å) compared to the B2−N4 bond (1.370(3) Å). Unfortunately, bonding parameters of **9** cannot be discussed in relation to other 1,2,3‐azadiboretidines, for which structural information are absent. In line with azadiboretidine **9** being an intermediate on the way to DADB formation, **9** was converted quantitatively to DADB **10** by heating benzene solutions at 60 °C for two hours (Figure 5, top). The ring expansion reaction proceeded with high selectivity, thus analytically pure **10** was isolated by simple evaporation of the solvent. We have not been able to obtain single crystals of **10** for structural characterization in the solid state, however, NMR spectroscopy in solution (^11^B: *δ*=33.8 ppm; *ω*_1/2_=1090 Hz. See **1 a**‐**d**: *δ*=33.5–36.7 ppm) and elemental analysis convincingly substantiate its 1,4‐diaza‐2,3‐diborinine identity. According to quantum‐chemical DFT calculations, the conversion of **9** to **10** is highly exothermic (Δ*G*=31.0 kcal mol^−1^) and proceeds via concerted transition state **TS**, in which the B2−C1 and B2−N2 bonds are simultaneously broken and formed, respectively (Figure 6). With a calculated Δ*G* of 15.7 kcal mol^−1^, **TS** is thermally accessible, which is consistent with the observed lability of **9** even in the solid state.

## Conclusions

With our present study, we have significantly expanded the scope of known 1,4‐diaza‐2,3‐diborinines (DADBs), a class of cyclic (amino)diborane(4) molecules neglected for a long time. We have shown that the synthetic approach to enter DADB chemistry, the synthesis of doubly NMe_2_‐substituted DADBs **1** via one‐pot salt metathesis reactions of Li_2_[dab] with B_2_Cl_2_(NMe_2_)_2_, also provides the possibility to simple vary the steric demand of the DADB backbone. Most importantly, we succeeded in converting these rather unreactive molecules into their synthetically more useful dihalide derivatives **2**–**4** by convenient methods long‐established in diborane(4) chemistry. Subsequent salt elimination reactions with MeLi (**5**) and commutation reactions with NMe_2_ DADBs **1** (**7**/**8**) served to demonstrate the high versatility of the B−X bonds for further functionalization at the boron centers. Thus, the DADB system becomes highly relevant as a general structural motif in diborane(4) chemistry allowing for the targeted synthesis of (amino)diboranes(4) with highly specific properties for applications in borylation/diboration processes. Inspection of the solid‐state structures of a total of 18 DADBs established a strong dependence of the heterocyclic B_2_N_2_C_2_ bonding parameters and thus its aromaticity from the electronics of the boron‐bound substituents. With the isolation of the first structurally authenticated 1,2,3‐azadiboretidine **9**, we were also able to shed some light onto the mechanism of DADB formation. Currently, we are forcefully exploring the scope of substituents that can be attached to the DADB boron centers via halide ADBs **2**–**4** aiming at creation of a large DADB database. Concomitantly, we are testing the new DADBs with respect to their suitability in catalytic processes involving diboranes(4).

## Experimental Section

Synthesis and characterization of new compounds, NMR spectra, crystallographic details, and supplementary structures can be found in the Supporting Information. CCDC https://www.ccdc.cam.ac.uk/services/structures?id=doi:10.1002/chem.201905356 contain the supplementary crystallographic data for this paper. These data are provided free of charge by http://www.ccdc.cam.ac.uk/.

## Conflict of interest

The authors declare no conflict of interest.

## Supporting information

As a service to our authors and readers, this journal provides supporting information supplied by the authors. Such materials are peer reviewed and may be re‐organized for online delivery, but are not copy‐edited or typeset. Technical support issues arising from supporting information (other than missing files) should be addressed to the authors.

Supplementary
