# Supplementary material for: Facile Access to Substituted 1,4‐Diaza‐2,3‐Diborinines
Source: Chemistry. 2020 Feb 21;26(13):2967–72. doi: 10.1002/chem.201905356 (PMC7078994; doi:10.1002/chem.201905356)
Supplement: Supplementary file 1 — Supplementary [file CHEM-26-2967-s001.pdf]

CHEMISTRY
A European Journal

Supporting Information

Facile Access to Substituted 1,4-Diaza-2,3-Diborinines

Torsten Thiess,[a, b] Moritz Ernst,[a, b] Thomas Kupfer,[a, b] and Holger Braunschweig*[a, b]

chem_201905356_sm_miscellaneous_information.pdf

Supporting Information for

Facile access to substituted 1,4-diaza-2,3-diborinines

Torsten Thiess,[a,b] Moritz Ernst,[a,b] Thomas Kupfer,[a,b] and Holger Braunschweig*[a,b]

[a] Institute of Inorganic Chemistry, Julius-Maximilians-Universität Würzburg, Am Hubland, 97074 Würzburg, Germany

[b] Institute for Sustainable Chemistry & Catalysis with Boron, Julius-Maximilians-Universität Würzburg, Am Hubland, 97074 Würzburg, Germany

e-mail: h.braunschweig@uni-wuerzburg.de

This PDF file includes:

Materials and Methods
Figures S1 to S80
References

S1 Synthetic details and characterization of compounds

General experimental considerations: All manipulations were performed either under an atmosphere of dry argon, or in vacuo using standard Schlenk line or glovebox techniques. Deuterated solvents were dried over molecular sieves and degassed by three freeze-pump-thaw cycles prior to use. All other solvents were distilled and degassed from appropriate drying agents. Solvents (both deuterated and non-deuterated) were stored under argon over activated 4 Å molecular sieves. All glassware was oven-dried prior to use. Commercially available BCl_3, and $B_2(NMe_2)_4$ were used without further purification. BBr_3 was stirred over elemental mercury, distilled and stored under argon atmosphere. Ethereal HCl was prepared by passing gaseous HCl through Et_2O at 0 °C and subsequent titration using 0.1 N NaOH solutions. Lithium metal stored under mineral oil was washed with pentane, and added to the reaction quickly. *N,N'*-Dimesityl-1,4-diazabutadiene,[1] *N,N*-bis(2,6-dimethylphenyl)-1,4-diazabutadiene),[2] *N,N'*-bis(*p*-tolyl)-1,4-diazabutadiene,[3] *N,N'*-bis(*tert*-butyl)-1,4-diazabutadiene,[4] *N,N'*-bis(2,6-di*iso*propylphenyl)-1,4-diazabutadiene,[1] and 1,2-dichloro-1,2-bis(dimethylamino)diborane(4)[5] were synthesized as described in the literature. NMR spectra were acquired on a Bruker Avance 500 NMR spectrometer (^{1}H: 500.1 MHz, ^{11}B: 160.5 MHz, ^{13}C: 125.8 MHz). Chemical shifts (δ) are given in ppm and internally referenced to the carbon nuclei (^{13}C{^{1}H}) or residual protons (^{1}H) of the solvent. NMR spectra were referenced to $SiMe_4$ (^{1}H, ^{13}C) and $BF_3 \cdot OEt_2$ (^{11}B, ^{11}B{^{1}H}) as external standards. ^{1}H and ^{13}C{^{1}H} NMR signals were assigned with assistance of DEPT-135, HMBC, and HSQC NMR experiments. Full widths at half maximum $\omega_{1/2}$ are given in Hz. Resonances are given as singlet (s), doublet (d), septet (sept) or multiplet (m). Microanalyses (C, H, N) were performed on an Elementar vario MICRO cube elemental analyser.

Synthesis and characterization of 1a

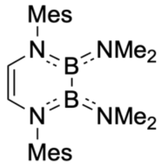

1a was synthesized by a slight modification of the literature procedure.[6] The dilithium salt $Li_2[^{Mes}dab]$ was generated *in situ* in THF, and reacted with $B_2Cl_2(NMe_2)_2$ at room temperature without isolation and without further purification. The crude product was extracted into pentane, and the solution was stored in the freezer at –30 °C to give **1a** in 42% yield. The spectroscopic parameters were in excellent agreement to those described in the literature.[6]

Synthesis and characterization of 1b

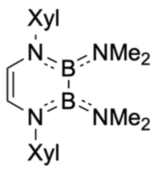

To a solution of *N,N'*-bis(2,6-dimethylphenyl)-1,4-diazabutadiene (1.09 g, 4.35 mmol) in THF (50 mL) was added excess lithium metal, and the solution was stirred at ambient temperature until an intense yellow color has formed. The mixture was cannulated to remove excess lithium, and treated dropwise with a solution of $B_2Cl_2(NMe_2)_2$ (0.69 mL, 775 mg, 4.12 mmol) in THF (15 mL). The reaction mixture was stirred at room temperature over a period of 16 h resulting in the formation of a dark red solution. All volatiles were removed *in vacuo*, and the crude product was extracted into pentane, concentrated and stored at -30 °C overnight to yield colorless block-shaped crystals. The mother liquor was removed, the crystals washed with cold pentane and dried *in vacuo* to yield 1,4-bis(2,6-dimethylphenyl)-2,3-bis(dimethylamino)-1,4-diaza-2,3-diborinine (**1b**) (500 mg, 1.34 mmol, 32%). Crystals suitable for X-ray diffraction were obtained from saturated pentane solutions at -30 °C.

^{1}H-NMR (500 MHz, C_6D_6): δ = 7.02-7.00 (m, 6H, aryl-C*H*), 5.17 (s, 2H, C=C*H*), 2.47 (s, 12H, N(C*H*$_3$)$_2$), 2.29 (s, 12H, C*H*$_3$). **^{11}B{^{1}H}-NMR** (160 MHz, C_6D_6): δ = 33.5 (s, br, $\omega_{1/2}$ = 1027). **^{13}C{^{1}H}-NMR** (125 MHz, C_6D_6): δ = 148.98 (s, *ipso*-*C*$_q$), 135.41 (s, *ortho*-*C*$_q$), 128.42 (s, *meta*-*C*), 126.04 (s, *para*-*C*H), 116.24 (s, C=*C*H), 40.99 (s, N(*C*H$_3$)$_2$), 18.83 (s, *ortho*-*C*H$_3$). **El. Anal.** [%]: calculated for $C_{22}H_{32}B_2N_4$ (374.15 g·mol^{-1}): C 70.63, H 8.62, N 14.97; found: C 70.66, H 8.57, N 14.53.

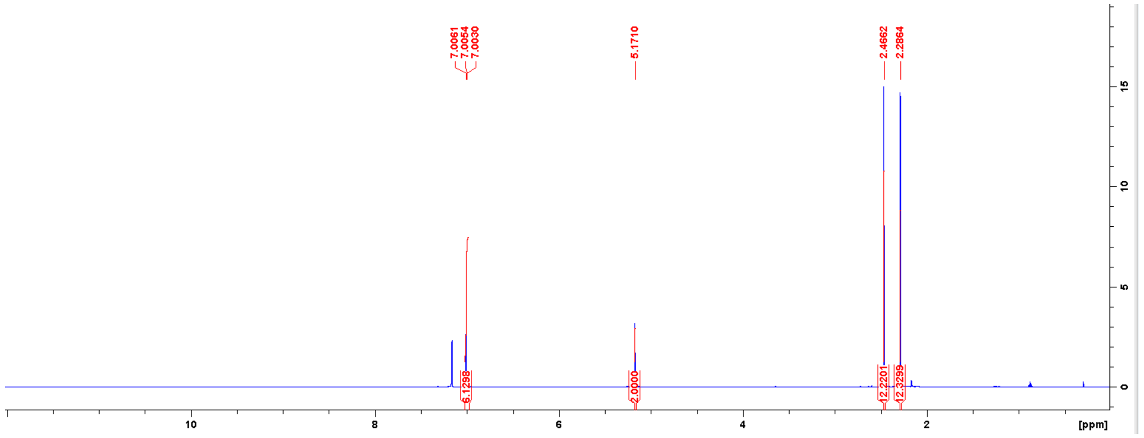

Figure S1. ^{1}H NMR spectrum of **1b** in C_6D_6.

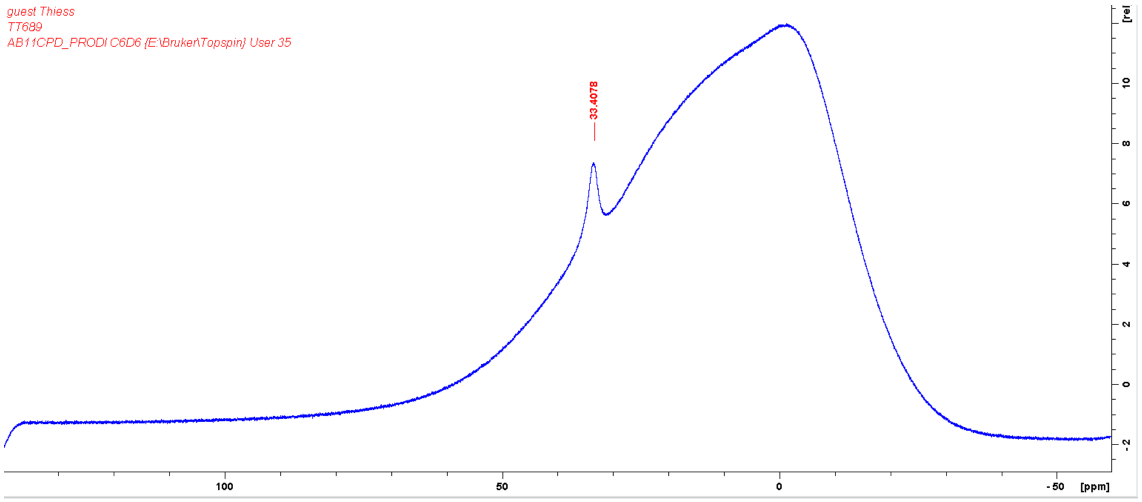

Figure S2. ^{11}B NMR spectrum of **1b** in C_6D_6.

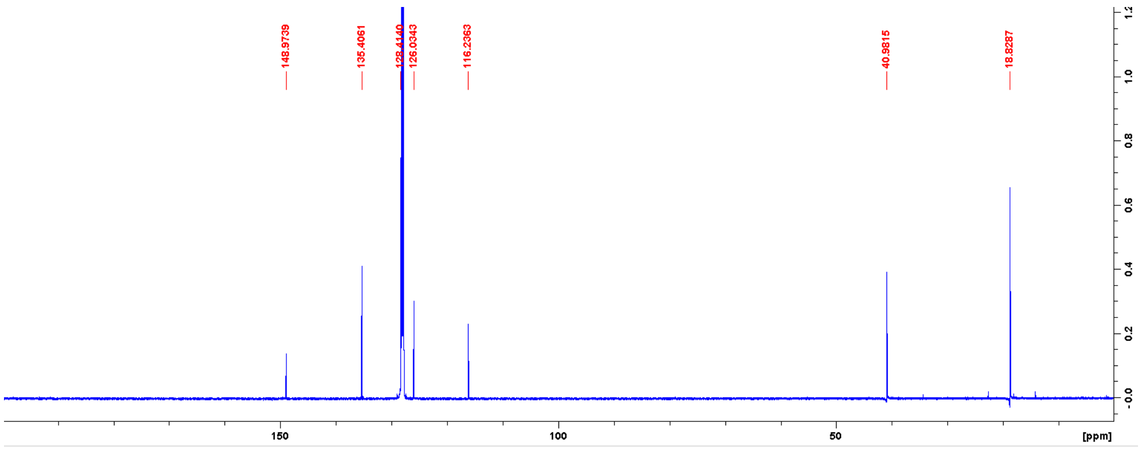

Figure S3. ^{13}C NMR spectrum of **1b** in C_6D_6.

Synthesis and characterization of 1c

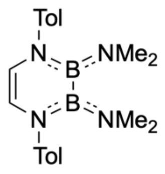

To a solution of *N,N'*-bis(*para*-tolyl)-1,4-diazabutadiene (2.05 g, 8.67 mmol) in THF (50 mL) was added excess lithium metal, and the solution was stirred at ambient temperature until an intense yellow color has formed. The mixture was cannulated to remove excess lithium, and treated dropwise with a solution of $B_2Cl_2(NMe_2)_2$ (1.45 mL, 1.57 g, 8.67 mmol) in THF (20 mL). The reaction mixture was stirred at room temperature over a period of 16 h resulting in the formation of a dark red solution. All volatiles were removed *in vacuo*, and the crude product was extracted into pentane, concentrated and stored at -30 °C overnight to give colorless crystals. The mother liquor was removed, the crystals washed with cold pentane and dried *in vacuo* to yield 1,4-bis(*para*-

tolyl)-2,3-bis(dimethylamino)-1,4-diaza-2,3-diborinine (**1c**) (1.62 g, 4.68 mmol, 54%). Crystals suitable for X-ray diffraction were obtained from saturated pentane solutions at -30 °C.

^{1}H-NMR (500 MHz, C_6D_6): δ = 7.05-6.99 (m, 8H, aryl-C*H*), 6.03 (s, 2H, HC=C*H*), 2.52 (s, 12H, N(C*H*$_3$)$_2$), 2.16 (s, 6H, *para*-C*H*$_3$). **^{11}B{^{1}H}-NMR** (160 MHz, C_6D_6): δ = 34.1 (s, br, $\omega_{1/2}$ = 941). **^{13}C{^{1}H}-NMR** (125 MHz, C_6D_6): δ = 147.36 (s, *ipso*-*C*$_q$), 130.87 (s, *ortho*-*C*H), 130.06 (s, *meta*-*C*H), 122.59 (s, *para*-*C*$_q$), 113.48 (s, HC=*C*H), 42.09 (s, N(*C*H$_3$)$_2$), 20.81 (s, *para*-*C*H$_3$). **El. Anal. [%]:** calculated for $C_{20}H_{28}B_2N_4$ (346.09 g·mol^{-1}): C 69.41, H 8.16, N 16.19; found: C 69.24, H 8.10, N 16.04.

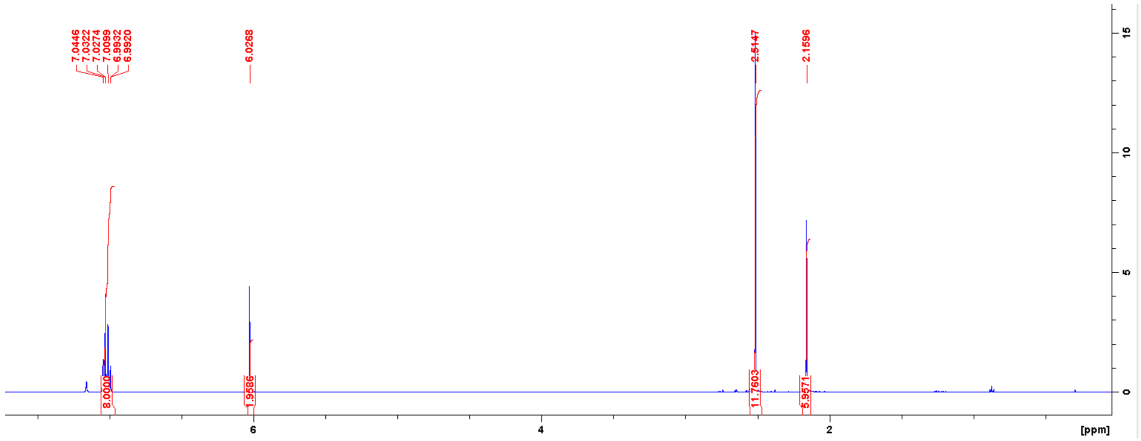

Figure S4. ^{1}H NMR spectrum of **1c** in C_6D_6.

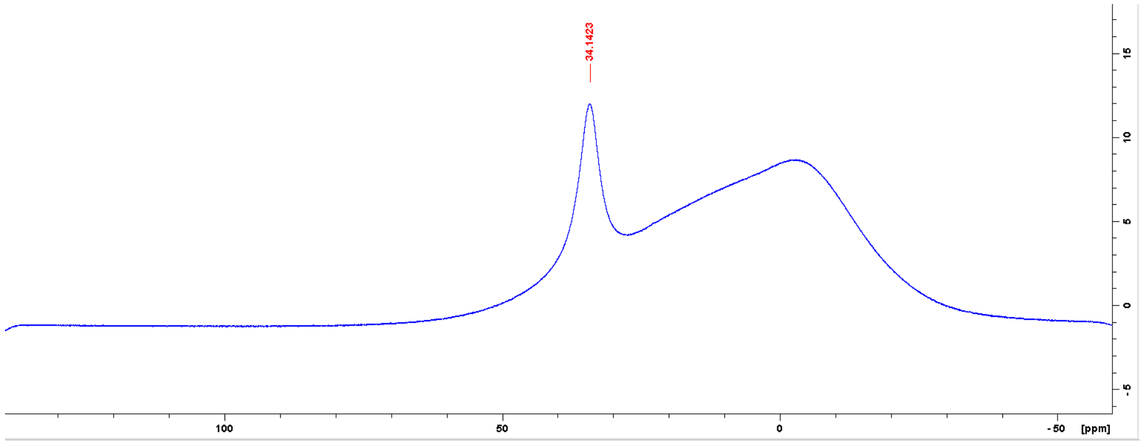

Figure S5. ^{11}B NMR spectrum of **1c** in C_6D_6.

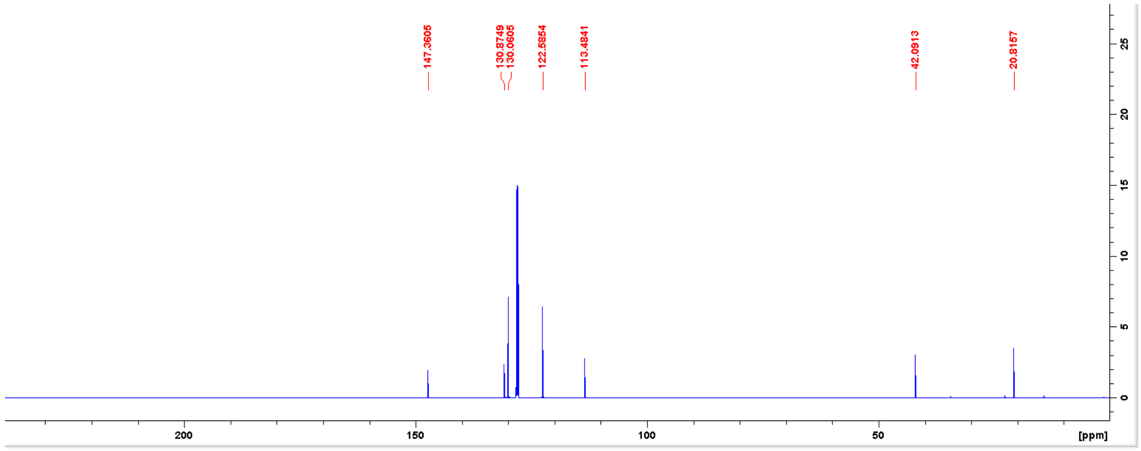

Figure S6. ^{13}C NMR spectrum of **1c** in C_6D_6.

Synthesis and characterization of 1d

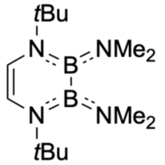

To a solution of *N,N'*-bis(*tert*-butyl)-1,4-diazabutadiene (1.05 g, 6.24 mmol) in THF (50 mL) was added excess lithium metal, and the solution was stirred at ambient temperature until an intense yellow color has formed. The mixture was cannulated to remove excess lithium, cooled to -78 °C. and treated dropwise with a solution of $B_2Cl_2(NMe_2)_2$ (1.04 mL, 1.13 g, 6.24 mmol) in THF (20 mL). The reaction mixture was allowed to reach room temperature, stirred over a period of 16 h, and heated at reflux for 10 minutes resulting in the formation of a dark red solution. All volatiles were removed *in vacuo*, and the crude product was extracted into pentane, concentrated and stored at -30 °C overnight to give colorless crystals. The mother liquor was removed, the crystals washed with cold pentane and dried *in vacuo* to yield 1,4-bis(*tert*-butyl)-2,3-bis(dimethylamino)-1,4-diaza-2,3-diborinine (**1d**) (452.3 mg, 1.63 mmol, 26%). Crystals suitable for X-ray diffraction were obtained from saturated pentane solutions at -30 °C.

^{1}H-NMR (500 MHz, C_6D_6): δ = 5.45 (s 2H, C*H*), 2,71 (s, 12H, N(CH_3)$_2$), 1.30 (s, 18H, C(CH_3)$_3$). **^{11}B-NMR** (160 MHz, C_6D_6): δ = 36.7 (s, br, $\omega_{1/2}$ = 347). **^{13}C{^{1}H}-NMR** (125 MHz, C_6D_6): δ = 110.1 (s, *C*H), 54.1 (*C*(CH$_3$)$_3$), 41.7 (s, C(*C*H$_3$)$_3$), 30.9 (s, N(*C*H$_3$)$_2$). **El. Anal.** [%]: calculated for $C_{14}H_{32}B_2N_4$ (278.06 g·mol^{-1}): C 60.47, H 11.60, N 20.15; found: C 60.32, H 11.53, N 20.43.

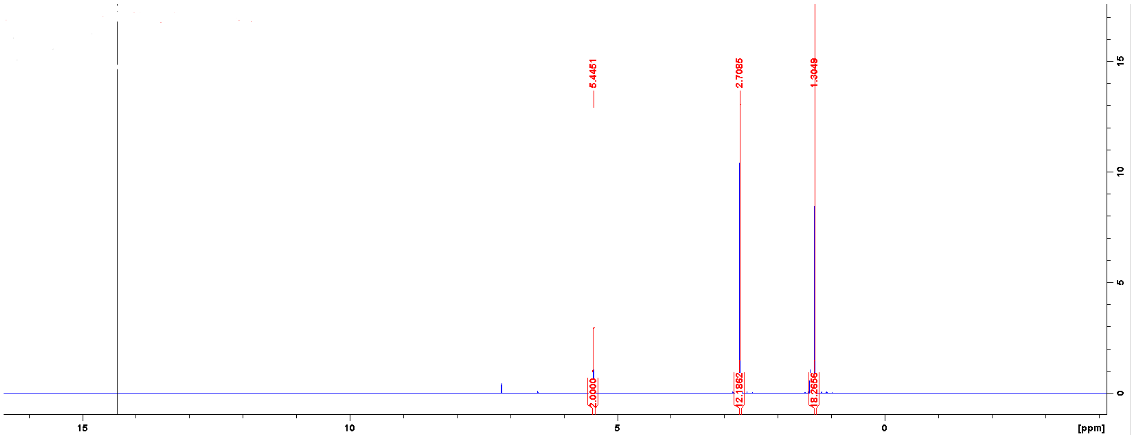

Figure S7. ^{1}H NMR spectrum of **1d** in C_6D_6.

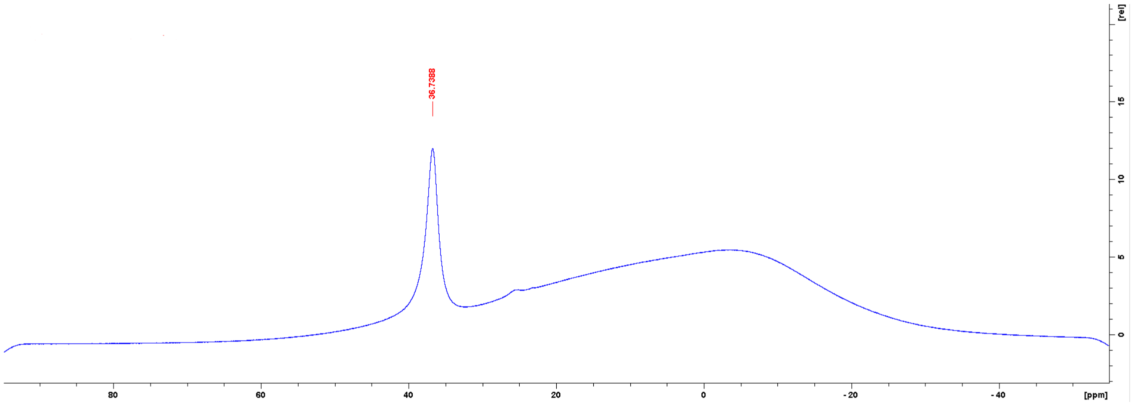

Figure S8. ^{11}B NMR spectrum of **1d** in C_6D_6.

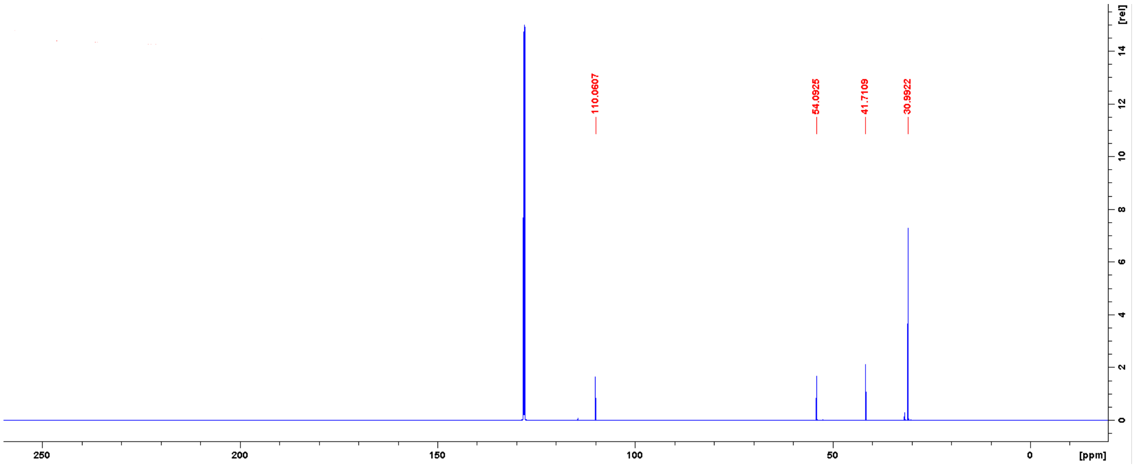

Figure S9. ^{13}C NMR spectrum of **1d** in C_6D_6.

Synthesis and characterization of 2a

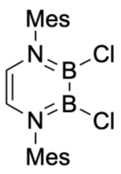

A solution of **1a** (1.00 g, 2.49 mmol) in Et_2O (20 mL) was cooled to -78 °C and treated dropwise with ethereal HCl (4.74 mL, 363 mg, 9.95 mmol, c = 2.1 mol/L, 4 eq.), whereupon the formation of a colorless precipitate was observed. The reaction mixture was allowed to warm to room temperature overnight, and all volatiles were removed *in vacuo*. The residue was extracted into Et_2O, concentrated, and stored at -30 °C overnight to afford needle-shaped colorless crystals. The mother liquor was removed, the crystals washed with cold Et_2O and dried *in vacuo* to yield 1,4-dimesityl-2,3-dichloro-1,4-diaza-2,3-diborinine (**2a**) (568 mg, 1.48 mmol, 59%). Crystals suitable for X-ray diffraction were obtained from saturated Et_2O solutions at -30 C.

^{1}H-NMR (500 MHz, C_6D_6): δ = 6.78 (s, 4H, aryl-C*H*), 5.59 (s, 2H, C*H*), 2.12 (s, 6H, *para*-CH_3), 2.04 (s, 12H, *orhto*-CH_3). **^{11}B{^{1}H}-NMR** (160 MHz, C_6D_6): δ = 40.9 (s, br, $\omega_{1/2}$ = 667). **^{13}C{^{1}H}-NMR** (125 MHz, C_6D_6): δ = 142.0 (s, *ipso*-C_q), 137.1 (s, *para*-C_q), 133.1 (s, *ortho*-C_q), 129.5 (s, *meta*-CH), 120.1 (s, CH), 21.0 (s, *para*-CH_3), 17.9 (s, *ortho*-CH_3). **El. Anal. [%]**: calculated for $C_{20}H_{24}B_2Cl_2N_2$ (384.95 g·mol^{-1}): C 62.40, H 6.28, N 7.28; found: C 62.20, H 6.49, N 7.77.

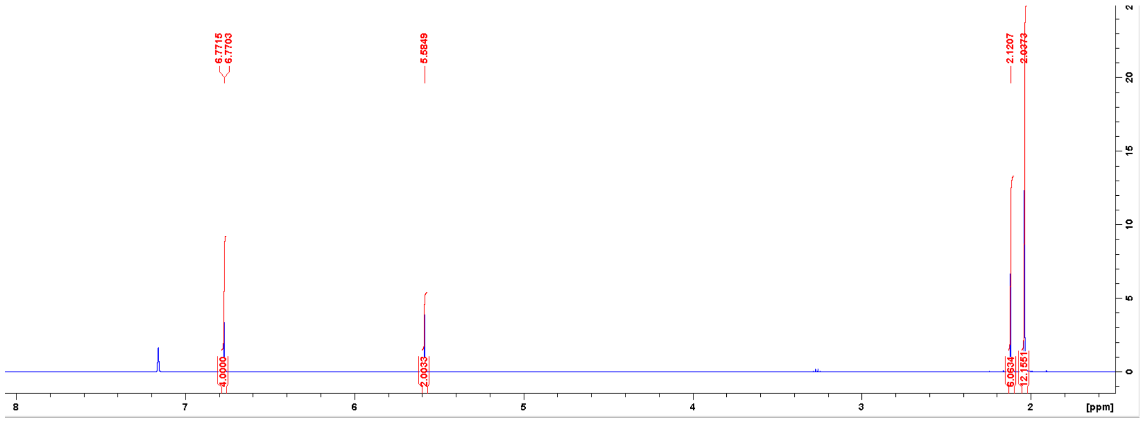

Figure S10. ^{1}H NMR spectrum of **2a** in C_6D_6.

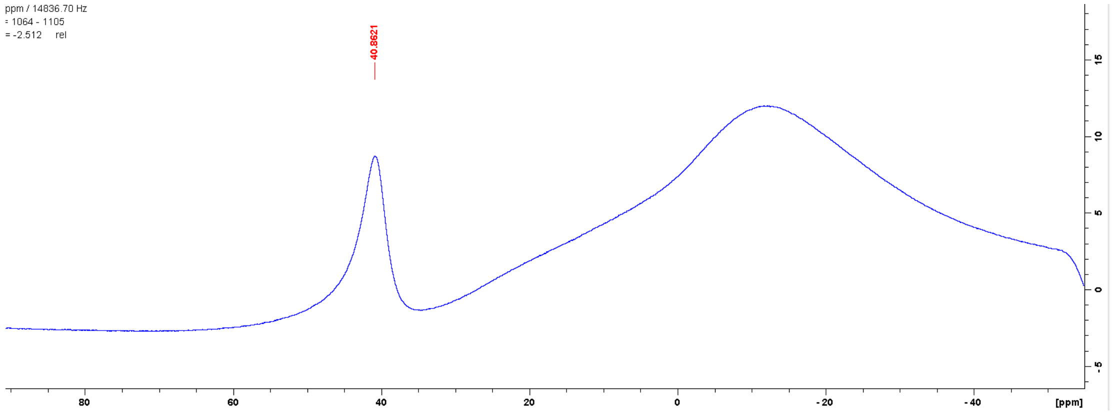

Figure S11. ^{11}B NMR spectrum of **2a** in C_6D_6.

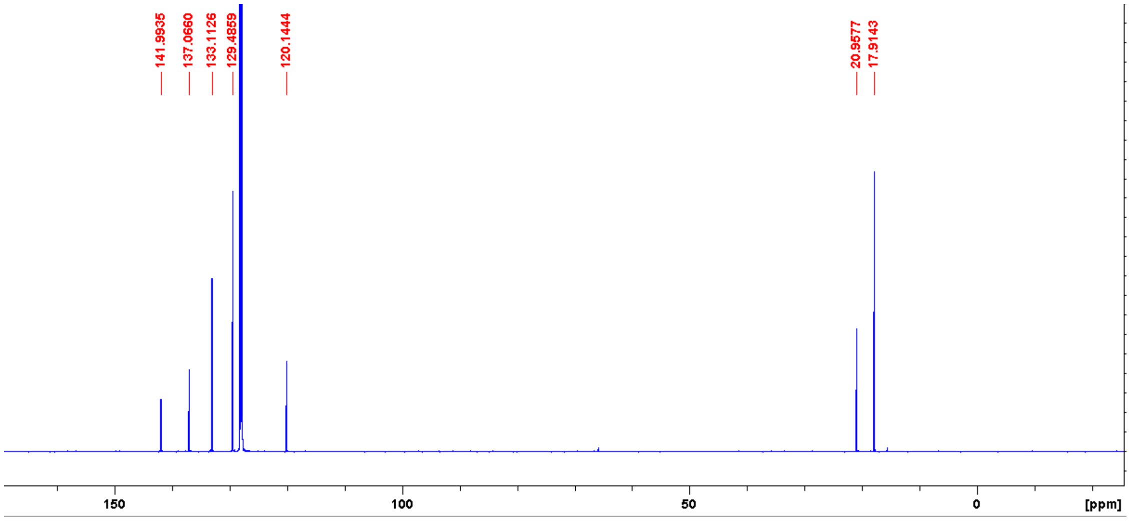

Figure S12. ^{13}C NMR spectrum of **2a** in C_6D_6.

Synthesis and characterization of 2b

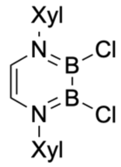

A solution of **1b** (411 mg, 1.10 mmol) in Et_2O (15 mL) was cooled to -78 °C and treated dropwise with ethereal HCl (1.30 mL, 160 mg, 4.39 mmol, 3.4 mol/L, 4 eq.), whereupon the formation of a colorless precipitate was observed. The reaction mixture was allowed to warm to room temperature overnight, and all volatiles were removed *in vacuo*. The residue was extracted into Et_2O, concentrated, and stored at -30 °C overnight to afford block-shaped colorless crystals. The mother liquor was removed, the crystals washed with cold Et_2O and dried *in vacuo* to yield 1,4-bis(2,6-dimethylphenyl)-2,3-dichloro-1,4-diaza-2,3-diborinine (**2b)** (38.0 mg, 106 µmol, 10%). Repeated crystallizations from the mother liquor increased the yields up to 36% (142 mg, 392 µmol). Crystals suitable for X-ray diffraction were obtained from saturated Et_2O solutions at -30 C.

^{1}H-NMR (500 MHz, C_6D_6): δ = 7.02-6.94 (m, 6H, aryl-C*H*), 5.49 (s, 2H, HC=C*H*), 2.02 (s, 12H, C*H*3). **^{11}B-NMR** (160 MHz, C_6D_6): δ = 40.5 (s, br, $\omega_{1/2}$ = 822). **^{13}C{^{1}H}-NMR** (125 MHz, C_6D_6): δ = 144.39 (s, *ipso*-C_q), 133.53 (s, o*rtho*-C_q), 128.78 (s, *meta*-CH), 127.78 (s, *para*-CH), 119.82 (s, HC=*C*H), 17.95 (s, *ortho*-CH_3). **El. Anal.** [%]: calculated for $C_{18}H_{20}B_2Cl_2N_2$ (356.89 g·mol^{-1}): C 60.58, H 5.65, N 7.85; found: C 60.85, H 5.58, N 7.58.

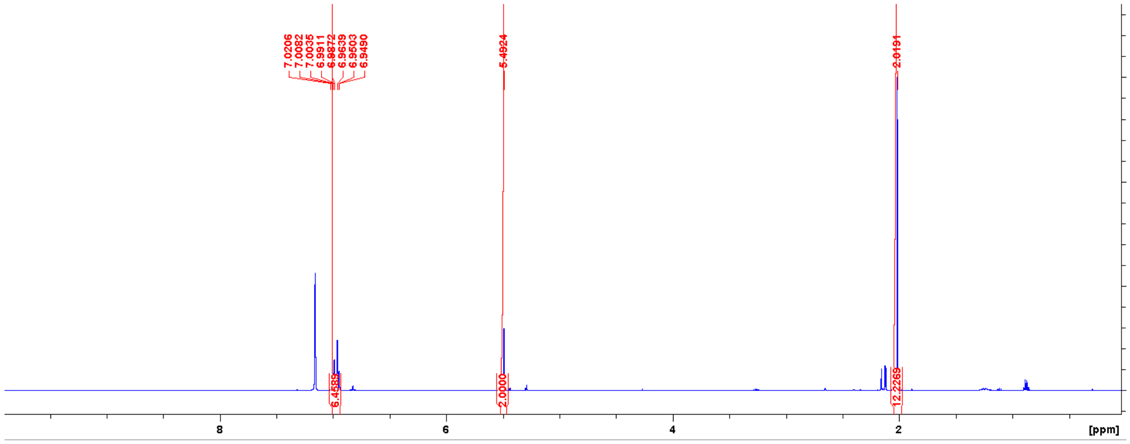

Figure S13. ^{1}H NMR spectrum of **2b** in C_6D_6.

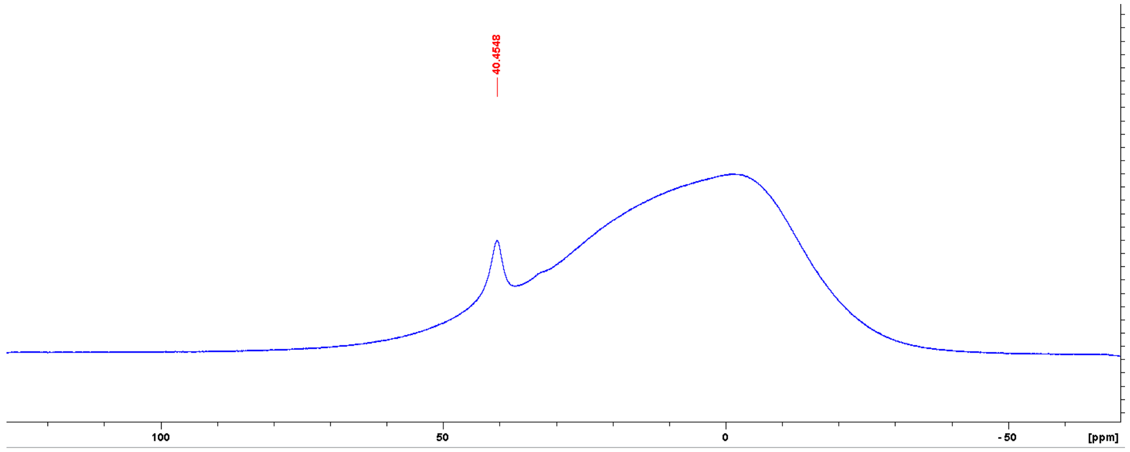

Figure S14. ^{11}B NMR spectrum of **2b** in C_6D_6.

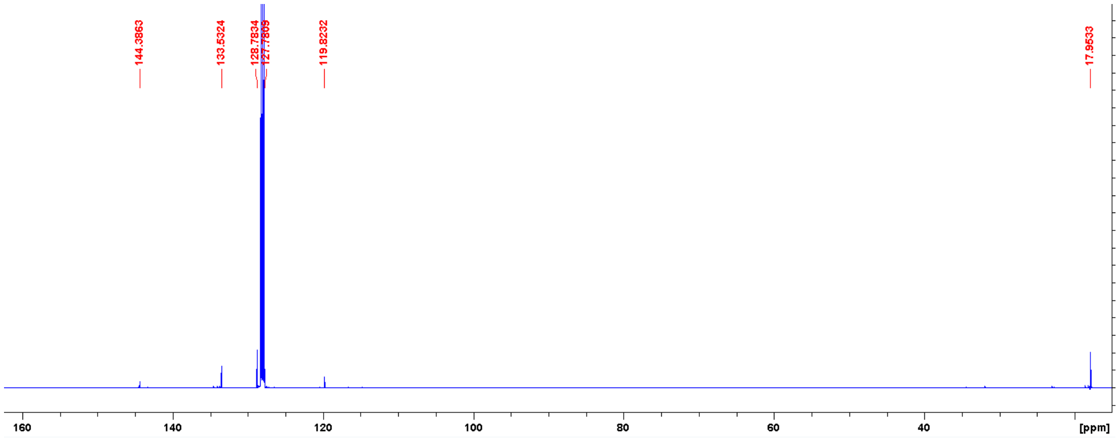

Figure S15. ^{13}C NMR spectrum of **2b** in C_6D_6.

Synthesis and characterization of 2c

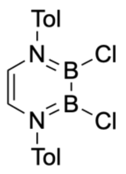

A solution of **1c** (458 mg, 1.32 mmol) in Et_2O (20 mL) was cooled to -78 °C and treated dropwise with ethereal HCl (1.56 ml, 193 mg, 5.29 mmol, 3.4 mol/L, 4 eq.), whereupon the formation of a colorless precipitate was observed. The reaction mixture was allowed to warm to room temperature overnight, and all volatiles were removed *in vacuo*. The residue was extracted into Et_2O, concentrated, and stored at -30 °C overnight to afford block-shaped colorless crystals. The mother liquor was removed, the crystals washed with cold Et_2O and dried *in vacuo* to yield 1,4-bis(*para*-tolyl)-2,3-dichloro-1,4-diaza-2,3-diborinine (**2c**) (263 mg, 801 µmol, 60%). Crystals suitable for X-ray diffraction were obtained from saturated Et_2O solutions at -30 C.

^{1}H-NMR (500 MHz, C_6D_6): δ = 6.95-6.89 (m, 8H, aryl-C*H*), 5.89 (s, 2H, CH=C*H*), 2.06 (s, 6H, *para*-CH_3). **^{11}B{^{1}H}-NMR** (160 MHz, C_6D_6): δ = 40.5 (s, br, $\omega_{1/2}$ = 1010). **^{13}C{^{1}H}-NMR** (125 MHz, C_6D_6): δ = 143.51 (s, *ipso*-C_q), 136.99 (s, *para*-C_q), 129.90 (s, *meta*-*C*H), 126.32 (s, *ortho*-*C*H), 120.33 (s, CH=*C*H), 20.89 (s, *para*-*C*H_3). **El. Anal. [%]:** calculated for $C_{16}H_{16}B_2Cl_2N_2$ (328.84 g·mol^{-1}): C 58.44, H 4.90, N 8.52; found: C 58.77, H 4.95, N 8.45.

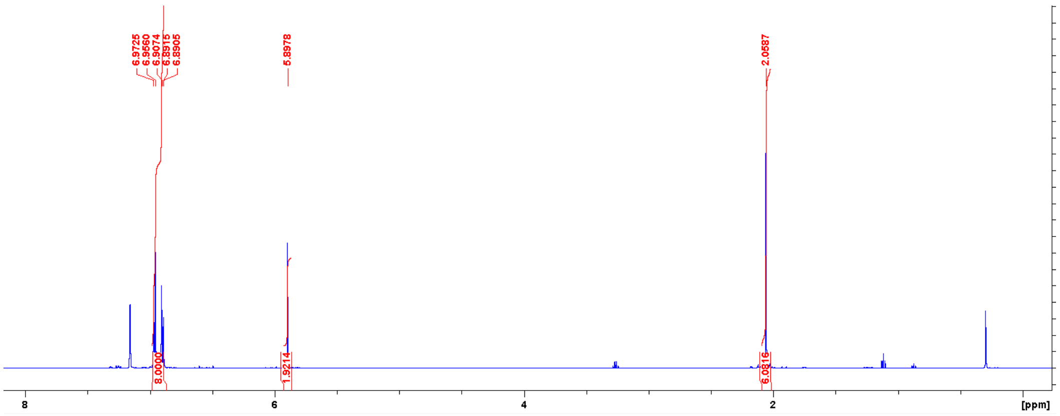

Figure S16. ^{1}H NMR spectrum of **2c** in C_6D_6.

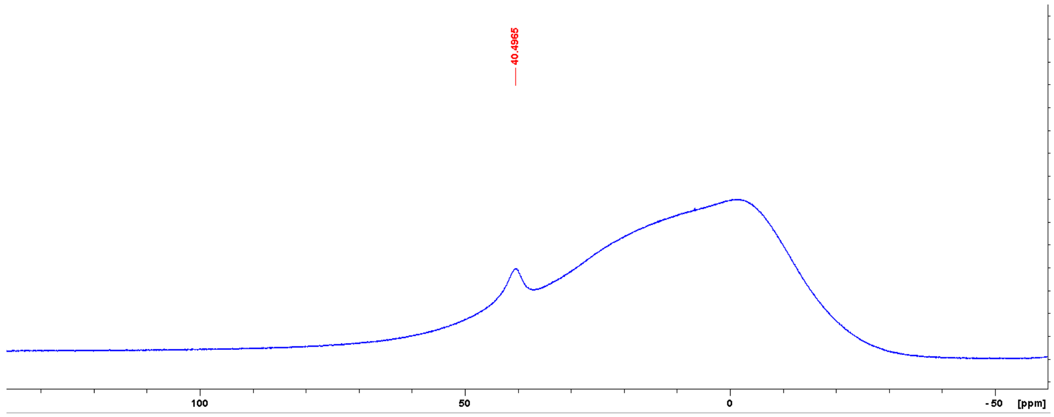

Figure S17. ^{11}B NMR spectrum of **2c** in C_6D_6.

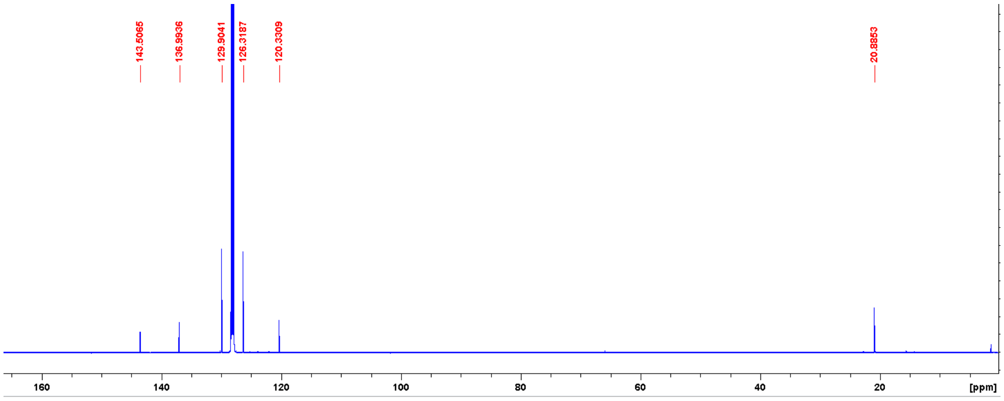

Figure S18. ^{13}C NMR spectrum of **2c** in C_6D_6.

Synthesis and characterization of 3a

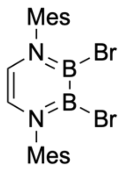

1a (1.58 g, 3.93 mmol) was dissolved in pentane (100 mL), cooled to -78 °C and treated with BBr_3 (1.97 g, 7.86 mmol, 0.75 mL, 2 eq.) in pentane (50 mL). The reaction mixture was allowed to warm to room temperature overnight, which was accompanied by the formation of a colorless precipitate. All volatiles were removed *in vacuo*, the residue was extracted into Et_2O, concentrated, and stored at -30 °C overnight to afford block-shaped colorless crystals. The mother liquor was removed, the crystals washed with cold Et_2O and dried *in vacuo* to yield 1,4-dimesityl-2,3-dibromo-1,4-diaza-2,3-diborinine (**3a**) (1.51 g, 3.19 mmol, 81%). Crystals suitable for X-ray diffraction were obtained from saturated Et_2O solutions at -30 C.

^{1}H-NMR (500 MHz, C_6D_6): δ = 6.77 (s, 4H, aryl-C*H*), 5.67 (s, 2H, C*H*), 2.12 (s, 6H, *para*-CH_3), 2.03 (s, 12H, *orhto*-CH_3). **^{11}B{^{1}H}-NMR** (160 MHz, C_6D_6): δ = 42.0 (s, br, $\omega_{1/2}$ = 847). **^{13}C{^{1}H}-NMR** (125 MHz, C_6D_6): δ = 143.4 (s, *ipso*-C_q), 137.2 (s, *para*-C_q), 132.9 (s, *ortho*-C_q), 129.5 (s, *meta*-CH), 121.3 (s, *C*H), 21.0 (s, *para*-CH_3), 18.0 (s, *ortho*-CH_3). **El. Anal.** [%]: calculated for $C_{20}H_{24}B_2Br_2N_2$ (473.85 g·mol^{-1}): C 50.69, H 5.11, N 5.91; found: C 50.69, H 5.05, N 6.19.

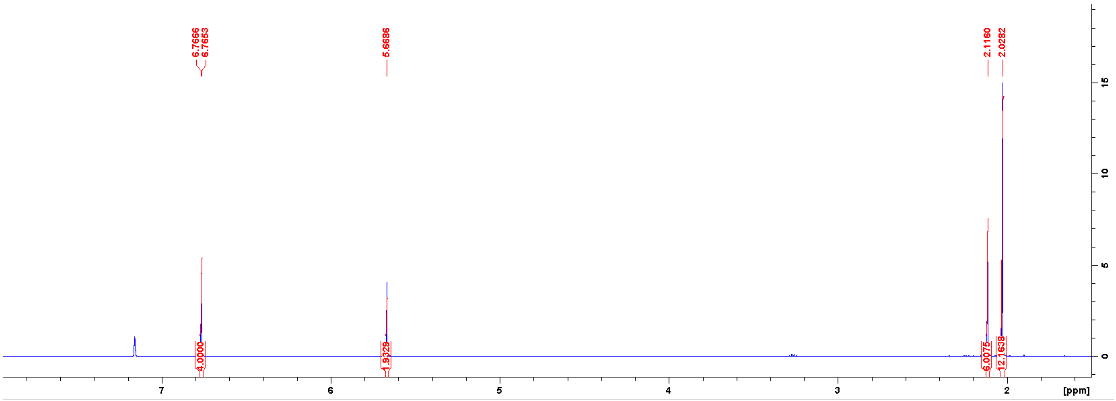

Figure S19. ^{1}H NMR spectrum of **3a** in C_6D_6.

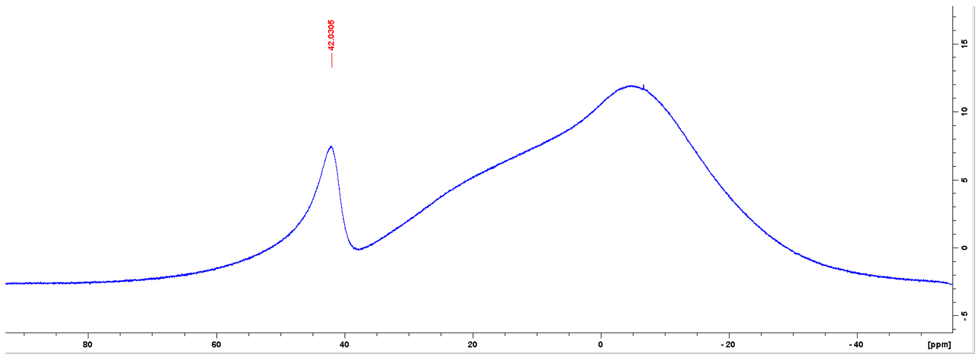

Figure S20. ^{11}B NMR spectrum of **3a** in C_6D_6.

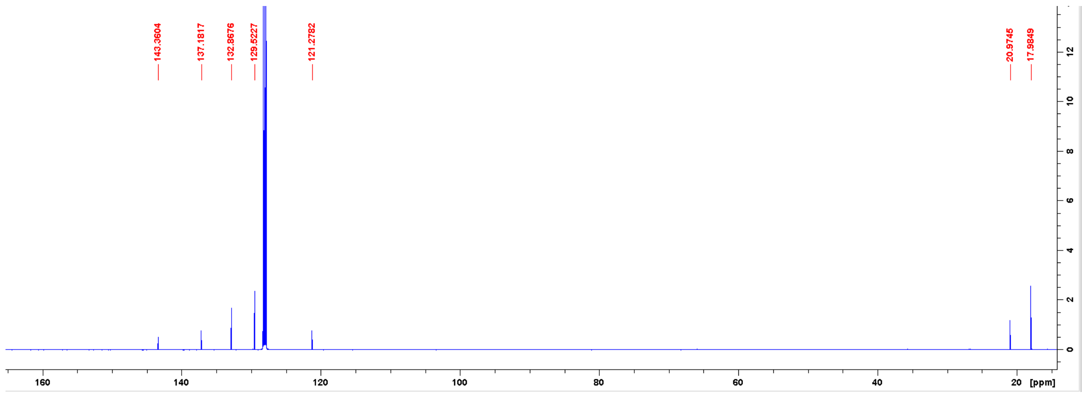

Figure S21. ^{13}C NMR spectrum of **3a** in C_6D_6.

Synthesis and characterization of 3b

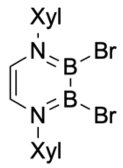

1b (228 mg, 608 µmol) was dissolved in pentane (15 mL), cooled to -78 °C and treated with BBr_3 (115 µL, 305 mg, 1.22 mmol, 2 eq.). The reaction mixture was allowed to warm to room temperature overnight, which was accompanied by the formation of a colorless precipitate. All volatiles were removed *in vacuo*, the residue was extracted into Et_2O, concentrated, and stored at -30 °C overnight to afford block-shaped colorless crystals. The mother liquor was removed, the crystals washed with cold Et_2O and dried *in vacuo* to yield 1,4-bis(2,6-dimethylphenyl)-2,3-dibromo-1,4-diaza-2,3-diborinine (**3b**) (105 mg, 236 µmol, 39%). Crystals suitable for X-ray diffraction were obtained from saturated Et_2O solutions at -30 C.

^{1}H-NMR (500 MHz, C_6D_6): δ = 7.02-6.94 (m, 6H, aryl-C*H*), 5.95 (s, 2H, HC=C*H*), 2.01 (s, 12H, C*H*$_3$). **^{11}B{^{1}H}-NMR** (160 MHz, C_6D_6): δ = 41.4 (s, br, $\omega_{1/2}$ = 462). **^{13}C{^{1}H}-NMR** (125 MHz, C_6D_6): δ = 145.70 (s, *ipso*-*C*$_q$), 133.28 (s, o*rtho*-*C*$_q$), 128.82 (s, *meta*-*C*H), 127.87 (s, *para*-*C*H), 120.97 (s, HC=*C*H), 10.03 (s, *ortho*-*C*H$_3$). **El. Anal.** [%]: calculated for $C_{18}H_{20}B_2Br_2N_2$ (445.80 g·mol^{-1}): C 48.50, H 4.52, N 6.28; found: C 48.97, H 4.53, N 5.80.

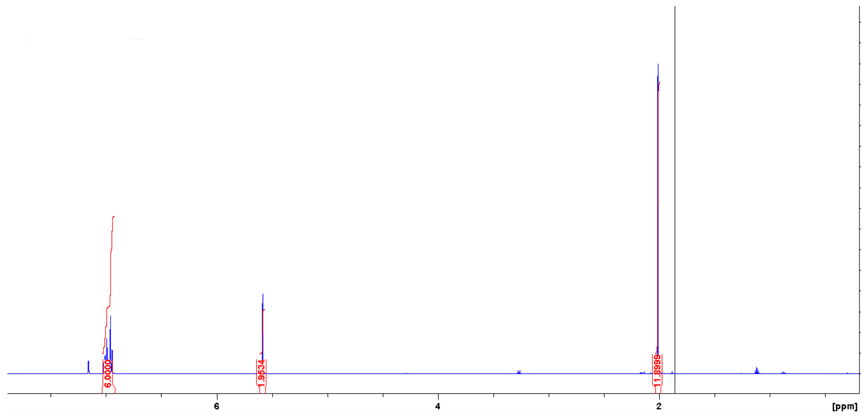

Figure S22. ^{1}H NMR spectrum of **3b** in C_6D_6.

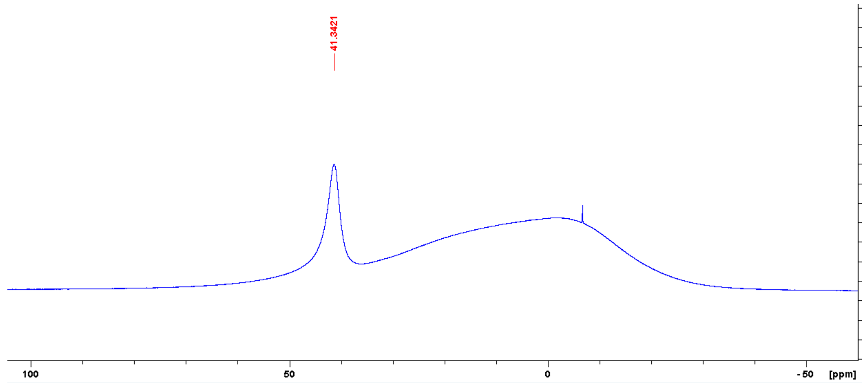

Figure S23. ^{11}B NMR spectrum of **3b** in C_6D_6.

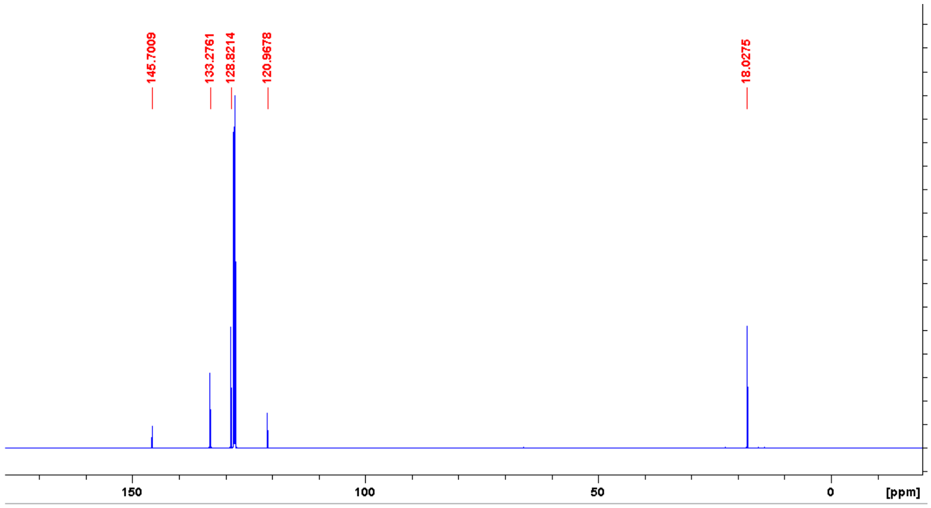

Figure S24. ^{13}C NMR spectrum of **3b** in C_6D_6.

Synthesis and characterization of 3c

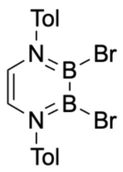

1c (185 mg, 535 µmol) was dissolved in pentane (15 mL), cooled to -78 °C and treated with BBr_3 (102 µL, 268 mg, 107 mmol, 2 eq.). The reaction mixture was allowed to warm to room temperature overnight, which was accompanied by the formation of a colorless precipitate. All volatiles were removed *in vacuo*, the residue was extracted into Et_2O, concentrated, and stored at -30 °C overnight to afford block-shaped colorless crystals. The mother liquor was removed, the crystals washed with cold Et_2O and dried *in vacuo* to yield 1,4-bis(*para*-tolyl)-2,3-dibromo-1,4-diaza-2,3-diborinine (**3c**) (47.8 mg, 114 µmol, 21%). Crystals suitable for X-ray diffraction were obtained from saturated Et_2O solutions at -30 C. The poor yields might be explained by the more reactive nature of **3c** compared to all other compounds. If the filtrate is concentrated too much, **3c** crushes out of solution and cannot be redissolved, most likely due to ether cleavage reactions.

^{1}H-NMR (500 MHz, C_6D_6): δ = 6.95-6.88 (m, 8H, aryl-C*H*), 5.95 (s, 2H, CH=C*H*), 2.04 (s, 6H, *para*-C*H*$_3$). **^{11}B{^{1}H}-NMR** (160 MHz, C_6D_6): δ = 41.1 (s, br, $\omega_{1/2}$ = 1061). **^{13}C{^{1}H}-NMR** (125 MHz, C_6D_6): δ = 144.97 (s, *ipso*-*C*$_q$), 137.20 (s, *para*-*C*$_q$), 129.93 (s, *meta*-*C*H), 126.44 (s, *ortho*-*C*H), 121.41 (s, CH=*C*H), 20.90 (s, *para*-*C*H$_3$). **El. Anal. [%]:** calculated for $C_{16}H_{16}B_2Br_2N_2$ (417.75 g·mol^{-1}): C 46.00, H 3.86, N 6.71; found: C 46.56, H 3.98, N 6.30.

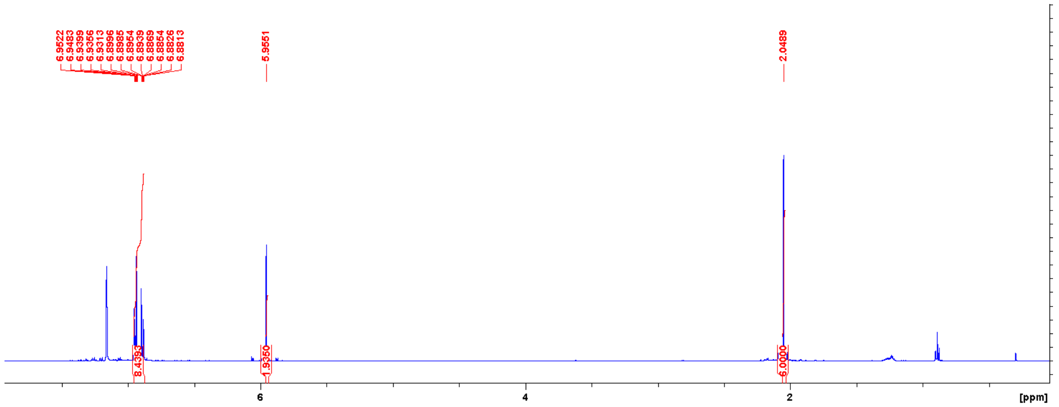

Figure S25. ^{1}H NMR spectrum of **3c** in C_6D_6.

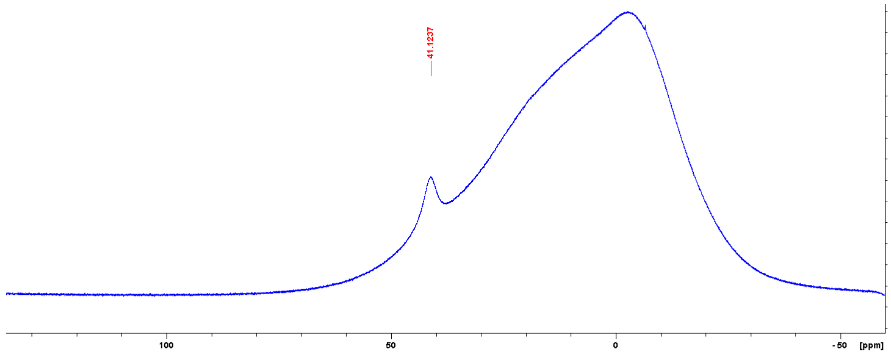

Figure S26. ^{11}B NMR spectrum of **3c** in C_6D_6.

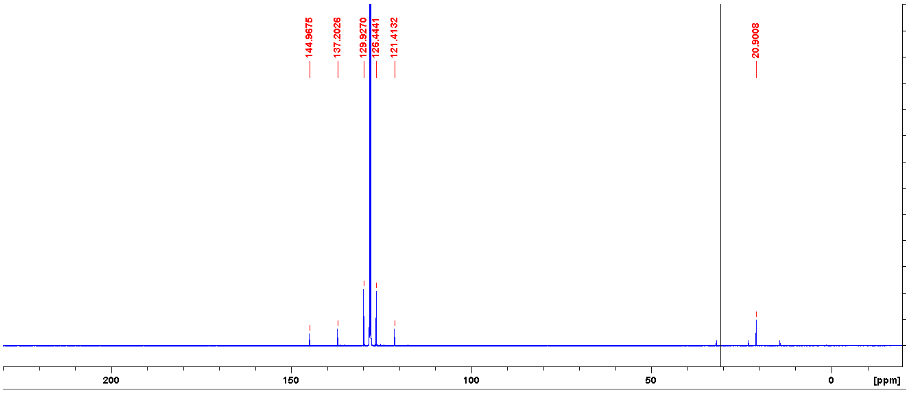

Figure S27. ^{13}C NMR spectrum of **3c** in C_6D_6.

Synthesis and characterization of 4a

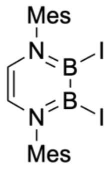

A solid mixture of **1a** (1.00 g, 2.49 mmol) and BI_3 (973.5 mg, 2.49 mmol) was suspended in precooled (-78 °C) pentane (100 mL), and the reaction mixture was allowed to warm to room temperature overnight. All volatiles were removed *in vacuo*, the residue was extracted into pentane, concentrated, and stored at -30 °C overnight to afford a colorless solid. The mother liquor was removed, the solid washed with cold pentane and dried *in vacuo* to yield 1,4-dimesityl-2,3-diiodo-1,4-diaza-2,3-diborinine (**4a**) (100 mg, 1.76 mmol, 7%). Reducing the volume of the mother liquor and storage at -30 °C afforded another crop of crystals to afford **4a** in 28% combined yields. Crystals suitable for X-ray diffraction were obtained from saturated pentane solutions at -30 C.

^{1}**H-NMR** (500 MHz, C_6D_6): δ = 6.76 (s, 4H, aryl-C*H*), 5.82 (s, 2H, C*H*), 2.14 (s, 6H, *para*-CH_3), 2.01 ppm (s, 12H, *orhto*-CH_3). ^{11}**B-NMR** (160 MHz, C_6D_6): δ = 41.4 (s, br, $\omega_{1/2}$ = 1027). ^{13}**C{**^{1}**H}-NMR** (125 MHz, C_6D_6): δ = 146.1 (s, *ipso*-C_q), 137.3 (s, *para*-C_q), 132.5 (s, *ortho*-C_q), 129.6 (s, *meta*-CH), 122.7 (s, CH), 21.0 (s, *para*-CH_3), 18.2 ppm (s, *ortho*-CH_3). **El. Anal. [%]**: calculated for $C_{20}H_{24}B_2N_2I_2$ (568.02·mol^{-1}): C 42.30, H 4.26, N 4.93; found: C 42.68, H 4.46, N 4.92.

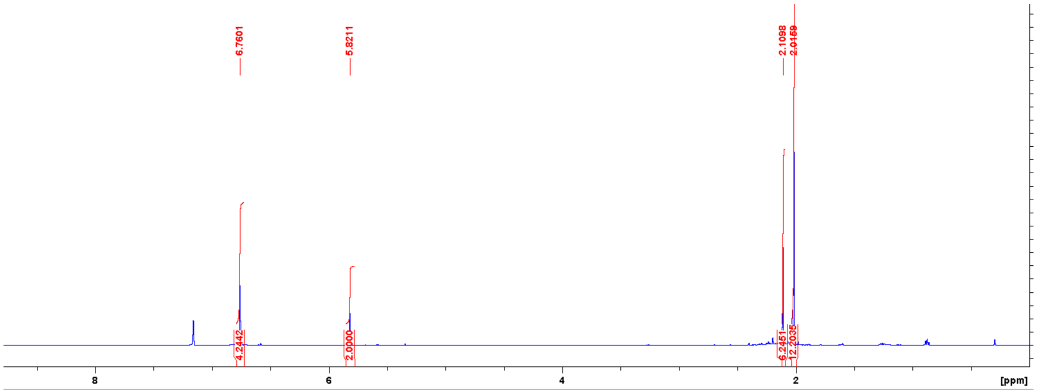

Figure S28. ^{1}H NMR spectrum of **4a** in C_6D_6.

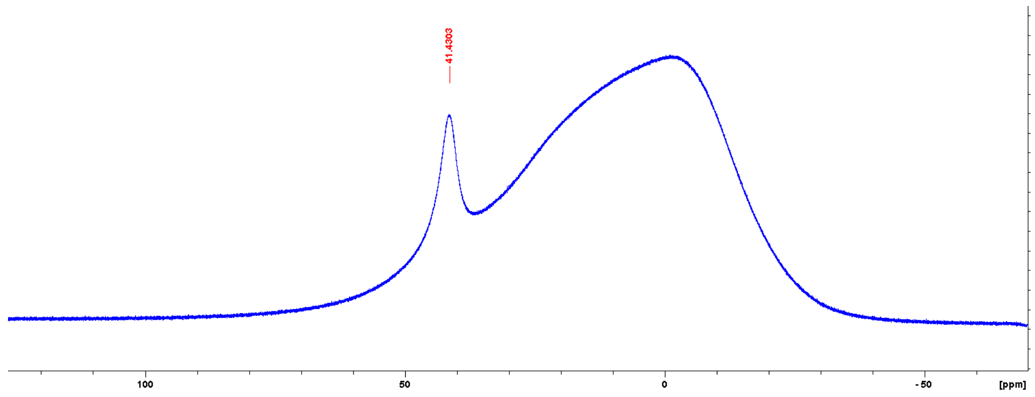

Figure S29. ^{11}B NMR spectrum of **4a** in C_6D_6.

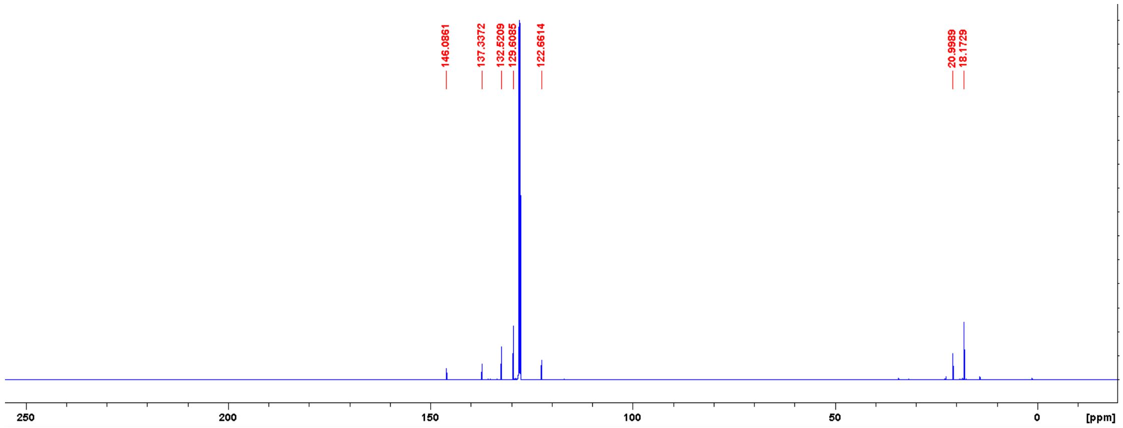

Figure S30. ^{13}C NMR spectrum of **4a** in C_6D_6.

Synthesis and characterization of 5a

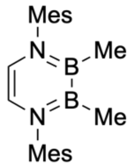

A solution of **3a** (300 mg, 633 mmol) in Et_2O (50 mL) was cooled to -78 °C and treated dropwise with ethereal MeLi (0.58 mL, 27.8 mg, 1.27 mmol, 2.2 mol/L, 2 eq.), during which time the formation of a colorless precipitate was observed. The reaction mixture was allowed to warm to room temperature overnight, and all volatiles were removed *in vacuo*. The residue was extracted into pentane, concentrated, and stored at -30 °C overnight to afford block-shaped colorless crystals. The mother liquor was removed, the crystals washed with cold pentane and dried *in vacuo* to yield 1,4-dimesityl-2,3-dimethyl-1,4-diaza-2,3-diborinine (**5a**) (113 mg, 328 µmol, 52%). Crystals suitable for X-ray diffraction were obtained from saturated pentane solutions at -30 C.

^{1}H-NMR (500 MHz, C_6D_6): δ = 6.83 (m, 2H, *aryl*-C*H*), 5.77 (s, 2H, C=C*H*), 2.17, (s, 6H, *para*-CH_3), 2.01 (s, 12H, *ortho*-CH_3), 0.72 (s br, 6H, BCH_3). **^{11}B-NMR** (160 MHz, C_6D_6; $\omega_{1/2}$ = 1220): δ = 49.0 (s, br). **^{13}C{^{1}H}-NMR** (125 MHz, C_6D_6): δ = 144.66 8 (s, *ipso*-C_q), 135.71 (s, *aryl*-C_q),

133.27 (s, *aryl*-C_q), 129.28 (s, *aryl*-C_q), 120.04 (s, H*C*=CH), 21.00 (s, *para*-C*H*$_3$), 18.06 (s, *ortho*-C*H*$_3$), 4.25 (s, BC*H*$_3$). **El. Anal. [%]**: calculated for $C_{22}H_{30}B_2N_2$ (344.12 g·mol^{-1}): C76.79, H 8.79, N 8.14; found: C 76.51, H 8.72, N 7.95.

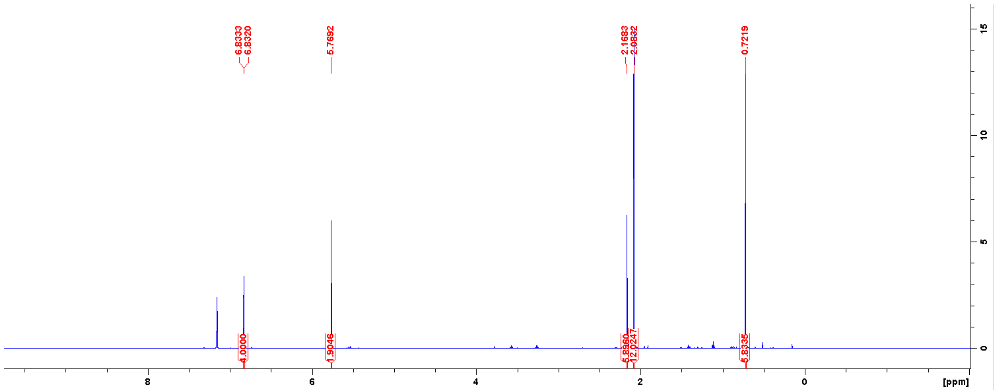

Figure S31. ^{1}H NMR spectrum of **5a** in C_6D_6.

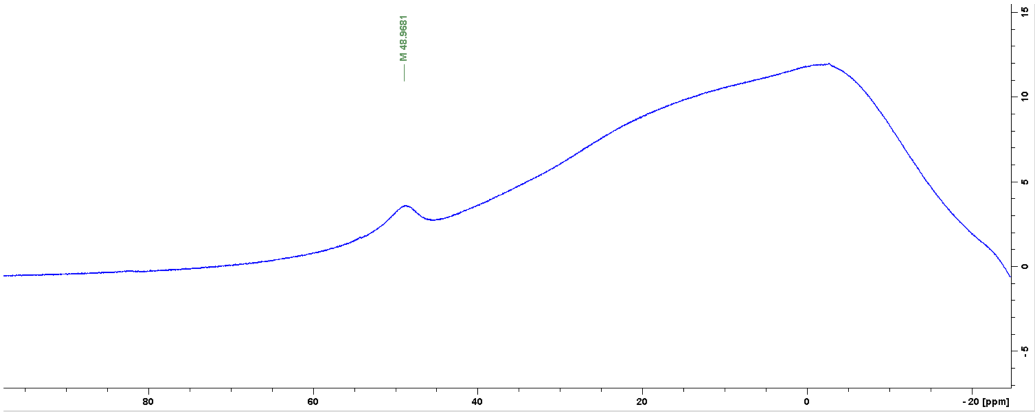

Figure S32. ^{11}B NMR spectrum of **5a** in C_6D_6.

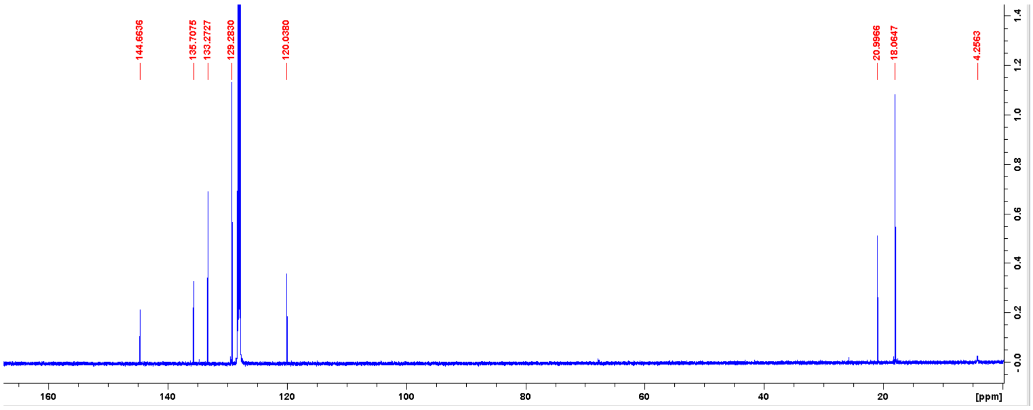

Figure S33. ^{13}C NMR spectrum of **5a** in C_6D_6.

Synthesis and characterization of 5b

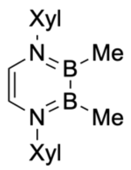

A solution of **3b** (43.3 mg, 97.1 µmol) in Et_2O (15 mL) was cooled to -78 °C and treated dropwise with ethereal MeLi (0.09 mL, 4.29 mg, 194 µmol, 2 eq.), during which time the formation of a colorless precipitate was observed. The reaction mixture was allowed to warm to room temperature overnight, and all volatiles were removed *in vacuo*. The residue was extracted into pentane, concentrated, and stored at -30 °C overnight to afford block-shaped colorless crystals. The mother liquor was removed, the crystals washed with cold pentane and dried *in vacuo* to yield 1,4-bis(2,6-dimethylphenyl)-2,3-dimethyl-1,4-diaza-2,3-diborinine (**5b**) (12.6 mg, 39.8 µmol, 41%). Crystals suitable for X-ray diffraction were obtained from saturated pentane solutions at -30 C.

^{1}H-NMR (500 MHz, C_6D_6): δ = 7.02-6.94 (m, 6H, aryl-C*H*), 5.49 (s, 2H, HC=C*H*), 2.02 (s, 12H, C*H*$_3$). **^{11}B-NMR** (160 MHz, C_6D_6): δ = 40.5 (s, br, $\omega_{1/2}$ = 1112). **^{13}C{^{1}H}-NMR** (125 MHz, C_6D_6): δ = 144.39 (s, *ipso*-*C*$_q$), 133.53 (s, o*rtho*-*C*$_q$), 128.78 (s, *meta*-*C*H), 127.78 (s, *para*-*C*H), 119.82 (s, HC=*C*H), 17.95 (s, *ortho*-*C*H$_3$).

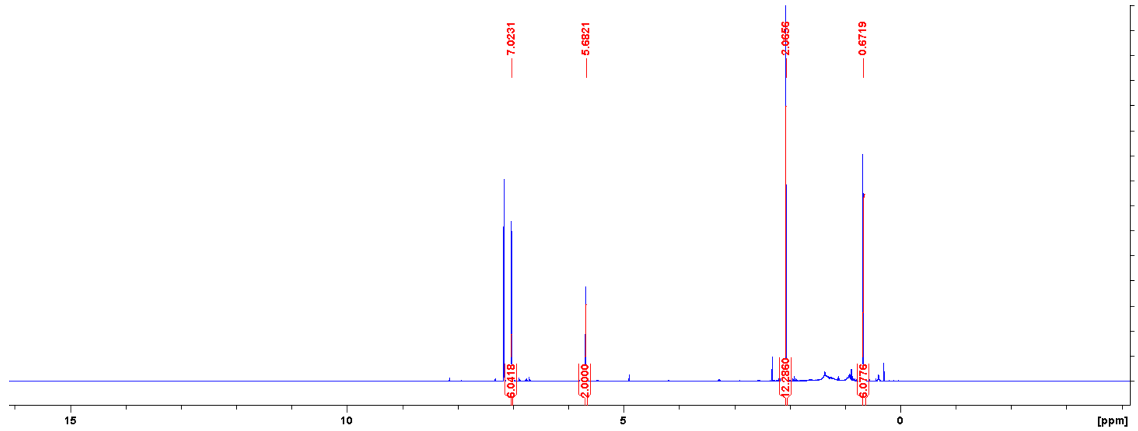

Figure S34. ^{1}H NMR spectrum of **5b** in C_6D_6.

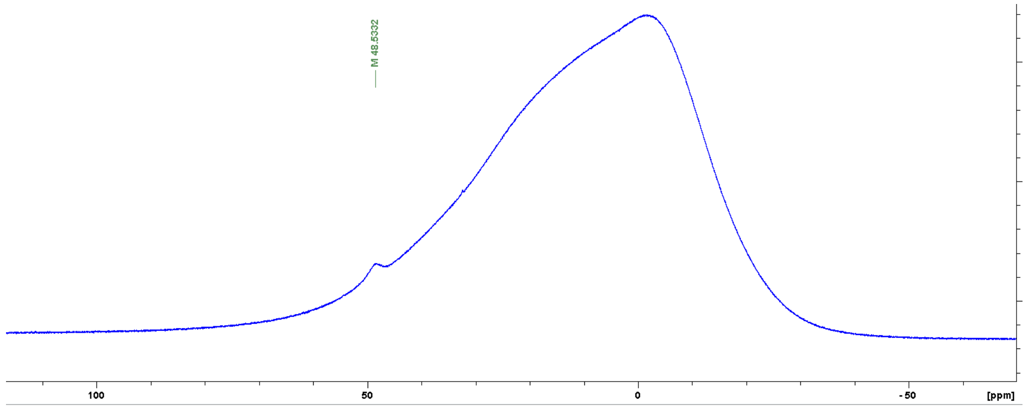

Figure S35. ^{11}B NMR spectrum of **5b** in C_6D_6.

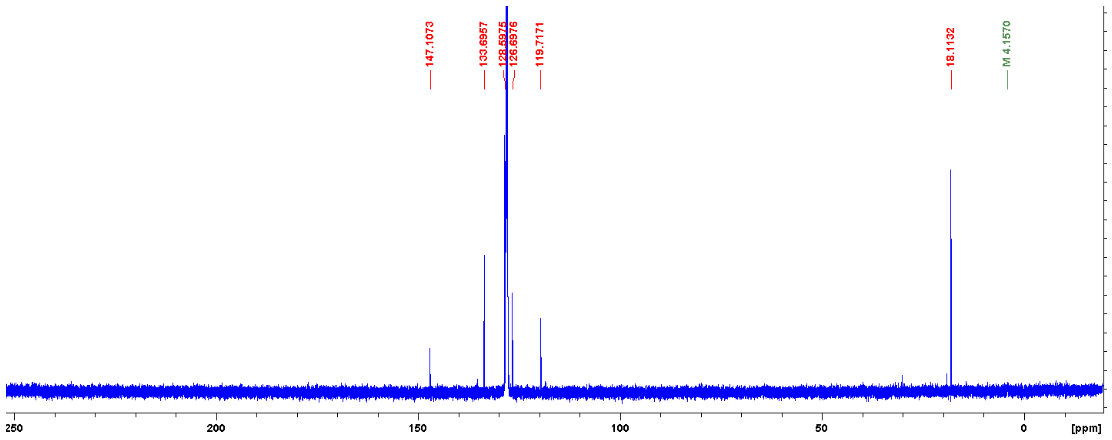

Figure S36. ^{13}C NMR spectrum of **5b** in C_6D_6.

Generation and characterization of 6a

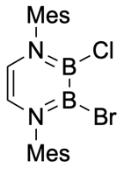

In a J. Young NMR tube, a mixture of **2a** (12.5 mg, 32.5 µmol) and **3a** (15.4 mg, 32.5 µmol) was dissolved in C_6D_6 (0.5 mL), and stored at ambient temperatures for 16 hours. ^{1}H NMR spectroscopy showed the formation of a 1:1:2 equilibrium mixture of **2a**:**3a**:**6a** (**6a** = 1,4-dimesityl-2-bromo-3-chloro-1,4-diaza-2,3-diborinine). All attempts to separate **6a** failed.

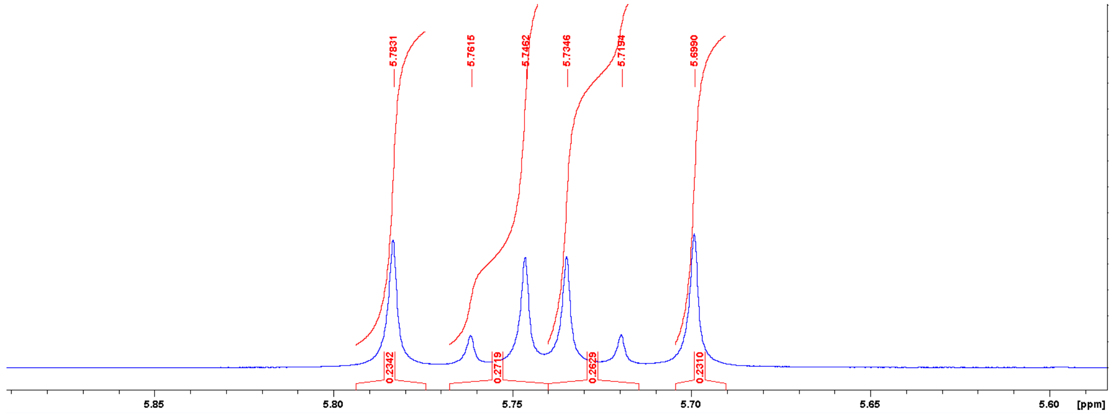

Figure S37. ^{1}H NMR spectrum of the backbone region of the statistical **2a**/**3a**/**6a** mixture in C_6D_6.

Generation and characterization of 6c

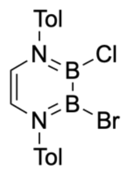

In a J. Young NMR tube, a mixture of **2c** (7.9 mg, 24.0 µmol) and **3c** (10.0 mg, 24.0 µmol) was dissolved in C_6D_6 (0.5 mL), and stored at ambient temperatures for 16 hours. ^{1}H NMR spectroscopy showed the formation of a 1:1:2 equilibrium mixture of **2c**:**3c**:**6c** (**6c** = 1,4-bis(*para*-tolyl)-2-bromo-3-chloro-1,4-diaza-2,3-diborinine). All attempts to separate **6c** failed. The sample was dried *in vacuo*, redissolved in toluene-d_8, and subjected to VT NMR studies. Accordingly, the equilibrium is temperature-independent.

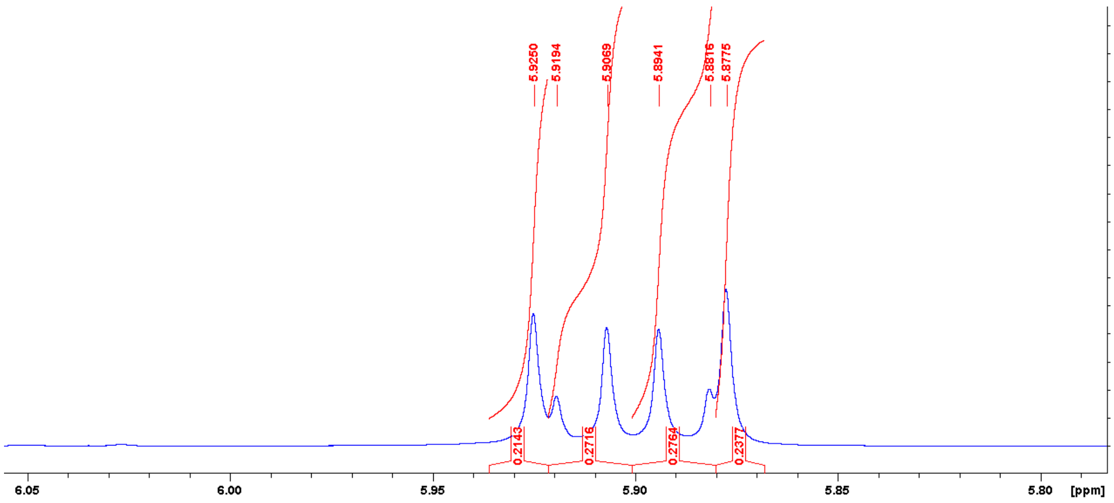

Figure S38. ^{1}H NMR spectrum of the backbone region of the statistical **2c**/**3c**/**6c** mixture in toluene-d_8 at -40 °C.

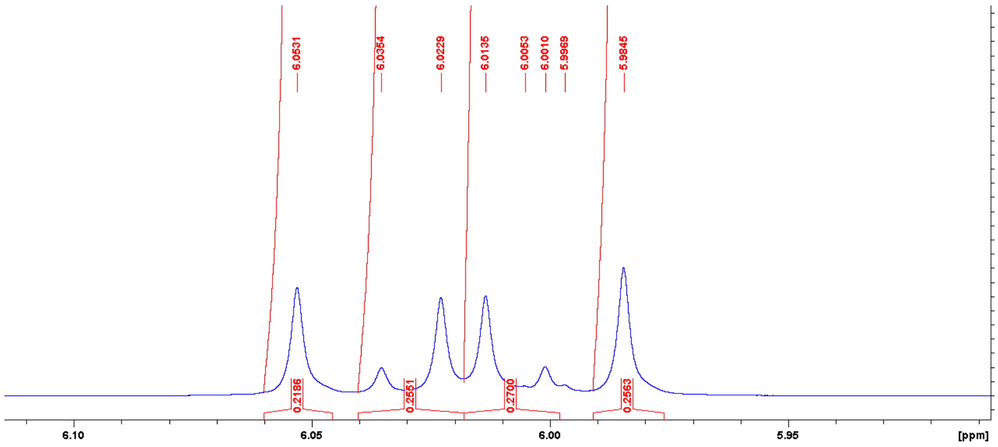

Figure S39. ^{1}H NMR spectrum of the backbone region of the statistical **2c**/**3c**/**6c** mixture in toluene-d_8 at 70 °C.

Synthesis and characterization of 7a

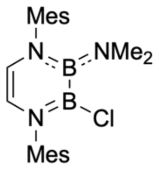

A solid mixture of **1a** (42.4 mg, 105 µmol) and **2a** (40.6 mg, 105 µmol) was dissolved in benzene (2 mL) and stirred at room temperature overnight. All volatiles were removed *in vacuo* to afford 1,4-dimesityl-2-chloro-3-dimethylamino-1,4-diaza-2,3-diborinine (**7a**) (78.3 mg, 199 µmol, 94%) analytically pure as a colorless powder. Crystals suitable for X-ray diffraction were obtained from saturated benzene solutions at room temperature.

^{1}H-NMR (500 MHz, C_6D_6): δ = 6.83 (m, 2H, aryl-C*H*), 6.77 (m, 2H, aryl-C*H*), 5.47 (d, 1H, CH=C*H*, $^{3}J_{HH}$ = 6.2 Hz,), 5.33 (d, 1H, *H*C=CH, $^{3}J_{HH}$ = 6.2 Hz,), 2.65 (br s, 6H, N(CH_3)$_2$), 2.23 (s, 6H, *ortho*-CH_3), 2.17 (s, 6H, *ortho*-CH_3), 2.15 (s, 3H, *para*-CH_3), 2.14 (s, 3H, *para*-CH_3). **^{11}B-NMR** (160 MHz, C_6D_6): δ = 41.1 (br, s, *B*Cl, $\omega_{1/2}$ = 1207), 30.7 (br, s, *B*NMe$_2$, $\omega_{1/2}$ = 1065). **^{13}C{^{1}H}-NMR** (125 MHz, C_6D_6): δ = 145.8 (s, *ipso*-C_q), 143.4 (s, *ipso*-C_q), 136.2 (s, aryl-C_q), 135.7 (s, aryl-C_q), 134.4 (s, aryl-C_q), 133.6 (s, aryl-C_q), 129.4 (s, *meta*-*C*H), 129.3 (s, *meta*-*C*H), 122.9 (s, HC=*C*H), 113.2 (s, H*C*=CH), 41.3 (br s, N(*C*H$_3$)$_2$), 21.0 (s, *para*-*C*H$_3$), 20.96 (s, *para*-*C*H$_3$), 18.6 (s, *ortho*-*C*H$_3$), 18.3 (s, *ortho*-*C*H$_3$). **El. Anal. [%]**: calculated for $C_{22}H_{30}B_2ClN_3$ (393.57 g·mol^{-1}): C 67.14, H 7.69, N 10.68; found: C 67.06, H 8.03, N 11.22.

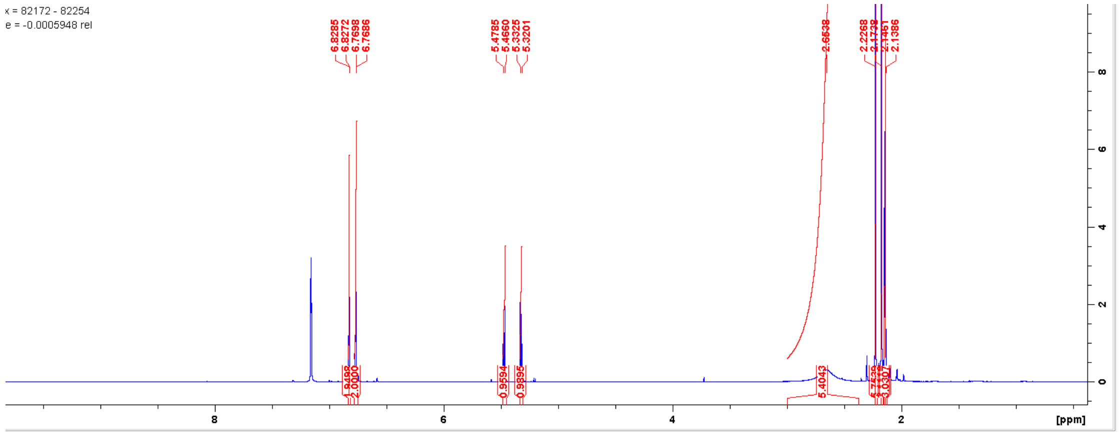

Figure S40. ^{1}H NMR spectrum of **7a** in C_6D_6.

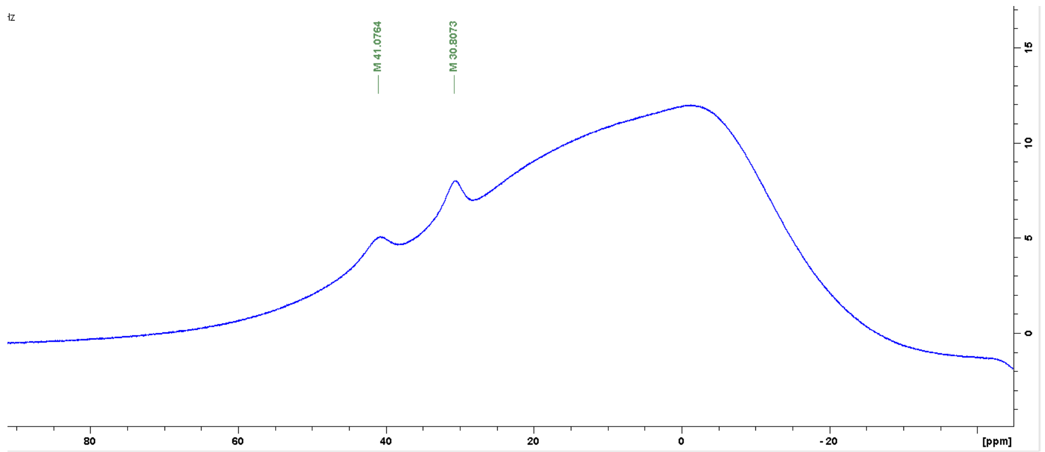

Figure S41. ^{11}B NMR spectrum of **7a** in C_6D_6.

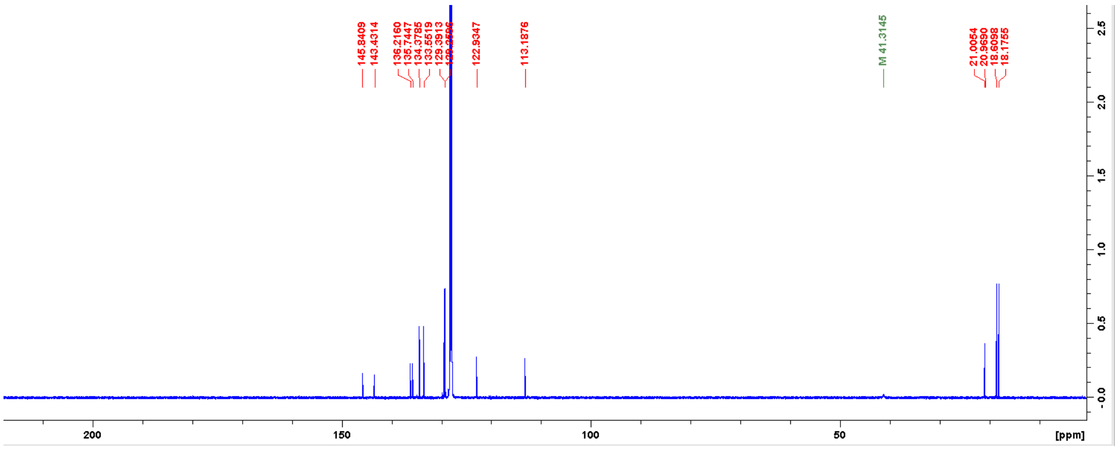

Figure S42. ^{13}C NMR spectrum of **7a** in C_6D_6.

Synthesis and characterization of 7b

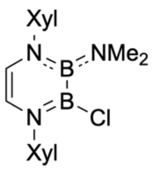

A solid mixture of **1b** (10.5 mg, 28.0 µmol) and **2b** (10.0 mg, 28.0 µmol) was dissolved in benzene (0.5 mL) in a J. Young NMR tube and allowed to react at room temperature overnight. All volatiles were removed *in vacuo* to afford 1,4-bis(2,6-dimethylphenyl)-2-chloro-3-dimethylamino-1,4-diaza-2,3-diborinine (**7b**) (12.0 mg, 32.8 µmol, 59%) analytically pure as a colorless powder. Crystals suitable for X-ray diffraction were obtained from saturated pentane solutions at -30 °C.

^{1}H-NMR (500 MHz, C_6D_6): δ = 7.02 (m, 3H, aryl-C*H*), 6.95 (m, 3H, aryl-C*H*), 5.39 (d, 1H, HC=C*H*, $^3J_{HH}$ = 6.27 Hz,), 5.24 (d, 1H, *H*C=CH, $^3J_{HH}$ = 6.27 Hz,), 2.59 (br s, 6H, N(CH_3)$_2$), 2.21 (s, 6H, *ortho*-CH_3), 2.16 (s, 6H, *ortho*-CH_3). **^{11}B-NMR** (160 MHz, C_6D_6): δ = 40.7 (br, s, *B*Cl, $\omega_{1/2}$ = 873), 30.4 (br, s, *B*N(CH$_3$)$_2$, $\omega_{1/2}$ = 821). **^{13}C{^{1}H}-NMR** (125 MHz, C_6D_6): δ = 148.29 (s, *ipso*-

C_q), 145.84 (s, *ipso*-C_q), 134.76 (s, *ortho*-C_q), 133.69 (s, *ortho*-C_q), 128.67 (s, *meta*-CH), 128.56 (s, *meta*-CH), 127.12 (s, *para*-CH), 126.59 (s, *para*-CH), 122.64 (s, HC=*C*H), 112.88 (s, H*C*=CH), 41.17 (br s, N(CH_3)$_2$), 18.64 (s, *ortho*-CH_3), 18.21 (s, *ortho*-CH_3). **El. Anal. [%]**: calculated for $C_{20}H_{26}B_2ClN_3$ (365.52 g·mol^{-1}): C 65.72, H 7.17, N 11.50; found: C 64.78, H 7.07, N 10.84.

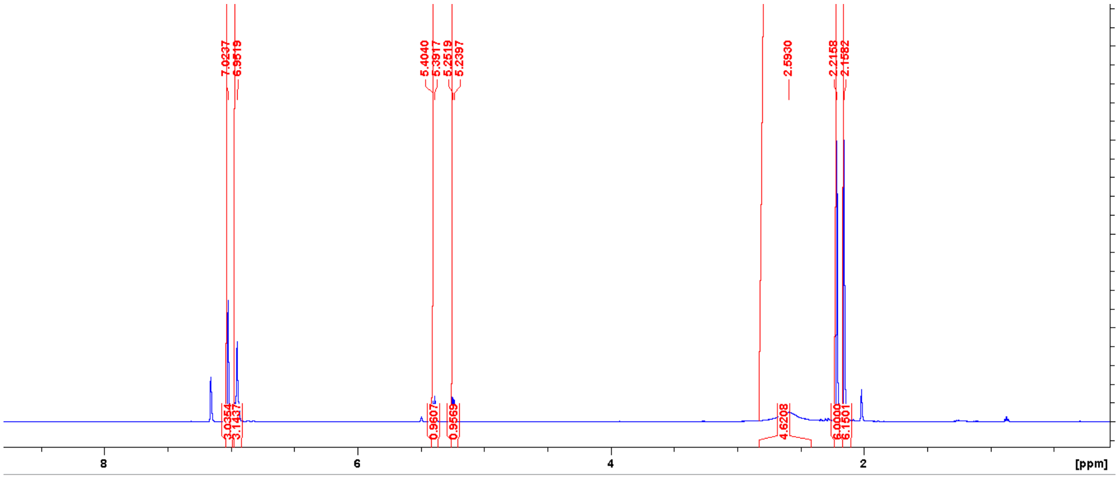

Figure S43. ^{1}H NMR spectrum of **7b** in C_6D_6.

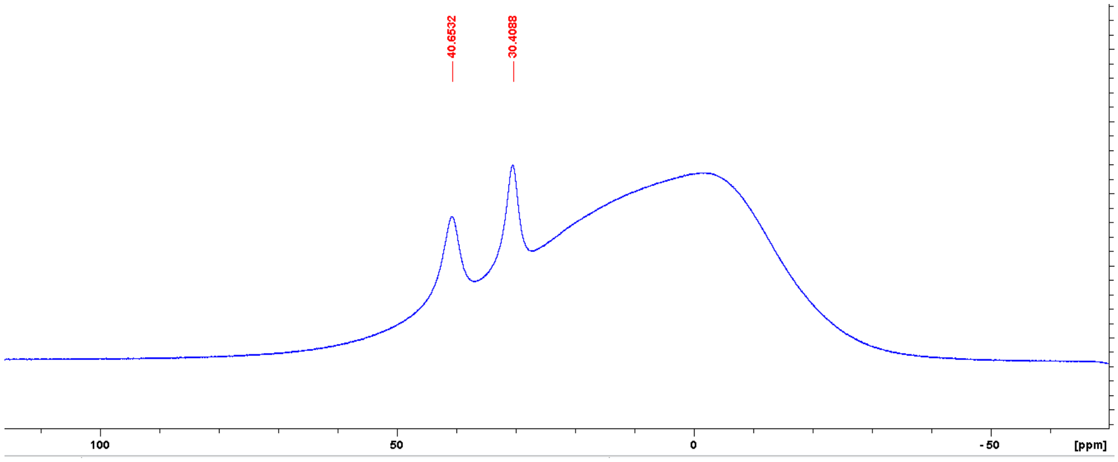

Figure S44. ^{11}B NMR spectrum of **7b** in C_6D_6.

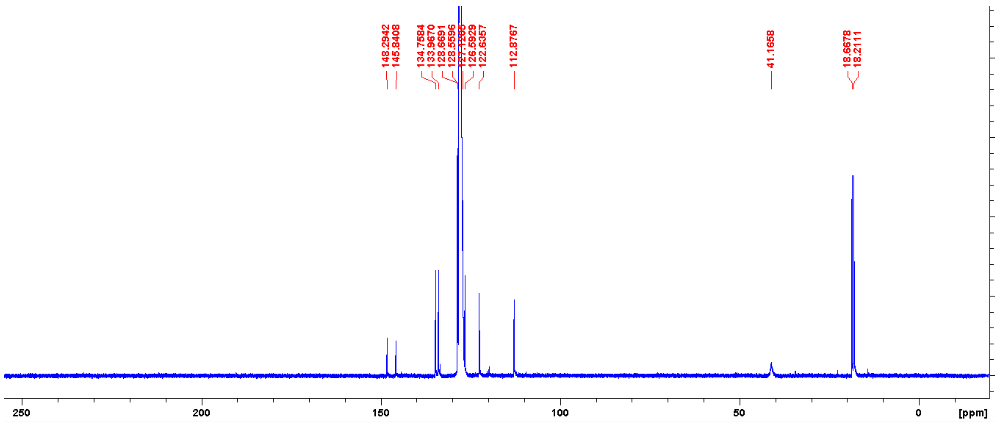

Figure S45. ^{13}C NMR spectrum of **7b** in C_6D_6.

Synthesis and characterization of 7c

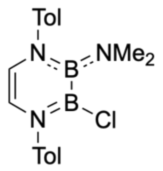

A solid mixture of **1c** (177 mg, 511 µmol) and **2c** (168 mg, 511 µmol) was dissolved in benzene (20 mL) and stirred at room temperature overnight. All volatiles were removed *in vacuo* to afford 1,4-bis(*para*-tolyl)-2-bromo-3-dimethylamino-1,4-diaza-2,3-diborinine (**7c**) (311 mg, 922 µmol, 90%) analytically pure as a colorless powder. Crystals suitable for X-ray diffraction were obtained from saturated benzene solutions at room temperature.

^{1}H-NMR (500 MHz, C_6D_6): δ = 7.10-6.88 (m, 8H, aryl-C*H*), 6.02 (d, 1H, HC=C*H*, $^{3}J_{HH}$ = 6.39 Hz,), 5.73 (d, 1H, *H*C=CH, $^{3}J_{HH}$ = 6.39 Hz,), 2.67 (br s, 6H, N(C*H*$_3$)$_2$), 2.09 (s, 6H, *para*-C*H*$_3$), 2.07 (s, 6H, *para*-C*H*$_3$). **^{11}B{^{1}H}-NMR** (160 MHz, C_6D_6): δ = 40.6 (br, s, *B*Br, $\omega_{1/2}$ = 1061), 31.6 (br, s, *B*N(CH$_3$)$_2$, $\omega_{1/2}$ = 993). **^{13}C{^{1}H}-NMR** (125 MHz, C_6D_6): δ = 148.00 (s, *ipso*-*C*$_q$), 145.00 (s, *ipso*-*C*$_q$), 136.18 (s, *para*-*C*$_q$), 134.46 (s, *para*-*C*$_q$), 130.03 (s, *meta*-*C*H), 129.83 (s, *meta*-*C*H), 127.10 (s, *ortho*-*C*H), 126.18 (s, *ortho*-*C*H), 122.41 (s, HC=*C*H), 114.93 (s, H*C*=CH), 41.95 (br s, N(*C*H$_3$)$_2$), 20.91 (s, *ortho*-C*H*$_3$), 20.85 (s, *ortho*-C*H*$_3$). **El. Anal. [%]**: calculated for $C_{18}H_{22}B_2ClN_3$ (337.47 g/mol): C 64.07, H 6.57, N 12.45; found: C 63.64, H 6.49, N 11.90.

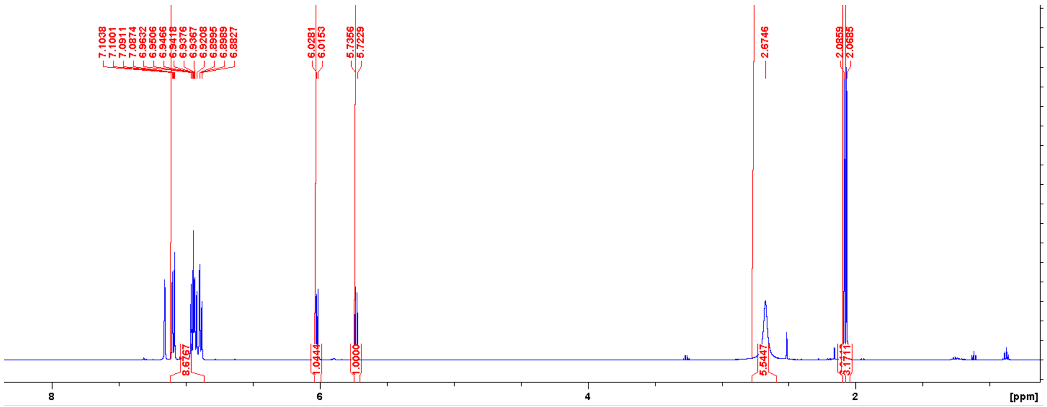

Figure S46. ^{1}H NMR spectrum of **7c** in C_6D_6.

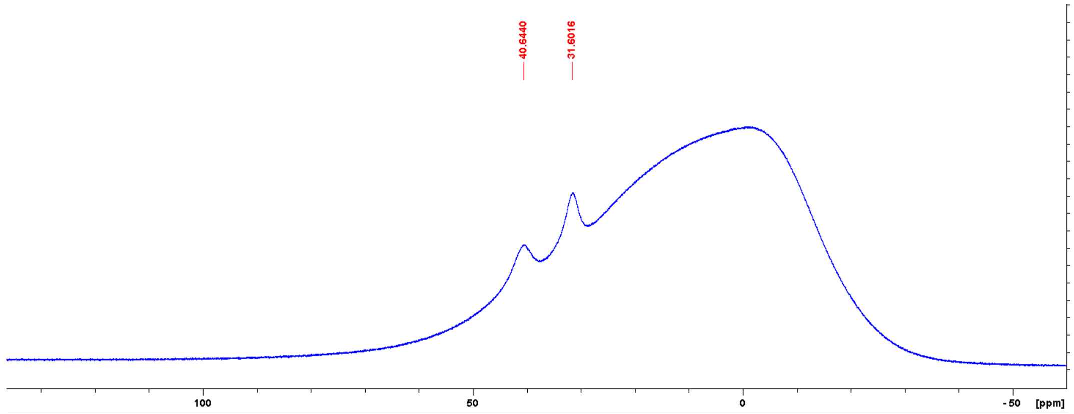

Figure S47. ^{11}B NMR spectrum of **7c** in C_6D_6.

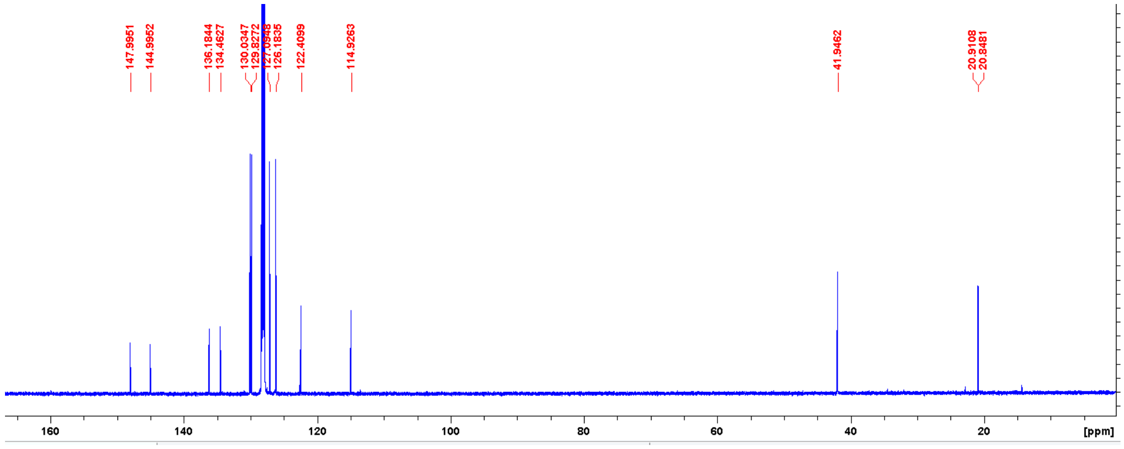

Figure S48. ^{13}C NMR spectrum of **7c** in C_6D_6.

Synthesis and characterization of 8a

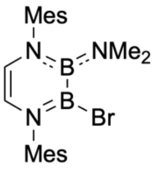

A solid mixture of **1a** (501 mg, 1.25 mmol) and **3a** (590 mg, 1.25 mmol) was dissolved in benzene (20 mL) and stirred at room temperature overnight. All volatiles were removed *in vacuo* to afford 1,4-dimesityl-2-bromo-3-dimethylamino-1,4-diaza-2,3-diborinine (**8a**) (927 mg, 2.12 mmol, 85%) analytically pure as a colorless powder. Crystals suitable for X-ray diffraction were obtained from saturated benzene solutions at room temperature.

^{1}H-NMR (500 MHz, C_6D_6): δ = 6.83 (m, 2H, aryl-C*H*), 6.76 (m, 2H, aryl-C*H*), 5.50 (d, 1H, CH=C*H*, $^3J_{HH}$ = 6.2 Hz,), 5.38 (d, 1H, *H*C=CH, $^3J_{HH}$ = 6.2 Hz,), 2.68 (br s, 6H, N(CH_3)$_2$), 2.23 (s, 6H, *ortho*-CH_3), 2.15 (s, 6H, *ortho*-CH_3), 2.15 (s, 3H, *para*-CH_3), 2.13 (s, 3H, *para*-CH_3). **^{11}B-NMR** (160 MHz, C_6D_6): δ = 40.7 (br, s, *B*Br, $\omega_{1/2}$ = 1117), 31.1 (br, s, *B*NMe$_2$, $\omega_{1/2}$ = 937). **^{13}C{^{1}H}-NMR** (125 MHz, C_6D_6): δ = 145.8 (s, *ipso*-C_q), 144.9 (s, *ipso*-C_q), 136.3 (s, aryl-C_q),

135.8 (s, aryl-C_q), 134.3 (s, aryl-C_q), 133.3 (s, aryl-C_q), 129.4 (s, *meta*-CH), 129.3 (s, *meta*-CH), 123.7 (s, HC=CH), 123.7 (s, HC=CH), 41.5 (br s, $N(CH_3)_2$), 21.0 (s, *para*-CH_3), 20.96 (s, *para*-CH_3), 18.6 (s, *ortho*-CH_3), 18.3 (s, *ortho*-CH_3). **El. Anal. [%]**: calculated for $C_{22}H_{30}B_2BrN_3$ (438.03 g·mol^{-1}): C 60.33, H 6.90, N 9.59; found: C 58.55, H 7.54, N 9.22.

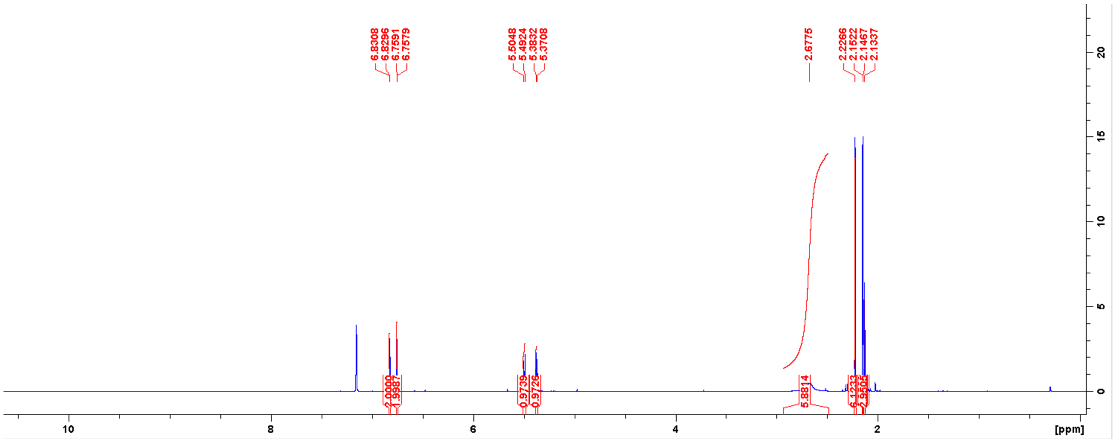

Figure S49. ^{1}H NMR spectrum of **8a** in C_6D_6.

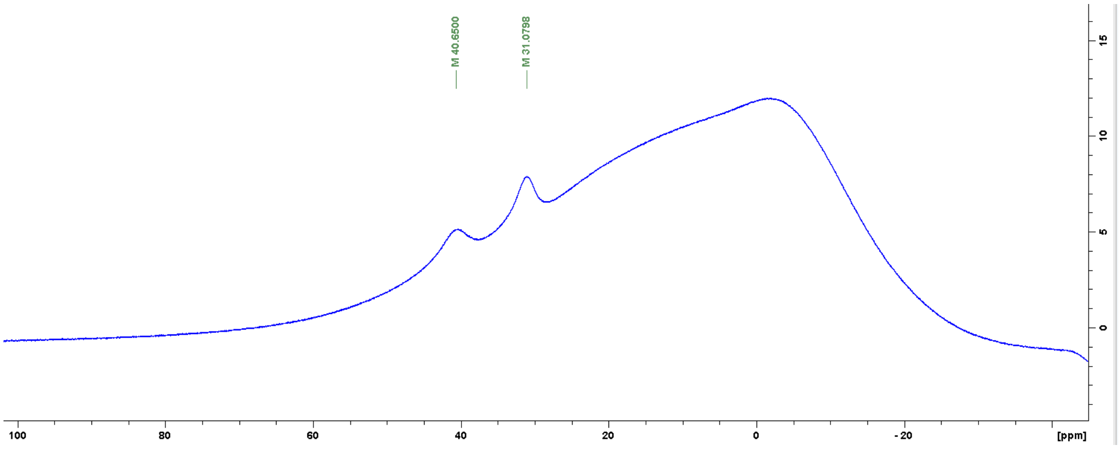

Figure S50. ^{11}B NMR spectrum of **8a** in C_6D_6.

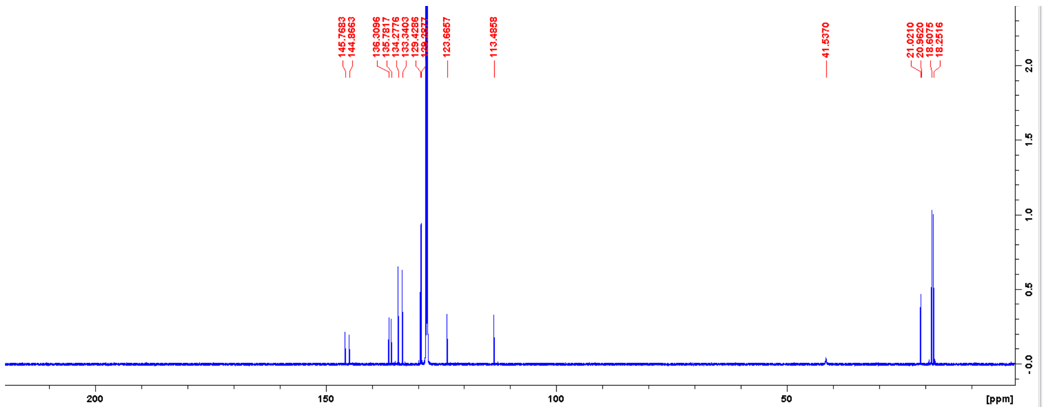

Figure S51. ^{13}C NMR spectrum of **8a** in C_6D_6.

Synthesis and characterization of 8b

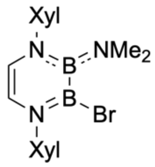

In a J. Young NMR tube, a solid mixture of **1b** (15 mg, 40.2 µmol) and **3b** (17.9 mg, 40.2 µmol) was dissolved in benzene (0.5 mL) and allowed to react at room temperature overnight. All volatiles were removed *in vacuo* to afford 1,4-bis(2,6-dimethylphenyl)-2-bromo-3-dimethylamino-1,4-diaza-2,3-diborinine (**8b**) (30.1 mg, 73.4 µmol, 91%) analytically pure as a colorless powder. Crystals suitable for X-ray diffraction were obtained from saturated benzene solutions at room temperature.

^{1}H-NMR (500 MHz, C_6D_6): δ = 7.03-7.00 (m, 3H, aryl-C*H*), 6.97-6.92 (m, 3H, aryl-C*H*), 5.42 (d, 1H, HC=C*H*, $^3J_{HH}$ = 6.22 Hz,), 5.29 (d, 1H, *H*C=CH, $^3J_{HH}$ = 6.22 Hz,), 2.63 (br s, 6H, N(C*H*$_3$)$_2$), 2.21 (s, 6H, *ortho*-C*H*$_3$), 2.14 (s, 6H, *ortho*-C*H*$_3$). **^{11}B{^{1}H}-NMR** (160 MHz, C_6D_6): δ = 40.4 (br, s, *B*Br, $\omega_{1/2}$ = 1044), 31.0 (br, s, *B*N(CH$_3$)$_2$, $\omega_{1/2}$ = 907). **^{13}C{^{1}H}-NMR** (125 MHz, C_6D_6): δ = 148.23 (s, *ipso*-*C*$_q$), 147.24 (s, *ipso*-*C*$_q$), 134.66 (s, o*rtho*-*C*$_q$), 133.76 (s, o*rtho*-*C*$_q$), 128.71 (s, *meta*-*C*H), 128.59 (s, *meta*-*C*H), 127.19 (s, *para*-*C*H), 126.62 (s, *para*-*C*H), 123.36 (s, HC=*C*H), 113.18 (s, H*C*=CH), 41.46 (br s, N(*C*H$_3$)$_2$), 18.67 (s, *ortho*-*C*H$_3$), 18.29 (s, *ortho*-*C*H$_3$). **El. Anal. [%]**: calculated for $C_{20}H_{26}B_2BrN_3$ (409.97 g·mol^{-1}): C 58.59, H 6.39, N 10.25; found: C 58.69, H 6.45, N 10.14.

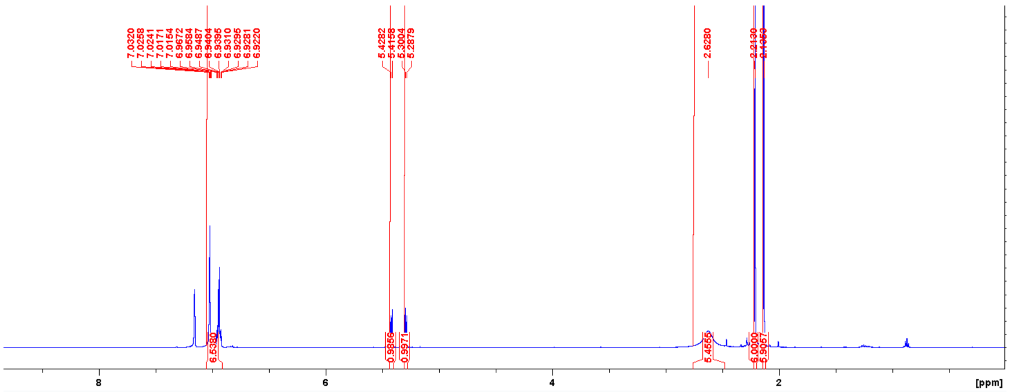

Figure S52. ^{1}H NMR spectrum of **8b** in C_6D_6.

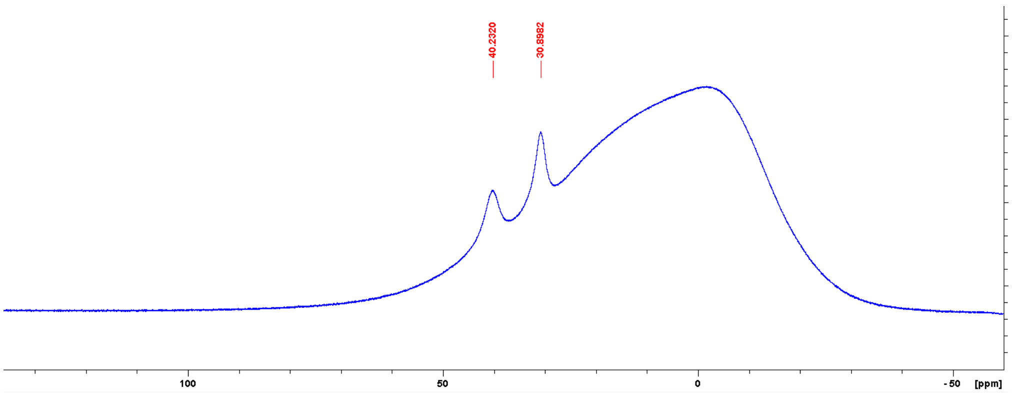

Figure S53. ^{11}B NMR spectrum of **8b** in C_6D_6.

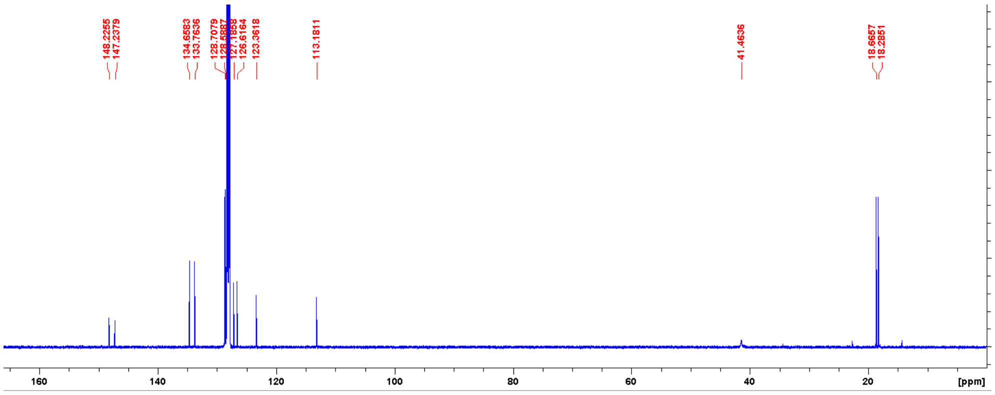

Figure S54. ^{13}C NMR spectrum of **8b** in C_6D_6.

Synthesis and characterization of 8c

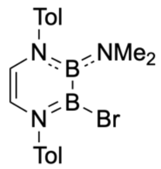

In a J. Young NMR tube, a solid mixture of **1c** (20 mg, 57.8 µmol) and **3c** (24.1 mg, 57.8 µmol) was dissolved in benzene (0.5 mL) and allowed to react at room temperature overnight. All volatiles were removed *in vacuo* to afford 1,4-bis(*para*-tolyl)-2-bromo-3-dimethylamino-1,4-diaza-2,3-diborinine (**8c**) (41.3 mg, 108 µmol, 94%) analytically pure as a colorless powder. Crystals suitable for X-ray diffraction were obtained from saturated benzene solutions at room temperature.

^{1}H-NMR (500 MHz, C_6D_6): δ = 7.09-6.87 (m, 8H, aryl-C*H*), 6.04 (d, 1H, HC=C*H*, $^{3}J_{HH}$ = 6.31 Hz,), 5.77 (d, 1H, *H*C=CH, $^{3}J_{HH}$ = 6.31 Hz,), 2.71 (br s, 6H, N(C*H*$_3$)$_2$), 2.08 (s, 6H, *para*-C*H*$_3$), 2.06 (s, 6H, *para*-C*H*$_3$). **^{11}B{^{1}H}-NMR** (160 MHz, C_6D_6): δ = 40.1 (br. s, *B*Br, $\omega_{1/2}$ = 1198), 32.0 (br, s, *B*N(CH$_3$)$_2$, $\omega_{1/2}$ = 941). **^{13}C{^{1}H}-NMR** (125 MHz, C_6D_6): δ = 147.93 (s, *ipso*-*C*$_q$), 146.52 (s, *ipso*-*C*$_q$), 136.38 (s, *para*-*C*$_q$), 134.48 (s, *para*-*C*$_q$), 130.06 (s, *meta*-*C*H), 129.86 (s, *meta*-*C*H), 127.28 (s, *ortho*-*C*H), 126.06 (s, *ortho*-*C*H), 122.89 (s, HC=*C*H), 115.39 (s, H*C*=CH), 42.16 (br s, N(*C*H$_3$)$_2$), 20.93 (s, *ortho*-C*H*$_3$), 20.84 (s, *ortho*-C*H*$_3$). **El. Anal. [%]**: calculated for $C_{18}H_{22}B_2BrN_3$ (381.92 g/mol): C 56.61, H 5.81, N 11.00; found: C 56.66, H 5.84, N 10.83.

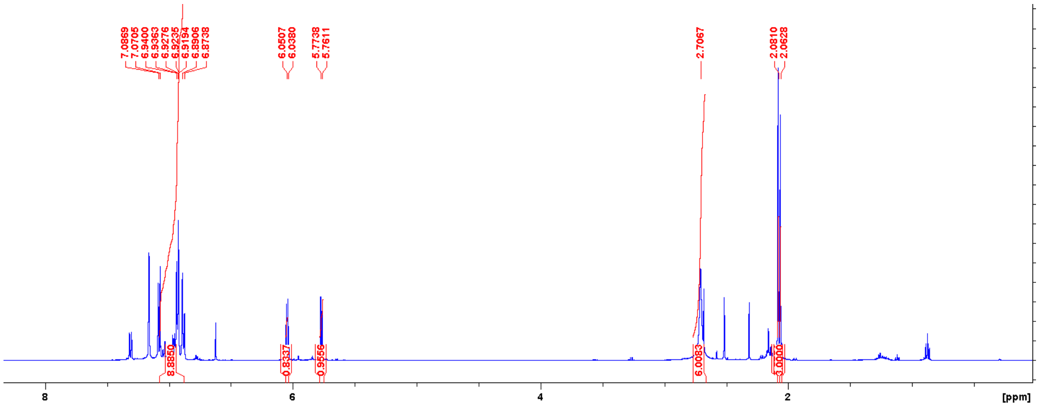

Figure S55. ^{1}H NMR spectrum of **8c** in C_6D_6.

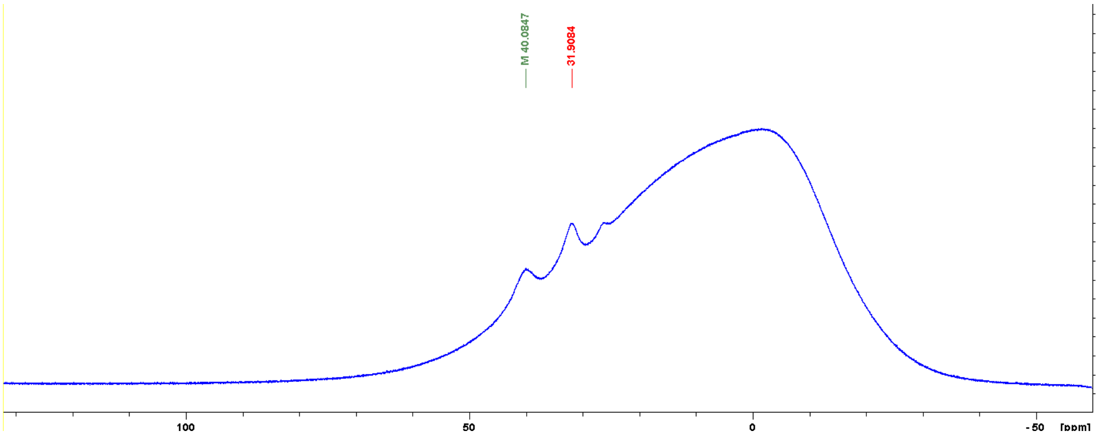

Figure S56. ^{11}B NMR spectrum of **8c** in C_6D_6.

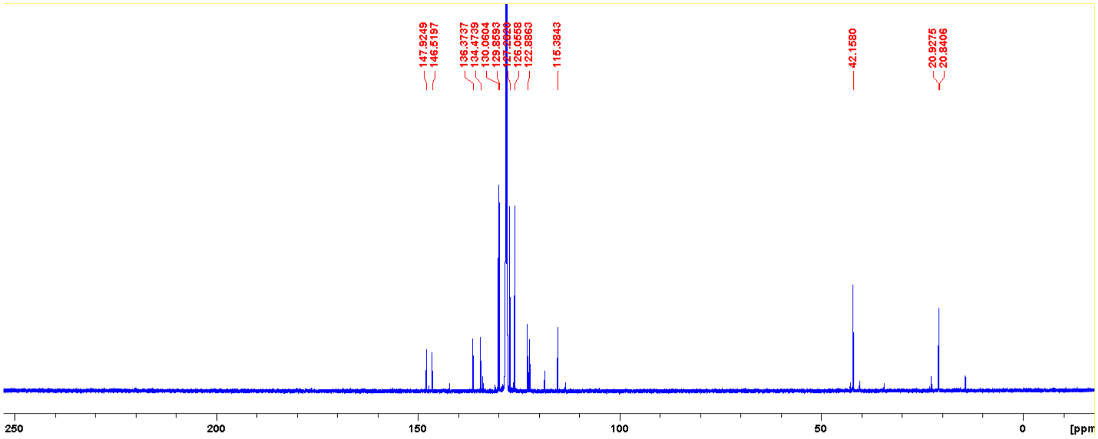

Figure S57. ^{13}C NMR spectrum of **8c** in C_6D_6.

Synthesis and characterization of 9

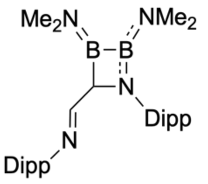

To a solution of *N,N'*-bis(2,6-di*iso*propylphenyl)-1,4-diazabutadiene (1.05 g, 2.79 mmol) in THF (25 mL) was added excess lithium metal, and the solution was stirred at ambient temperature until an intense yellow color has formed. The mixture was cannulated to remove excess lithium, and treated dropwise with a solution of $B_2Cl_2(NMe_2)_2$ (463 µL, 504 mg, 2.79 mmol) in THF (15 mL) at -78 °C. The reaction mixture was allowed to reach at room temperature overnight resulting in the formation of a dark red solution. All volatiles were removed *in vacuo*, and the crude product was extracted into pentane, concentrated and stored at -30 °C overnight to yield colorless crystals. The mother liquor was removed, the crystals washed with cold pentane and dried *in vacuo* to yield **9** (350 mg, 719 µmol, 26%). Crystals suitable for X-ray diffraction were obtained from saturated pentane solutions at -30 °C. Due to slow conversion of **9** into **10**, no clean ^{13}C NMR spectra could be obtained.

^{1}H-NMR (400.13 MHz, C_6D_6): δ = 7.93 (d, 1H, N=C*H*, $^{3}J_{HH}$ = 7.2 Hz), 7.12-6.99 (m, 6H, aryl-C*H*), 4.69 (d, 1H, BC*H*, $^{3}J_{HH}$ = 7.2 Hz), 3.89 (sept, 1H, C*H*(CH$_3$)$_2$, $^{3}J_{HH}$ = 6.88 Hz), 3.54 (sept, 1H, C*H*(CH$_3$)$_2$, $^{3}J_{HH}$ = 6.84 Hz), 2.92 (s, 3H, NC*H*$_3$), 2.77 (s, 3H, NC*H*$_3$), 2.68 (br s, 3H, NC*H*$_3$), 2.27 (br s, 3H, NC*H*$_3$), 1.42 (d, 3H, CH(CH$_3$)(C*H*$_3$), $^{3}J_{HH}$= 6.88 Hz), 1.20 (d, 3H, CH(CH$_3$)(C*H*$_3$), $^{3}J_{HH}$= 6.83 Hz), 1.16 (d, 3H, CH(CH$_3$)(C*H*$_3$), $^{3}J_{HH}$= 6.84 Hz), 1.13 (d, 6H, CH(CH$_3$)(C*H*$_3$), $^{3}J_{HH}$= 6.84 Hz), 1.10 (d, 6H, CH(CH$_3$)(C*H*$_3$), $^{3}J_{HH}$= 6.88 Hz), 0.92 (d, 3H, CH(CH$_3$)(C*H*$_3$), $^{3}J_{HH}$= 6.88 Hz). **^{11}B-NMR** (160 MHz, C_6D_6): δ = 48.7 (br, s, $\omega_{1/2}$ = 1049), 33.1 (br, s, $\omega_{1/2}$ = 864).

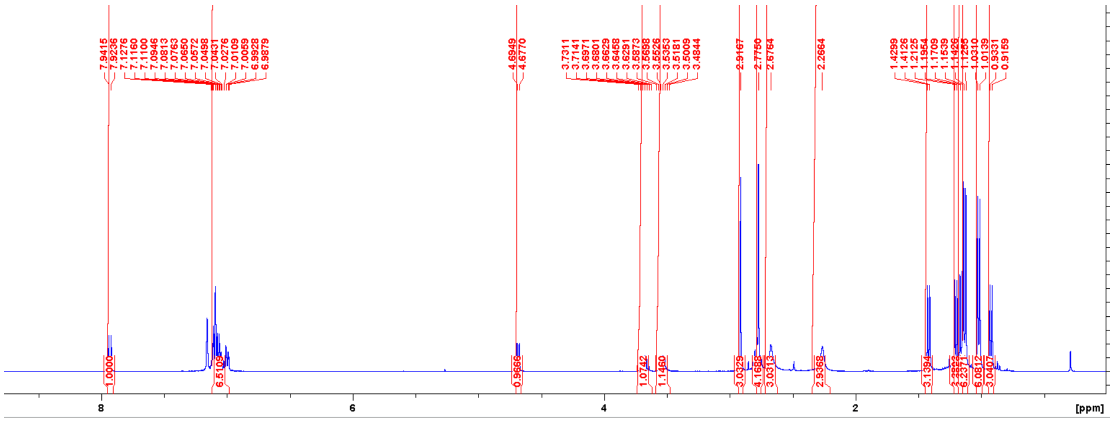

Figure S58. ^{1}H NMR spectrum of **9** in C_6D_6.

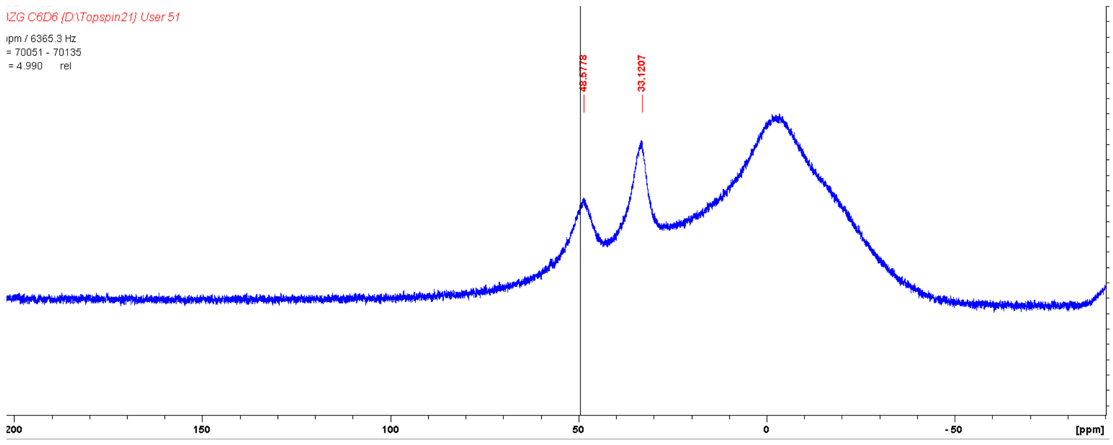

Figure S59. ^{11}B NMR spectrum of **9** in C_6D_6.

Synthesis and characterization of 10

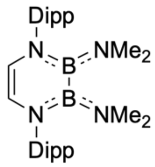

In a J. Young NMR tube, a solution of **9** (8.5 mg, 17.5 µmol) in C_6D_6 (0.5 mL) was heated at 60 °C for 2h. Full conversion into 1,4-bis(2,6-di*iso*propylphenyl)-2,3-bis(dimethylamino)-1,4-diaza-2,3-diborinin (**10**) could be verified by ^1H NMR and ^{11}B NMR spectroscopy. All attempts to crystallize **10** failed.

^{1}H-NMR (400.13 MHz, C_6D_6): δ = 7.22-7.19 (m, 2H, aryl-C*H*), 7.14-7.12 (m, 4H, aryl-C*H*), 5.27 (s, 2H, HC=C*H*), 3.51 (sept, 4H, C*H*(CH_3)$_2$, $^3J_{HH}$ = 6.90 Hz), 2.49 (s, 12H, N(C*H*$_3$)$_2$), 1.24 ppm (t, 24H, CH(C*H*$_3$)$_2$, $^3J_{HH}$ = 6.90 Hz). **^{11}B-NMR** (128.38. MHz, C_6D_6): δ = 33.8 (s, br, $\omega_{1/2}$ = 1090). **El. Anal.** [%]: calculated for $C_{30}H_{48}B_2N_4$ (486.41 g·mol^{-1}): C 74.09, H 9.95, N 11.52; found: C 74.13, H 9.97, N 11.50.

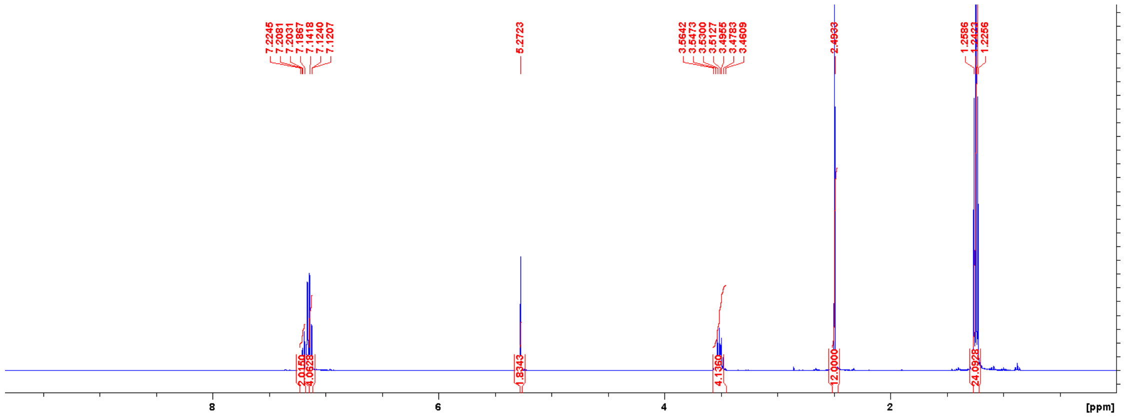

Figure S60. ^1H NMR spectrum of **10** in C_6D_6.

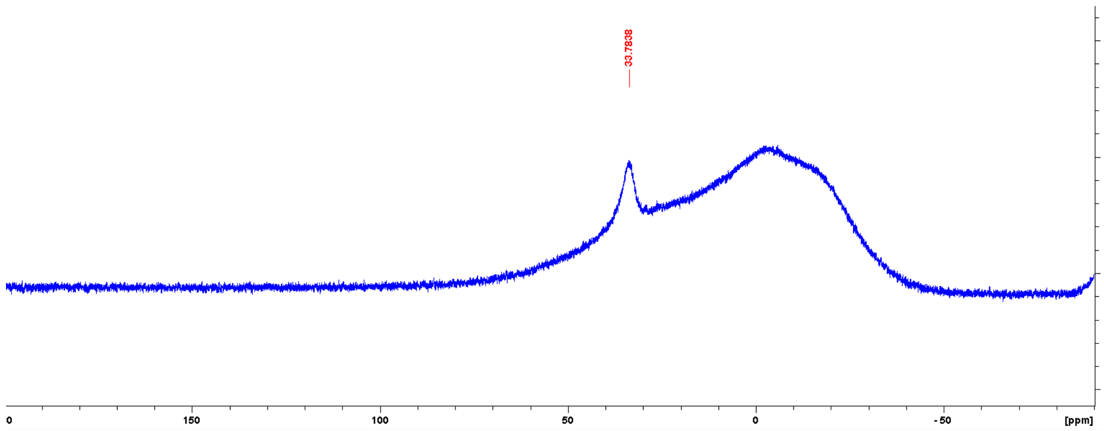

Figure S61. ^{11}B NMR spectrum of **10** in C_6D_6.

S2 X-ray diffraction data

General remarks: The crystal data of **1b**, **1d**, **2a-c**, **3a**, **3c**, **4a**, **5a-b**, and **7c** were collected on a BRUKER D8 QUEST diffractometer with a CMOS area detector and multi-layer mirror monochromated $Mo_{K\alpha}$ radiation. The crystal data of **1c**, **3b**, **7a-b**, **8a-c** and **9** were collected on a BRUKER X8-APEX II diffractometer with a CCD area detector and multi-layer mirror monochromated $Mo_{K\alpha}$ radiation. The structures were solved using intrinsic phasing method (SHELXT),[7] refined with the SHELXL program,[7] and expanded using Fourier techniques. All non-hydrogen atoms were refined anisotropically. Hydrogen atoms were included in structure factors calculations. All hydrogen atoms were assigned to idealized geometric positions.

Crystal data for **1b**: $C_{11}H_{16}BN_2$, M_r = 187.07, colourless block, 0.531×0.146×0.128 mm^3, Triclinic space group $P\bar{1}$, a = 8.141(4) Å, b = 11.715(7) Å, c = 12.636(7) Å, α = 100.38(2)°, β = 102.377(16)°, γ = 102.972(15)°, V = 1113.5(10) Å^3, Z = 4, ρ_{calcd} = 1.116 g·cm^{-3}, μ = 0.065 mm^{-1}, F(000) = 404, T = 100(2) K, R_1 = 0.0795, wR^2 = 0.1645, 4369 independent reflections [2θ≤52.038°] and 261 parameters. CCDC 1967424.

Crystal data for **1c**: $C_{20}H_{28}B_2N_4$, M_r = 346.08, colourless block, 0.386×0.207×0.206 mm^3, Monoclinic space group $C2/c$, a = 18.331(3) Å, b = 13.556(3) Å, c = 7.900(4) Å, β = 101.861(10)°, V = 1921.1(12) Å^3, Z = 4, ρ_{calcd} = 1.197 g·cm^{-3}, μ = 0.071 mm^{-1}, F(000) = 744, T = 100(2) K, R_1 = 0.0465, wR^2 = 0.1131, 1897 independent reflections [2θ≤52.022°] and 121 parameters. CCDC 1967425.

Crystal data for **1d**: $C_{14}H_{32}B_2N_4$, M_r = 278.05, colourless block, 0.301×0.279×0.237 mm^3, Triclinic space group $P\bar{1}$, a = 9.800(3) Å, b = 10.193(3) Å, c = 10.458(5) Å, α = 100.898(18)°, β = 95.77(2)°, γ = 116.06(3)°, V = 901.2(6) Å^3, Z = 2, ρ_{calcd} = 1.025 g·cm^{-3}, μ = 0.060 mm^{-1}, F(000) = 308, T = 102(2) K, R_1 = 0.0386, wR^2 = 0.1014, 3549 independent reflections [2θ≤52.04°] and 191 parameters. CCDC 1967427.

Crystal data for **2a**: $C_{20}H_{24}B_2Cl_2N_2$, M_r = 384.93, colourless plate, 0.267×0.209×0.162 mm^3, Monoclinic space group $P2_1/c$, a = 12.103(5) Å, b = 13.377(10) Å, c = 12.835(5) Å, β = 102.653(9)°, V = 2027.5(19) Å^3, Z = 4, ρ_{calcd} = 1.261 g·cm^{-3}, μ = 0.326 mm^{-1}, F(000) = 808, T = 100(2) K, R_1 = 0.0723, wR^2 = 0.2119, 3985 independent reflections [2θ≤52.044°] and 242 parameters. CCDC 1967428.

Crystal data for **2b**: $C_{18}H_{20}B_2Cl_2N_2$, M_r = 356.88, colourless block, 0.318×0.231×0.154 mm^3, Monoclinic space group $P2_1$, a = 7.1685(3) Å, b = 12.3456(5) Å, c = 10.9958(4) Å, β = 104.8520(10)°, V = 940.61(6) Å^3, Z = 2, ρ_{calcd} = 1.260 g·cm^{-3}, μ = 0.346 mm^{-1}, F(000) = 372, T = 105(2) K, R_1 = 0.0209, wR^2 = 0.0526, 3714 independent reflections [2θ≤52.034°] and 221 parameters. CCDC 1967429.

Crystal data for **2c**: $C_{16}H_{16}B_2Cl_2N_2$, $M_r = 328.83$, colourless block, 0.372×0.167×0.145 mm^3, Orthorhombic space group *Pbca*, $a = 14.960(6)$ Å, $b = 7.423(3)$ Å, $c = 58.652(18)$ Å, $V = 6513(4)$ Å^3, $Z = 16$, $\rho_{calcd} = 1.341$ g·cm^{-3}, $\mu = 0.394$ mm^{-1}, $F(000) = 2720$, $T = 100(2)$ K, $R_1 = 0.0362$, $wR^2 = 0.0788$, 6399 independent reflections [2θ≤52.044°] and 401 parameters. CCDC 1967431.

Crystal data for **3a**: $C_{20}H_{24}B_2Br_2N_2$, $M_r = 473.85$, colourless block, 0.292×0.215×0.064 mm^3, Monoclinic space group $P2_1/c$, $a = 12.3052(13)$ Å, $b = 13.7337(17)$ Å, $c = 12.7722(16)$ Å, $\beta = 102.865(4)°$, $V = 2104.3(4)$ Å^3, $Z = 4$, $\rho_{calcd} = 1.496$ g·cm^{-3}, $\mu = 3.858$ mm^{-1}, $F(000) = 952$, $T = 100(2)$ K, $R_1 = 0.0914$, $wR^2 = 0.1589$, 4140 independent reflections [2θ≤52.044°] and 241 parameters. CCDC 1967434.

Crystal data for **3b**: $C_{18}H_{20}B_2Br_2N_2$, $M_r = 445.80$, colourless block, 0.435×0.172×0.086 mm^3, Monoclinic space group $P2_1/c$, $a = 11.404(2)$ Å, $b = 13.260(3)$ Å, $c = 13.309(4)$ Å, $\beta = 111.269(7)°$, $V = 1875.5(8)$ Å^3, $Z = 4$, $\rho_{calcd} = 1.579$ g·cm^{-3}, $\mu = 4.324$ mm^{-1}, $F(000) = 888$, $T = 103(2)$ K, $R_1 = 0.0629$, $wR^2 = 0.1315$, 3695 independent reflections [2θ≤52.044°] and 222 parameters. The crystal was a pseudo-merohedral twin with domains rotated by 179.5 ° around real axis [-0.002 0.001 1.000]. The BASF parameter was refined to 0%. CCDC 1967435.

Crystal data for **3c**: $C_{16}H_{16}B_2Br_2N_2$, $M_r = 417.75$, colourless plate, 0.334×0.303×0.124 mm^3, Monoclinic space group $C2/c$, $a = 5.933(2)$ Å, $b = 14.356(4)$ Å, $c = 20.101(7)$ Å, $\beta = 94.007(11)°$, $V = 1707.8(10)$ Å^3, $Z = 4$, $\rho_{calcd} = 1.625$ g·cm^{-3}, $\mu = 4.742$ mm^{-1}, $F(000) = 824$, $T = 100(2)$ K, $R_1 = 0.0259$, $wR^2 = 0.0619$, 1677 independent reflections [2θ≤52.016°] and 101 parameters. CCDC 1967436.

Crystal data for **4a**: $C_{20}H_{24}B_2I_2N_2$, $M_r = 567.85$, colourless block, 0.338x0.401x0.489 mm^3, Orthorhombic space group $Pna2_1$, $a = 24.4526(8)$ Å, $b = 7.5671(3)$ Å, $c = 12.1757(4)$ Å, $V = 2252.93(14)$ Å^3, $Z = 4$, $\rho_{calcd} = 1.674$ g·cm^{-3}, $F(000) = 1096$, $T = 100(2)$ K, $R_1 = 0.0199$, $wR^2 = 0.0500$, 4138 independent reflections [2θ≤54.20°] and 241 parameters. CCDC 1967437.

Crystal data for **5a**: $C_{22}H_{30}B_2N_2$, $M_r = 344.10$, colourless block, 0.322×0.192×0.14 mm^3, Monoclinic space group $P2_1/c$, $a = 12.189(5)$ Å, $b = 13.537(5)$ Å, $c = 12.813(3)$ Å, $\beta = 102.69(3)°$, $V = 2062.5(13)$ Å^3, $Z = 4$, $\rho_{calcd} = 1.108$ g·cm^{-3}, $\mu = 0.063$ mm^{-1}, $F(000) = 744$, $T = 100(2)$ K, $R_1 = 0.1217$, $wR^2 = 0.2296$, 3305 independent reflections [2θ≤48.37°] and 244 parameters. CCDC 1967438.

Crystal data for **5b**: $C_{20}H_{26}B_2N_2$, M_r = 316.05, colourless plate, 0.325×0.25×0.072 mm^3, Monoclinic space group $P2_1/n$, a = 8.151(4) Å, b = 21.098(8) Å, c = 11.361(5) Å, β = 101.828(18)°, V = 1912.3(15) Å^3, Z = 4, ρ_{calcd} = 1.098 g·cm^{-3}, μ = 0.062 mm^{-1}, F(000) = 680, T = 102(2) K, R_1 = 0.0664, wR^2 = 0.1136, 3750 independent reflections [2θ≤52.03°] and 223 parameters. CCDC 1967439.

Crystal data for **7a**: $C_{22}H_{30}B_2ClN_3$, M_r = 393.56, colourless plate, 0.529×0.377×0.30 mm^3, Monoclinic space group $P2_1/c$, a = 11.724(6) Å, b = 7.743(3) Å, c = 24.880(7) Å, β = 101.79(2)°, V = 2210.9(16) Å^3, Z = 4, ρ_{calcd} = 1.182 g·cm^{-3}, μ = 0.185 mm^{-1}, F(000) = 840, T = 104(2) K, R_1 = 0.0431, wR^2 = 0.1108, 4361 independent reflections [2θ≤52.04°] and 261 parameters. CCDC 1967585.

Crystal data for **7b**: $C_{22}H_{30}B_2ClN_3$, M_r = 365.51, colourless plate, 0.529×0.377×0.30 mm^3, Monoclinic space group P 1 $2_1/n$ 1, a = 11.8363(14) Å, b = 7.6690(11) Å, c = 22.104(3) Å, β = 97.063(5)°, V = 1991.2(5) Å^3, Z = 4, ρ_{calcd} = 1.219 g·cm^{-3}, F(000) = 776, T = 100(2) K, R_1 = 0.1133, wR^2 = 0.1964, 4352 independent reflections [2θ≤54.08°] and 241 parameters. CCDC 1967446.

Crystal data for **7c**: $C_{18}H_{22}B_2ClN_3$, M_r = 337.45, colourless block, 0.216×0.122×0.116 mm^3, Monoclinic space group $P2_1$, a = 5.9624(17) Å, b = 7.859(3) Å, c = 19.044(9) Å, β = 92.007(15)°, V = 891.8(6) Å^3, Z = 2, ρ_{calcd} = 1.257 g·cm^{-3}, μ = 0.218 mm^{-1}, F(000) = 356, T = 100(2) K, R_1 = 0.0432, wR^2 = 0.1114, 2638 independent reflections [2θ≤52.036°] and 221 parameters. CCDC 1967447.

Crystal data for **8a**: $C_{22}H_{30}B_2BrN_3$, M_r = 438.02, colourless block, 0.157×0.093×0.042 mm^3, Monoclinic space group $P2_1/c$, a = 11.860(5) Å, b = 7.788(3) Å, c = 24.705(10) Å, β = 100.325(19)°, V = 2244.9(16) Å^3, Z = 4, ρ_{calcd} = 1.296 g·cm^{-3}, μ = 1.843 mm^{-1}, F(000) = 912, T = 104(2) K, R_1 = 0.0483, wR^2 = 0.0889, 4425 independent reflections [2θ≤52.044°] and 261 parameters. CCDC 1967441.

Crystal data for **8b**: $C_{20}H_{26}B_2BrN_3$, M_r = 409.97, colourless block, 0.368×0.261×0.104 mm^3, Monoclinic space group $P2_1/n$, a = 11.810(6) Å, b = 7.778(4) Å, c = 22.194(9) Å, β = 97.26(3)°, V = 2022.4(16) Å^3, Z = 4, ρ_{calcd} = 1.346 g·cm^{-3}, μ = 2.041 mm^{-1}, F(000) = 848, T = 100(2) K, R_1 = 0.0401, wR^2 = 0.0778, 3995 independent reflections [2θ≤52.042°] and 241 parameters. CCDC 1967448.

Crystal data for **8c**: $C_{18}H_{22}B_2BrN_3$, $M_r = 381.91$, colourless plate, 0.38×0.267×0.098 mm^3, Monoclinic space group $P2_1$, $a = 5.9047(12)$ Å, $b = 7.916(4)$ Å, $c = 19.300(3)$ Å, $\beta = 91.872(10)°$, $V = 901.6(5)$ Å^3, $Z = 2$, $\rho_{calcd} = 1.407$ g·cm^{-3}, $\mu = 2.283$ mm^{-1}, $F(000) = 392$, $T = 100(2)$ K, $R_1 = 0.0525$, $wR^2 = 0.0752$, 3404 independent reflections [2θ≤52.028°] and 221 parameters. CCDC 1967449.

Crystal data for **9**: $C_{30}H_{48}B_2N_4$, $M_r = 486.34$, colourless block, 0.565×0.152×0.138 mm^3, Monoclinic space group $P2_1/n$, $a = 11.161(8)$ Å, $b = 14.999(9)$ Å, $c = 18.338(11)$ Å, $\beta = 91.160(12)°$, $V = 3069(3)$ Å^3, $Z = 4$, $\rho_{calcd} = 1.052$ g·cm^{-3}, $\mu = 0.061$ mm^{-1}, $F(000) = 1064$, $T = 104(2)$ K, $R_1 = 0.0891$, $wR^2 = 0.1229$, 6056 independent reflections [2θ≤52.044°] and 366 parameters. CCDC 1967450.

Crystallographic data have been deposited with the Cambridge Crystallographic Data Centre supplementary publication nos. CCDC 1967424-1967425, 1967427-1967429, 1967431, 1967434-1967439, 1967441, 1967446-1967450, and 1967585. These data can be obtained free of charge from The Cambridge Crystallographic Data Centre *via* www.ccdc.cam.ac.uk/data_request/cif.

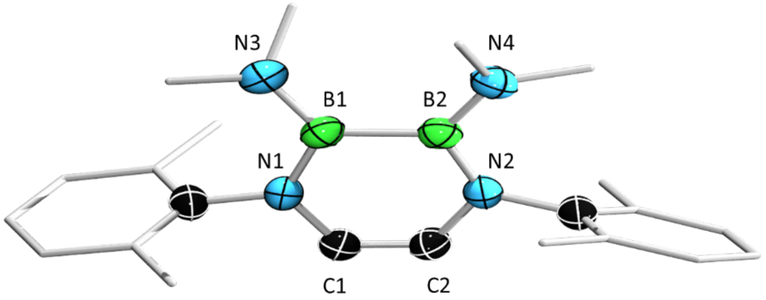

Figure S62. X-ray crystallographic structure of compound **1b**. Ellipsoids represented at the 50% probability level. The ellipsoids of the hydrogen and part of the carbon atoms are omitted for clarity. Selected bond lengths (Å), angles and torsion angles (º): B2-B1 1.719(3), B1-N1 1.441(3), N1-C1 1.399(3), C1-C2 1.318(3), C2-N2 1.405(3), N2-B2 1.441(3), B2-N4 1.430(3), B1-N3 1.449(3), N2-B2-B1 114.6(2), B2-B1-N1 114.1(2), B1-N1-C1 121.0(2), N1-C1-C2 124.7(2), C1-C2-N2 124.4(2), C2-N2-B2 120.8(2), N4-B2-B1 124.9(2), B2-B1-N3 125.5(2), N3-B1-N1 120.3(2), N2-B2-N4 120.5(2), N2-C2-C1-N1 2.2(3), C2-C1-N1-B1 2.8(3), C1-N1-B1-B2 -5.9(3), N1-B1-B2-N2 5.0(3), B1-B2-N2-C2 -0.9(3), B2-N2-C2-C1 -2.8(3), B2-N2-C2-C1 -2.8(3), B2-N2-C2-C1 -2.8(3), C2-N2-C11-C12 78.7(2), C1-N1-C3-C8 -93.5(2), N1-B1-N3-C19 25.6(3), N2-B2-N4-C21 -148.0(2).

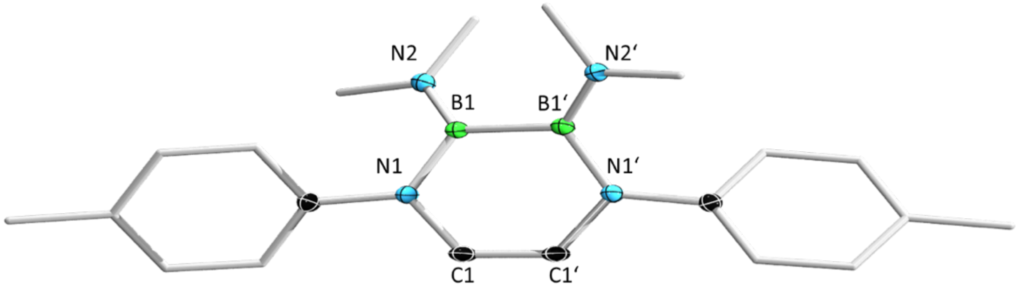

Figure S63. X-ray crystallographic structure of compound **1c**. Ellipsoids represented at 50% probability. The ellipsoids of the hydrogen and part of the carbon atoms are omitted for clarity. Selected bond lengths (Å), angles and torsion angles (º): B1-B1’ 1.708(2), B1-N1 1.472(2), N1-C1 1.411(2), C1-C1’ 1.338(2), B1-N2 1.409(2), B1’-B1-N1 111.3(1), B1’-B1-N2 126.6(1), N2-B1-N1 121.7(1), B1-N1-C1 116.0(1), N1-C1-C1’ 125.2(1), B1’-B1-N1-C1 -32.4(2), B1-N1-C1-C1’ 8.5(2), N1-C1-C1’-N1’ 6.7(2), C1’-C1-N1-B1 8.5(2), C1-N1-B1-B1’ -32.4(2), N1-B1-B1’-N1’ 45.0(2), C1-N1-C2-C3 -33.5(2), N1-B1-N2-C9 -3.5(2).

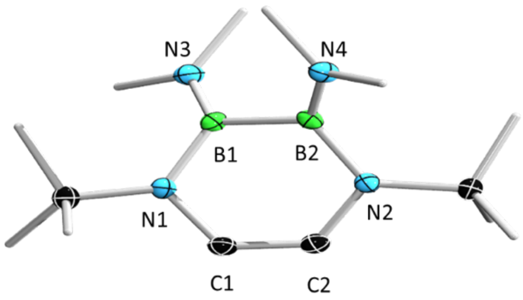

Figure S64. X-ray crystallographic structure of compound **1d**. Ellipsoids represented at 50% probability level. The ellipsoids of the hydrogen and part of the carbon atoms are omitted for clarity. Selected bond lengths (Å), angles and torsion angles (°): B1-B2 1.704(2), B2-N2 1.475(2), N2-C2 1.409(1), C2-C1 1.341(2), C1-N1 1.409(2), N1-B1 1.478(2), B1-N3 1.406(2), B2-N4 1.412(2), N1-B1-B2 109.80(9), B2-B1-N3 127.69(9), N3-B1-N1 121.90(9), B1-N1-C1 113.29(8), N1-C1-C2 125.6(1), C1-C2-N2 125.76(9), C2-N2-B2 114.21(8), N2-B2-B1 110.96(9), N2-B2-N4 123.20(9), N4-B2-B1 124.54(9), B1-N1-C1-C2 13.0(1), N1-C1-C2-N2 8.8(2), C1-C2-N2-B2 4.5(1), C2-N2-B2-B1 -33.0(1), N2-B2-N1-C1 10.66(8), B2-B1-N1-C1 -40.3(1), B1-N1-C1-C2 13.0(1), C2-N2-B2-N4 134.5(1), C1-N1-B1-N3 131.5(1), N1-B1-B2-N2 52.0(1).

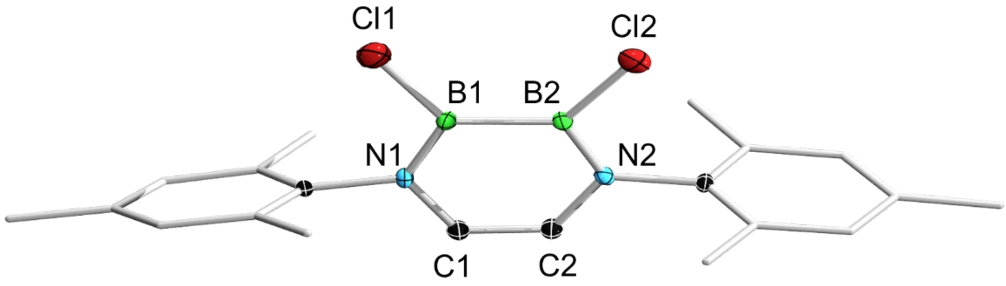

Figure S65. X-ray crystallographic structure of compound **2a**. Ellipsoids represented at 50% probability level and partially omitted for clarity. The ellipsoids of the hydrogen and part of the carbon atoms are omitted for clarity. Selected bond lengths (Å), angles and torsion angles (°): B1-B2 1.667(5), B2-N1 1.411(4), N1-C1 1.401(4), C1-C2 1.347(4), C2-N2 1.400(4), N2-B1 1.402(5), B1-Cl2 1.776(4), B2-Cl1 1.768(3), N2-B1-B2 115.6(3), B1-B2-N1 115.4(3), B2-N1-C1 121.2(2), N1-C1-C2 123.1(2), C1-C2-N2 123.2(3), C2-N2-B1 121.3(3), N2-B1-Cl2 118.7(3), Cl2-B1-B2 125.7(3), B1-B2-Cl1 126.3(2), Cl1-B2-N1 118.3(2), B1-B2-N1-C1 -1.1(4), B2-N1-C1-C2 -1.4(4), N1-C1-C2-N2 1.1(4), C1-C2-N2-B1 2.1(4), C2-N2-B1-B2 -4.3(4), N2-B1-B2-N1 3.8(4), C1-N1-C3-C4 99.8(3), C2-N2-C12-C17 -84.6(3).

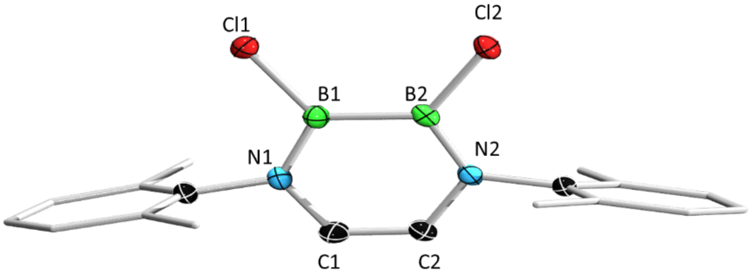

Figure S66. X-ray crystallographic structure of compound **2b**. Ellipsoids represented at 50% probability level. The ellipsoids of the hydrogen and part of the carbon atoms are omitted for clarity. Selected bond lengths (Å), angles and torsion angles (°): B1-B2 1.657(3), B2-N2 1.404(3), N2-C2 1.399(2), C2-C1 1.340(3), C1-N1 1.404(3), N1-B1 1.409(3), B1-Cl1 1.784(2), B2-Cl2 1.789(2), N1-B1-B2 115.9(2), B1-B2-N2 116.1(2), B2-N2-C2 120.3(2), N2-C2-C1 123.6(2), C2-C1-N1 123.6(2), C1-N1-B1 120.2(2), B2-B1-Cl1 125.1(1), Cl1-B1-N1 118.9(1), B1-B2-Cl2 125.1(1), Cl2-B2-N2 118.8(1), N1-B1-B2-N2 5.2(3), B1-B2-N2-C2 -6.9(3), B2-N2-C2-C1 3.7(3), N2-C2-C1-N1 2.1(3), C2-C1-N1-B1 -3.7(3), C1-N1-B1-B2 -0.0(3), C2-N2-C11-C16 -88.1(2), C1-N1-C3-C4 80.2(2).

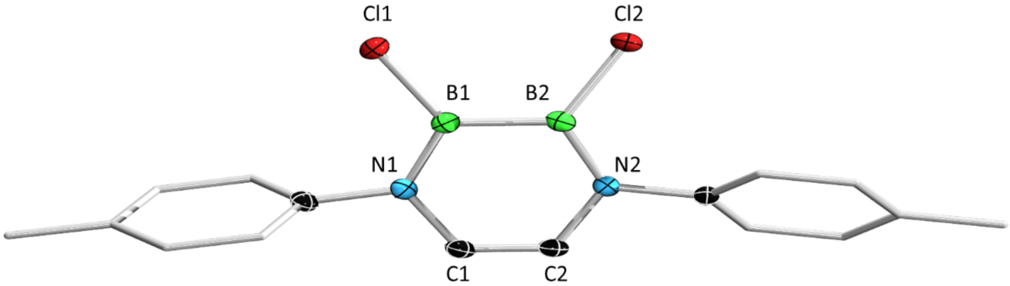

Figure S67. X-ray crystallographic structure of compound **2c**. Ellipsoids represented at 50% probability level. Two molecules present in the asymmetric unit. Due to their similarity, one is omitted for clarity. The ellipsoids of the hydrogen and part of the carbon atoms are omitted for clarity. Selected bond lengths (Å), angles and torsion angles (°): B1-B2 1.653(3), B2-N2 1.409(2), N2-C2 1.405(2), C2-C1 1.337(2), C1-N1 1.402(2), N1-B1 1.413(3), B1-Cl1 1.775(2), Cl2-B2 1.786(2), Cl1-B1 B2 124.4(1), B1-B2-Cl2 122.9(1), Cl2-B2-N2 120.2(1), B1-B2-N2 116.8(1), B2-N2-C2 119.6(1), N2-C2-C1 123.6(1), C2-C1-N1 124.3(1), C1-N1-B1 119.8(1), N1-B1-B2 115.8(1), N1-B1-Cl1 119.8(1), N1-C1-C2-N2 -0.4(3), C1-C2-N2-B2 -0.2(2), C2-N2-B2-B1 1.8(2), N2-B2-B1-N1 -3.0(2), B2-B1-N1-C1 2.6(2), B1-N1-C1-C2 -1.1(2), N1-C1-C2-N2 -0.4(3), C2-N2-C10-C15 -121.5(2), C1-N1-C3-C4 68.0(2), C1-N1-B1-Cl1 -176.1(1), C2-N2-B2-Cl2 -175.4(1).

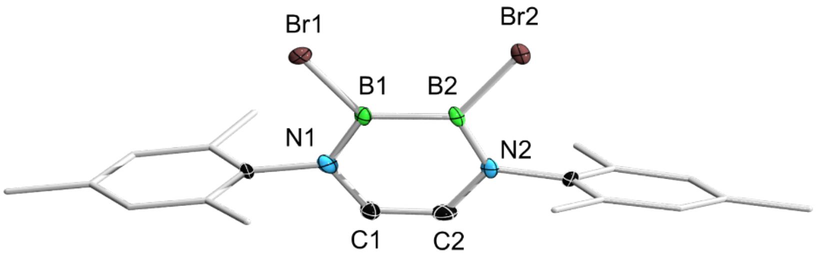

Figure S68. X-ray crystallographic structure of compound **3a**. Ellipsoids represented at 50% probability level. The ellipsoids of the hydrogen and part of the carbon atoms are omitted for clarity. Selected bond lengths (Å), angles and torsion angles (°): B2-B1 1.659(9), B1-N1 1.41(1), N1-C1 1.403(9), C1-C2 1.323(9), C2-N2 1.406(9), N2-B2 1.41(1), B2-Br2 1.933(8), B1-Br1 1.939(8), B2-B1-N1 116.4(6), B1-N1-C1 120.6(6), N1-C1-C2 123.1(7), C1-C2-N2 124.3(7), C2-N2-B2 120.5(6), N2-B2-B1 114.8(6), N2-B2-Br2 119.6(5), Br2-B2-B1 125.5(5), B2-B1-Br1 125.4(5), Br1-B1-N1 118.2(5) ,C2-C1-N1 B1 2(1), C1-N1-B1 B2 2.5(9) ,N1-B1-B2-N2 6.0(9), B1-B2-N2-C2 5.9(9), B2-N2-C2-C1 -2(1),N2-C2-C1-N1 -2(1), C2-C1-N1-B1 2(1), C2-N2-C12-C17 -93.8(7), C1-N1-C3-C4 82.0(8).

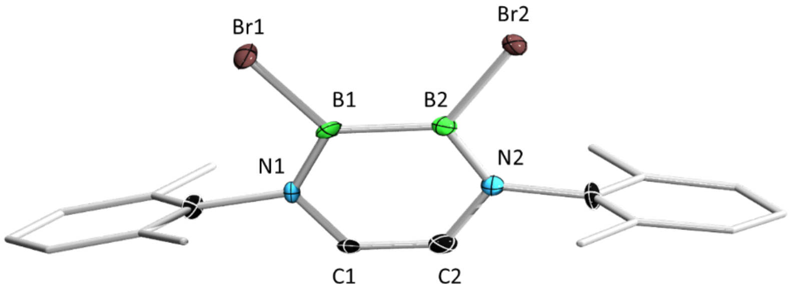

Figure S69. X-ray crystallographic structure of compound **3b**. Ellipsoids represented at 50% probability level. The ellipsoids of the hydrogen and part of the carbon atoms are omitted for clarity. Selected bond lengths (Å), angles and torsion angles (°): B2-B1 1.662(8), B1-N1 1.398(8), N1-C1 1.375(6), C1-C2 1.344(7), C2-N2 1.402(7), N2-B2 1.403(8), B2-Br2 1.942(6), B1-Br1 1.941(6), B2-B1-N1 115.8(5), B1-N1-C1 120.6(5), N1-C1-C2 124.2(5), C1-C2-N2 123.5(5), C2-N2-B2 119.7(5), N2-B2-B1 116.2(5), N2-B2-Br2 119.1(5), Br2-B2-B1 124.7(4), B2-B1-Br1 124.7(4), Br1-B1-N1 119.5(4), N2-C2-C1-N1 1.9(9), C2-C1-N1-B1 -2.8(9), C1-N1-B1-B2 2.6(8), N1-B1-B2-N2 -1.8(8), B1-B2-N2-C2 1.0(8), B2-N2-C2-C1 -1.1(9), B1-B2-N2-C2 1.0(8), C2-N2-C11-C12 90.8(6), C1-N1-C3-C8 -84.5(6).

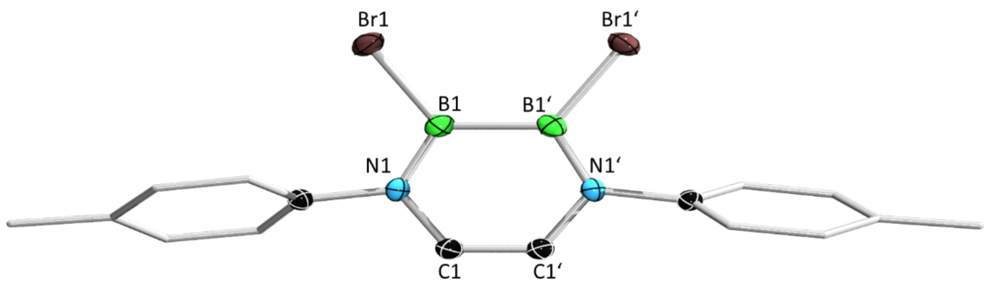

Figure S70. X-ray crystallographic structure of compound **3c**. Ellipsoids represented at 50% probability level. The ellipsoids of the hydrogen and part of the carbon atoms are omitted for clarity. Selected bond lengths (Å), angles and torsion angles (°): B1-B1' 1.665(3), B1-Br1 1.940(2), B1-N1 1.406(3), N1-C1 1.403(3), C1-C1' 1.347(2), B1'-B1-Br1 123.5(2), B1'-B1-N1 116.0(2), Br1-B1-N1 120.5(2), B1-N1-C1-120.4(2), N1-C1-C1'-123.5(2), N1-C1-C1'-N1' 0.3(3), C1'-C1-N1-B1 0.6(3), C1-N1-B1-B1' -1.9(3), N1-B1-B1'-N1' 2.6(3), C1-N1-C2-C7 -67.8(2).

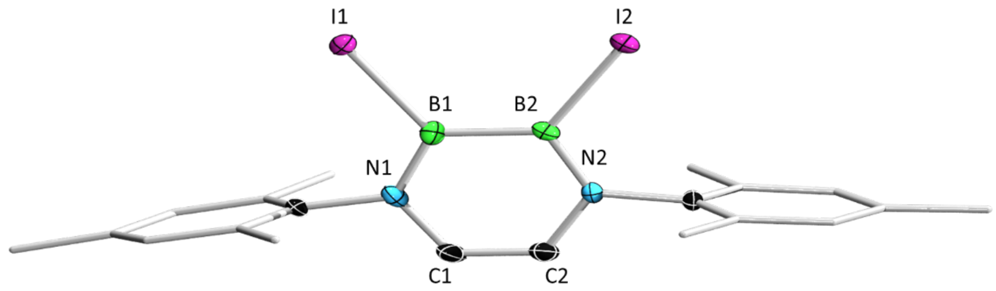

Figure S71. X-ray crystallographic structure of compound **4a**. Ellipsoids represented at 50% probability level. The ellipsoids of the hydrogen and part of the carbon atoms are omitted for clarity. Selected bond lengths (Å), angles and torsion angles (°): B1-B2 1.659(6), B2-N2 1.402(6), N2-C2 1.404(6), C2-C1 1.352(6), C1-N1 1.391(6), N1-B1 1.404(6), B1-I1 2.170(5), I2-B2 2.154(5), B1-B2-N2 115.4(4), B1-B2-I2 125.8(3), I2-B2-N2 118.8(3), B2-N2-C2 121.1(4), N2-C2-C1 122.8(4), C2-C1-N1 123.8(4), C1-N1-B1 120.4(4), N1-B1-B2 116.5(4), B2-B1-I1 124.0(3), I1-B1-N1 119.5(3), B1-B2-N2-C2 -2.3(6), B2-N2-C2-C1 0.9(7), B2-N2-C2-C1 0.9(7), N2-C2-C1-N1 1.0(7), C2-C1-N1-B1 -1.1(7), C1-N1-B1-B2 -0.5(6), N1-B1-B2-N2 2.2(6), B1-B2-N2-C2 -2.3(6), C2-N2-C3-C4 86.6(5), C1-N1-C12-C13 93.1(5).

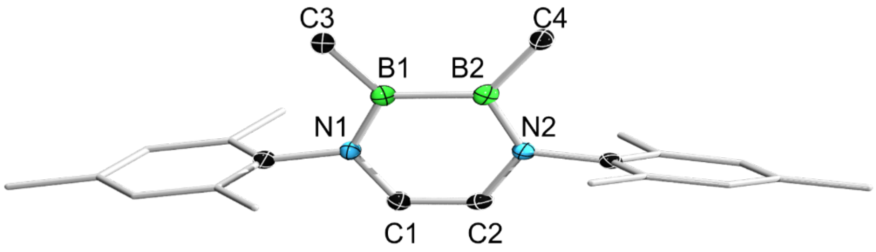

Figure S72. X-ray crystallographic structure of compound **5a**. Ellipsoids represented at 50% probability level. The ellipsoids of the hydrogen and part of the carbon atoms are omitted for clarity. Selected bond lengths (Å), angles and torsion angles (°): B2-B1 1.688(5), B2-N2 1.426(5), N2-C2 1.392(4), C2-C1 1.335(4), C1-N1 1.398(4), N1-B1 1.424(5), B1-C3 1.574(5), B2-C4 1.575(5), B1-N1-C1 122.2(3), N1-C1-C2 123.3(3), C1-C2-N2 122.9(3), C2-N2-B2 122.8(3), N2-B2-B1 114.1(3), B2-B1-N1 114.4(3), N1-B1-C3 118.6(3), C3-B1-B2 127.0(3), B1-B2-C4 127.3(3), C4-B2-N2 118.6(3), N1-C1-C2-N2 -1.5(5), C1-C2-N2-B2 0.1(5), C2-N2-B2-B1 2.9(4), N2-B2-B1-N1 -4.7(4), B2-B1-N1-C1 3.9(4), B1-N1-C1-C2 -0.9(5), N1-C1-C2-N2 -1.5(5), C2-N2-C14-C15 83.2(3), C1-N1-C5-C6 85.0(3).

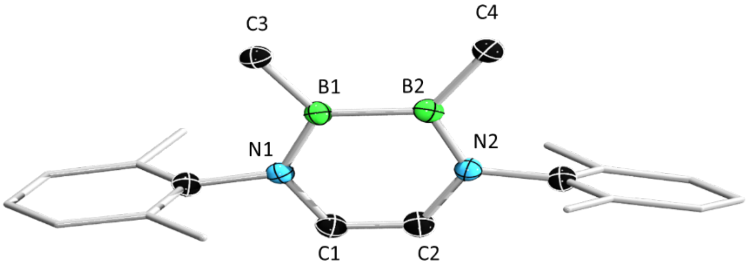

Figure S73. X-ray crystallographic structure of compound **5b**. Ellipsoids represented at 50% probability level. The ellipsoids of the hydrogen and part of the carbon atoms are omitted for clarity. Selected bond lengths (Å), angles and torsion angles (°): B1-B2 1.684(3), B2-N2 1.422(2), N2-C2 1.400(2), C2-C1 1.331(3), C1-N1 1.400(2), N1-B1 1.424(2), B1-C3 1.580(2), B2-C4 1.579(3), B1-B2-N2 114.8(1), B2-N2-C2 121.7(1), N2-C2-C1 123.7(2), C2-C1-N1 123.1(2), C1-N1-B1 122.1(1), N1-B1-B2 114.6(1), C4-B2-B1 125.9(1), C4-B2-N2 119.3(1), B2-B1-C3 126.6(1), C3-B1-N1 118.8(1), N2-C2-C1-N1 -0.7(3), C2-C1-N1-B1 -0.7(2), C1-N1-B1-B2 2.1(2), N1-B1-B2-N2 -2.5(2), B1-B2-N2-C2 1.5(2), B2-N2-C2-C1 0.1(2), N2-C2-C1-N1 -0.7(3), C2-N2-C13-C18 -95.7(2), C1-N1-C5-C6 85.1(2), C1-N1-B1-C3 -177.2(1), C2-N2-B2-C4 -178.9(1).

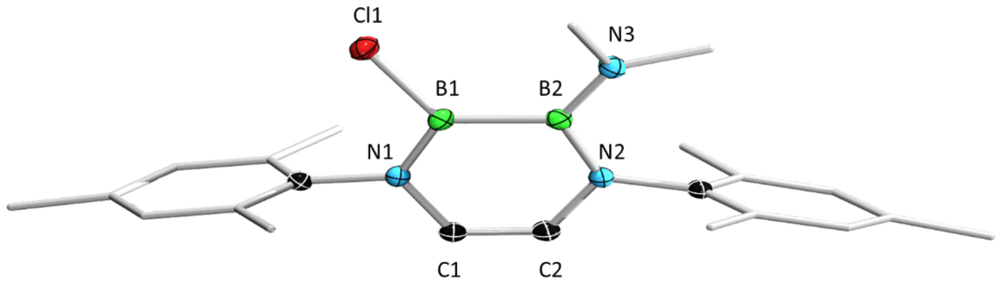

Figure S74. X-ray crystallographic structure of compound **7a**. Ellipsoids represented at 50% probability level. The ellipsoids of the hydrogen and part of the carbon atoms are omitted for clarity. Selected bond lengths (Å), angles and torsion angles (°): B2-B1 1.699(2), B1-N1 1.405(2), N1-C1 1.415(2), C1-C2 1.337(2), C2-N2 1.400(2), N2-B2 1.457(2), N3-B2 1.419(2), B1-Cl1 1.803(2), B2-B1-N1 118.6(1), B1-N1-C1 119.6(1), N1-C1-C2 122.9(1), C1-C2-N2 125.5(1), C2-N2-B2 120.8(1), N2-B2-B1 111.7(1), N2-B2-N3 123.0(1), N3-B2-B1 125.1(1), B2-B1-Cl1 126.0(1), Cl1-B1-N1 114.9(1), B2-N3-C21 121.1(1), B2-N3-C22 128.2(1), Cl1-B1-N1 114.9(1), N2-B2-B1-N1 6.6(2), B2-B1-N1-C1 1.4(2), B2-B1-N1-C1 1.4(2), B2-B1-N1-C1 1.4(2), B1-N1-C1-C2 -6.4(2), N1-C1-C2-N2 2.6(2), C1-C2-N2-B2 6.8(2), C2-N2-B2-B1 -10.4(2), C2-N2-B2-B1 -10.4(2), N2-B2-B1-N1 6.6(2), C1-N1-C3-C8 -81.1(2), C2-N2-C12-C13 77.6(2), N2-B2-N3-C22 18.6(2).

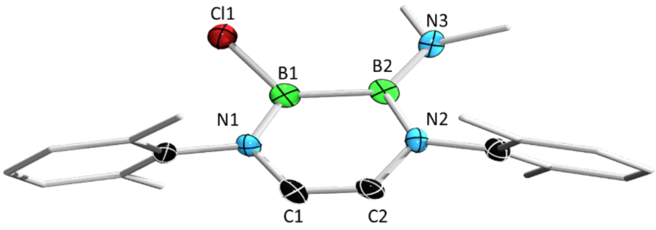

Figure S75. X-ray crystallographic structure of compound **7b**. Ellipsoids represented at 50% probability level and partially omitted for clarity. The ellipsoids of the hydrogen and part of the carbon atoms are omitted for clarity. Selected bond lengths (Å), angles and torsion angles (°): B1-B2 1.698(8), B2-N2 1.454(7), N2-C2 1.405(6), C2-C1 1.329(6), C1-N1 1.412(6), N1-B1 1.396(7), B1-Cl1 1.794(6), N3-B2 1.451(7), Cl1-B1-B2 125.2(4), Cl1-B1-N1 116.2(4), N1-B1-B2 117.9(5), B1-B2-N2 112.2(4), B2-N2-C2 120.7(4), B2-N2-C2 120.7(4), N2-C2-C1 124.6(4), C2-C1-N1 123.6(4), C1-N1-B1 119.9(4), B1-B2-N3 125.6(4), N3-B2-N2 122.0(4), B1-B2-N3-C17 -27.6(7), B1-B2-N2-C2 10.8(6), B2-N2-C2-C1 -8.3(7), N2-C2-C1-N1 -1.4(8), C2-C1-N1-B1 6.9(7), C1-N1-B1-B2 -2.8(7), N1-B1-B2-N2 -5.6(7), C1-N1-C4-C3 80.4(5), C2-N2-C7-C11 -80.6(5).

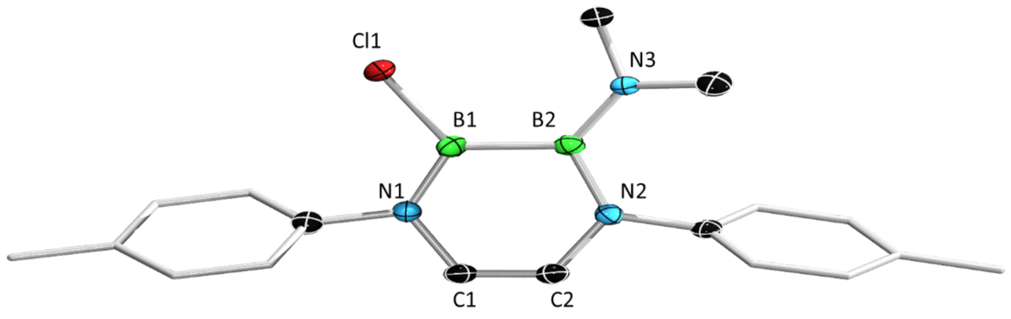

Figure S76. X-ray crystallographic structure of compound **7c**. Ellipsoids represented at 50% probability level. The ellipsoids of the hydrogen and part of the carbon atoms are omitted for clarity. Selected bond lengths (Å), angles and torsion angles (°): B2-B1 1.696(5), B1-N1 1.408(5), N1-C1 1.427(5), C1-C2 1.326(5), C1-C2 1.326(5), C2-N2 1.402(4), N2-B2 1.454(5), B2-N3 1.422(5), B1-Cl1 1.803(4), B2-B1-N1 118.2(3), B2-B1-Cl1 124.1(2), Cl1-B1-N1 117.2(3), B1-B2-N3 126.1(3), N3-B2-N2 121.4(3), B1-B2-N2 112.3(3), B2-N2-C2 121.2(3), N2-C2-C1 125.5(3), C2-C1-N1 122.8(3), C1-N1-B1 119.9(3), N2-C2-C1-N1 -0.4(6), C2-C1-N1-B1 0.8(5), C1-N1-B1-B2 -1.8(5), N1-B1-B2-N2 2.5(4), B1-B2-N2-C2 -2.2(4), B2-N2-C2-C1 1.4(5), C1-N1-C3-C4 52.5(4), C2-N2-C10-C15 -67.0(4), B1-B2-N3-C18 148.5(3), C1-N1-B1-Cl1 -173.7(2).

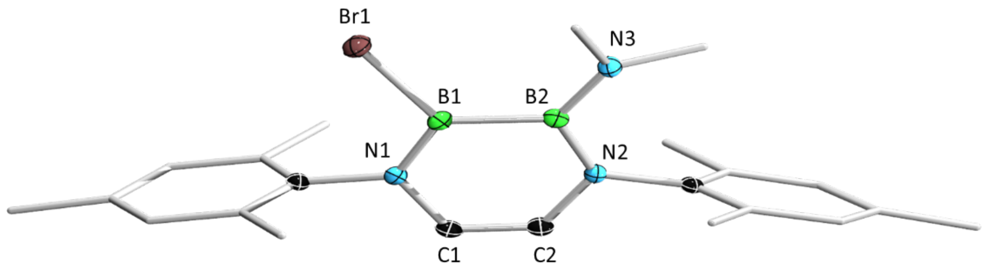

Figure S77. X-ray crystallographic structure of compound **8a**. Ellipsoids represented at 50% probability level. The ellipsoids of the hydrogen and part of the carbon atoms are omitted for clarity. Selected bond lengths (Å), angles and torsion angles (°): B2-B1 1.692(4), B1-N1 1.404(3), N1-C1 1.416(3), C1-C2 1.329(3), C2-N2 1.398(3), N2-B2 1.456(3), B2-N3 1.422(3), B1-Br1 1.969(3), B1-B2-N2 111.3(2), B2-N2-C2 120.9(2), N2-C2-C1 125.7(2), C2-C1-N1 122.8(2), C1-N1-B1 119.2(2), N1-B1-B2 119.3(2), B2-B1-Br1 126.0(2), Br1-B1-N1 114.2(2), B1-B2-N3 125.6(2), N3-B2-N2 122.9(2), B2-N3-C21 121.2(2), B2-N3-C22 128.2(2), B2-B1-N1 C1 1.9(3), B1-N1-C1 C2 -6.0(3), N1-C1-C2 N2 1.4(4), C1-C2-N2 B2 7.7(4), C2-N2-B2 B1 -10.3(3), C1-C2-N2 B2 7.7(4), N2-B2-B1 N1 5.8(3), C2-N2-C12 C13 77.8(3), C1-N1-C3 C8 -82.7(3), B1-B2-N3 C22 -155.5(2).

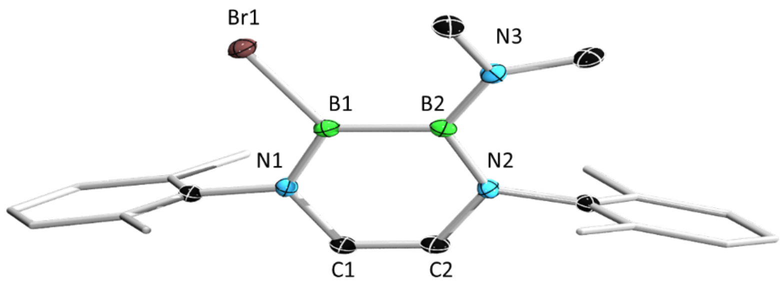

Figure S78. X-ray crystallographic structure of compound **8b**. Ellipsoids represented at 50% probability level and partially omitted for clarity. The ellipsoids of the hydrogen and part of the carbon atoms are omitted for clarity. Selected bond lengths (Å), angles and torsion angles (º): B1-B2 1.694(4), B2-N2 1.456(3), N2-C2 1.401(3), C2-C1 1.328(3), C1-N1 1.417(2), N1-B1 1.403(3), B1-Br1 1.967(3), B2-N3 1.422(3), N1-B1.B2 119.0(2), B1-B2.N2 111.6(2), B2-N2.C2 120.4(2), N2-C2.C1 125.8(2), C2-C1.N1 122.9(2), C1-N1.B1 119.2(2), B1-B2.N3 125.5(2), N3-B2.N2 122.7(2), B2-B1.Br1 125.8(2), Br1-B1-N1 114.6(2), N1-C1-C2-N2 1.3(3), C1-C2-N2-B2 8.6(3), C2-N2-B2-B1 -11.4(3), N2-B2-B1-N1 6.6(3), B2-B1-N1-C1 1.8(3), B1-N1-C1-C2 -6.3(3), C2-N2-C11-C16 -98.5(2), C1-N1-C3-C4 97.0(2), N2-B2-N3-C20 19.3(3), N2-B2-B1-Br1 -164.6(2).

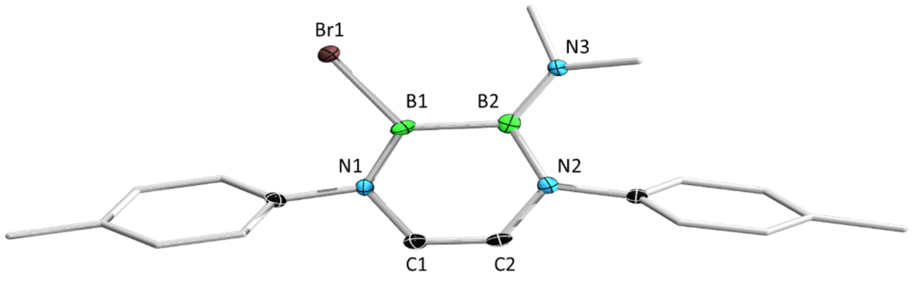

Figure S79. X-ray crystallographic structure of compound **8c**. Ellipsoids represented at 50% probability level. The ellipsoids of the hydrogen and part of the carbon atoms are omitted for clarity. Selected bond lengths (Å), angles and torsion angles (º): B2-B1 1.693(7), B1-N1 1.407(6), N1-C1 1.420(6), C1-C2 1.334(5), C2-N2 1.398(5), N2-B2 1.453(6), B2-N3 1.421(6), B1-Br1 1.965(5), N2-B2-B1 112.3(4), B2-B1-N1 118.6(4), B1-N1-C1 119.3(3), N1-C1-C2 123.6(4), C1-C2-N2 124.9(4), C2-N2-B2 121.4(3), N2-B2-N3 121.0(4), N3-B2-B1 126.5(4), B2-B1-Br1 123.9(3), Br1-B1-N1 117.1(3), N2-C2-C1-N1 -1.7(6), C2-C1-N1-B1 0.4(6), C1-N1-B1-B2 -0.3(6), N1-B1-B2-N2 1.3(6), B1-B2-N2-C2 -2.4(5), B2-N2-C2-C1 2.8(6), C2-N2-C10-C15 -65.9(5), C1-N1-C3-C4 52.0(5), C1-N1-B1-Br1 -172.7(3), B1-B2-N3-C18 147.3(4).

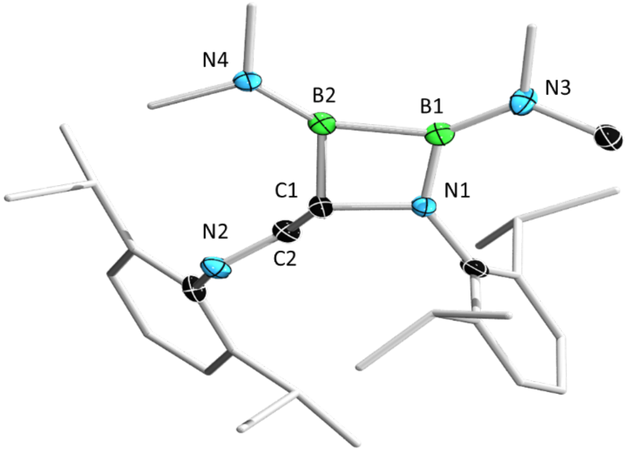

Figure S80. X-ray crystallographic structure of compound **9**. Ellipsoids represented at 50% probability level. The ellipsoids of the hydrogen and part of the carbon atoms are omitted for clarity. Selected bond lengths (Å), angles and torsion angles (°): B2-B1 1.720(3), B1-N3 1.401(3), B2-N4 1.370(3), B2-C1 1.632(3), C1-N1 1.494(2), C1-C2 1.481(3), C2-N2 1.263(3), N4-B2-B1 149.0(2), N4-B2-C1 126.5(2), C1-B2-B1 84.5(1), B2-B1-N1 86.8(1), N1-B1-N3 128.1(2), N3-B1-B2 144.9(2), B2-C1-N1 88.4(1), N1-C1-C2 112.0(1), B2-C1-C2 116.8(2), C1-C2-N2 124.0(2), B2-B1-N1-C1 -1.9(1), B1-N1-C1-B2 2.0(2), N1-C1-B2-B1 -1.7(1), C1-B2-B1-N1 1.8(1), N1-C1-B2-N4 179.0(2), C1-N1-B1-N3 173.8(2), C1-N1-C3-C8 101.6(2), C2-N2-C15-C16 -94.1(2), B1-B2-C1-C2 -115.6(2), B2-C1-C2-N2 -116.5(2), N1-C1-C2-N2 143.8(2).

S3 Computational details

General remarks: All structures were fully optimized using the *GAUSSIAN 09* program.[9] All computations were performed at the ωb97xd level of theory[10] employing 6-31G* basis sets. We ensured that the calculated geometries of **9** and **10** are minima on the potential energy surface by carrying out harmonic frequency calculations (zero negative eigenvalues of the Hessian). The nature of **TS** as real transition state was verified by frequency calculations (one negative eigenvalue of the Hessian).

Cartesian coordinates of DFT optimized structures:

9 E= -1439.828762 a.u.

7	-1.737826000	0.332469000	0.048654000
6	-0.680356000	0.734195000	-0.908248000
5	-2.502082000	1.556069000	0.111132000
5	-1.255783000	2.260800000	-0.835303000
7	-3.765374000	1.787228000	0.690337000
7	-0.700381000	3.403360000	-1.388925000
6	-4.547644000	0.782903000	1.384703000
1	-5.518298000	0.631737000	0.890211000
1	-4.749974000	1.088786000	2.421796000
1	-4.023918000	-0.173505000	1.408931000
6	-4.426262000	3.073500000	0.636044000
1	-4.559410000	3.500019000	1.641532000
1	-5.423610000	2.982653000	0.181262000
1	-3.843802000	3.774574000	0.036386000
6	-1.244621000	4.737378000	-1.201369000
1	-2.065957000	4.712156000	-0.484791000
1	-1.609939000	5.149622000	-2.151277000
1	-0.469779000	5.413293000	-0.816575000
6	0.460080000	3.389218000	-2.268377000
1	1.264970000	4.014950000	-1.860827000
1	0.191839000	3.789639000	-3.254869000
1	0.835538000	2.372513000	-2.396422000
6	0.680530000	0.468423000	-0.388768000
1	-0.785817000	0.271678000	-1.900537000
7	1.652190000	0.086555000	-1.119002000
1	0.807654000	0.615646000	0.689362000
6	2.894505000	-0.152743000	-0.483256000
6	3.629375000	0.871421000	0.144022000
6	3.381916000	-1.477632000	-0.534438000
6	4.855598000	0.537193000	0.729262000
6	4.604885000	-1.761184000	0.068132000
6	5.341725000	-0.761658000	0.699074000
1	5.435769000	1.312578000	1.223617000
1	4.992982000	-2.775317000	0.049268000
6	-1.894695000	-1.029263000	0.438825000
6	-2.453456000	-1.925913000	-0.503317000
6	-1.489752000	-1.492113000	1.709354000
6	-2.555321000	-3.278560000	-0.185778000
6	-1.639880000	-2.858798000	1.985157000
6	-2.146869000	-3.750825000	1.054682000

1	-2.971897000	-3.970306000	-0.913738000
1	-1.339813000	-3.226677000	2.964016000
6	2.533636000	-2.559998000	-1.186359000
1	2.003507000	-2.084796000	-2.020309000
6	3.141884000	2.312680000	0.207625000
1	2.227720000	2.392292000	-0.384216000
6	-2.965916000	-1.452179000	-1.855145000
1	-2.857853000	-0.366197000	-1.897259000
6	-0.872079000	-0.689147000	2.863517000
1	-1.353787000	-1.099392000	3.761395000
6	-4.460627000	-1.753339000	-2.023017000
1	-4.826259000	-1.343140000	-2.971724000
1	-4.660530000	-2.831269000	-2.027538000
1	-5.044832000	-1.304617000	-1.211601000
6	-2.146734000	-2.055609000	-3.003134000
1	-1.078235000	-1.843975000	-2.882336000
1	-2.268816000	-3.144707000	-3.046985000
1	-2.470860000	-1.643542000	-3.965992000
6	-1.106339000	0.821712000	2.934979000
1	-0.783860000	1.176083000	3.921596000
1	-0.541397000	1.377331000	2.183152000
1	-2.162801000	1.078701000	2.817321000
6	0.626261000	-1.012054000	3.009039000
1	0.794245000	-2.088003000	3.123310000
1	1.204916000	-0.682782000	2.140533000
1	1.035804000	-0.510641000	3.894091000
6	2.787982000	2.718194000	1.644432000
1	2.387866000	3.738875000	1.667927000
1	2.034801000	2.047604000	2.074429000
1	3.671084000	2.687802000	2.294103000
6	4.152668000	3.288958000	-0.406210000
1	5.091692000	3.312608000	0.158958000
1	4.387518000	3.014026000	-1.440354000
1	3.743816000	4.306407000	-0.408460000
6	3.349644000	-3.725429000	-1.750252000
1	3.799772000	-4.332307000	-0.955620000
1	2.697774000	-4.386453000	-2.332404000
1	4.152948000	-3.376318000	-2.408713000
6	1.466215000	-3.078901000	-0.209052000
1	0.833188000	-3.831340000	-0.694103000
1	1.936267000	-3.542288000	0.667273000
1	0.813765000	-2.274033000	0.142508000
1	-2.237287000	-4.805401000	1.299485000
1	6.294226000	-0.998780000	1.164825000

10 E= -1439.878162 a.u.

6	-0.665503000	-0.075193000	-1.350711000
1	-1.197191000	-0.115844000	-2.294217000
7	-1.462955000	-0.127992000	-0.198044000
5	-0.863350000	-0.004830000	1.115876000
6	0.665438000	0.074085000	-1.350713000
1	1.197152000	0.114478000	-2.294215000
7	1.462865000	0.127181000	-0.198046000
5	0.863284000	0.004024000	1.115883000
6	2.862517000	-0.016794000	-0.487338000
7	1.670642000	-0.140953000	2.294584000
6	3.427630000	-1.302841000	-0.495270000
7	-1.670718000	0.140333000	2.294537000
6	4.784234000	-1.426047000	-0.806413000
1	5.242980000	-2.411629000	-0.817146000
6	5.552896000	-0.310923000	-1.114889000
6	4.972846000	0.952544000	-1.115328000
1	5.580300000	1.818766000	-1.363555000
6	3.624122000	1.123086000	-0.799455000
6	-2.862529000	0.016726000	-0.487370000
6	-3.426863000	1.303112000	-0.495418000
6	-4.783373000	1.427127000	-0.806640000
1	-5.241507000	2.412992000	-0.817482000
6	-5.552696000	0.312452000	-1.115096000
6	-4.973414000	-0.951367000	-1.115422000
1	-5.581376000	-1.817237000	-1.363635000
6	-3.624815000	-1.122715000	-0.799446000
6	3.035541000	0.312575000	2.481174000
1	3.145444000	0.721913000	3.495837000
1	3.286899000	1.106083000	1.778059000
1	3.777463000	-0.492730000	2.367889000
6	1.225192000	-0.947279000	3.414834000
1	1.248995000	-0.383811000	4.359791000
1	1.879598000	-1.823633000	3.540876000
1	0.209565000	-1.309891000	3.249497000
6	-1.225200000	0.946552000	3.414842000
1	-1.249102000	0.383042000	4.359771000
1	-1.879518000	1.822969000	3.540900000
1	-0.209532000	1.309061000	3.249556000
6	-3.035772000	-0.312795000	2.480970000
1	-3.145923000	-0.722077000	3.495628000
1	-3.287268000	-1.106243000	1.777838000
1	-3.777445000	0.492724000	2.367570000
1	6.605332000	-0.426187000	-1.359238000
1	-6.605044000	0.428344000	-1.359524000
6	3.000888000	2.510671000	-0.799482000
1	2.033149000	2.433122000	-0.294562000
6	2.604054000	-2.547505000	-0.203054000
1	1.598933000	-2.228536000	0.083552000
6	-2.602547000	2.547298000	-0.203231000
1	-1.597600000	2.227744000	0.083342000
6	-3.002434000	-2.510684000	-0.799408000
1	-2.034723000	-2.433745000	-0.294345000
6	-3.176178000	3.351112000	0.968557000

1	-2.515284000	4.191044000	1.212218000
1	-4.164488000	3.763550000	0.732979000
1	-3.275004000	2.726718000	1.862904000
6	-2.463249000	3.417105000	-1.459028000
1	-1.829878000	4.287959000	-1.252827000
1	-2.005848000	2.851779000	-2.278629000
1	-3.438673000	3.783467000	-1.801686000
6	-2.741389000	-2.991607000	-2.233338000
1	-2.261411000	-3.977346000	-2.227039000
1	-3.680135000	-3.075363000	-2.794775000
1	-2.085273000	-2.299736000	-2.772054000
6	-3.844922000	-3.534613000	-0.030217000
1	-4.060143000	-3.193821000	0.988653000
1	-4.800730000	-3.734727000	-0.528134000
1	-3.305654000	-4.486314000	0.038689000
6	3.842629000	3.535088000	-0.030123000
1	4.057920000	3.194367000	0.988756000
1	4.798379000	3.735831000	-0.527898000
1	3.302759000	4.486449000	0.038760000
6	2.739752000	2.991470000	-2.233435000
1	2.259140000	3.976902000	-2.227174000
1	3.678530000	3.075846000	-2.794726000
1	2.084158000	2.299198000	-2.772269000
6	3.178177000	-3.351025000	0.968694000
1	2.517770000	-4.191344000	1.212342000
1	4.166717000	-3.762891000	0.733084000
1	3.276667000	-2.726610000	1.863062000
6	2.465241000	-3.417371000	-1.458871000
1	1.832311000	-4.288555000	-1.252707000
1	2.007582000	-2.852274000	-2.278486000
1	3.440869000	-3.783220000	-1.801493000

TS E= -1439.803805 a.u.

7	1.610290000	-0.161121000	-0.351740000
6	0.780452000	0.270937000	-1.425736000
5	0.991552000	-1.339779000	0.188958000
5	-0.372763000	-1.434131000	-0.816190000
7	1.353591000	-2.101331000	1.317203000
7	-1.091740000	-2.333932000	-1.615482000
6	2.411010000	-1.768429000	2.252984000
1	3.115349000	-2.605251000	2.362788000
1	1.993581000	-1.552575000	3.247253000
1	2.971127000	-0.894708000	1.919532000
6	0.600064000	-3.270601000	1.727231000
1	0.142372000	-3.118123000	2.715930000
1	1.251523000	-4.153872000	1.795176000
1	-0.197336000	-3.484896000	1.013185000
6	-0.521687000	-3.645126000	-1.878399000
1	0.352533000	-3.815881000	-1.245804000
1	-0.206152000	-3.734860000	-2.928069000
1	-1.258432000	-4.434815000	-1.677436000
6	-2.218736000	-2.017746000	-2.471012000

1	-2.965667000	-2.820356000	-2.423757000
1	-1.902455000	-1.911078000	-3.519444000
1	-2.703759000	-1.092911000	-2.156706000
6	-0.571379000	0.474032000	-1.297965000
1	1.240742000	0.420333000	-2.400903000
7	-1.252396000	0.186940000	-0.134785000
1	-1.113171000	0.711201000	-2.215434000
6	-2.643640000	0.350590000	-0.072378000
6	-3.398898000	-0.613338000	0.645444000
6	-3.301417000	1.484171000	-0.614173000
6	-4.787683000	-0.511613000	0.662318000
6	-4.698081000	1.530927000	-0.579497000
6	-5.448978000	0.532661000	0.024435000
1	-5.366109000	-1.263406000	1.194488000
1	-5.206294000	2.386061000	-1.019376000
6	2.733809000	0.639344000	0.010948000
6	3.990517000	0.343024000	-0.552426000
6	2.567266000	1.709086000	0.908639000
6	5.085942000	1.120525000	-0.172685000
6	3.689014000	2.468050000	1.251661000
6	4.939109000	2.174968000	0.722445000
1	6.068292000	0.907364000	-0.583652000
1	3.581751000	3.299632000	1.943719000
6	-2.556246000	2.711874000	-1.131480000
1	-1.484137000	2.559523000	-0.987706000
6	-2.727296000	-1.715116000	1.452830000
1	-1.654178000	-1.654875000	1.270912000
6	4.151926000	-0.817032000	-1.524877000
1	3.190529000	-0.957215000	-2.029094000
6	1.206971000	2.047110000	1.493762000
1	0.507211000	1.261668000	1.197968000
1	-6.533900000	0.587227000	0.033758000
1	5.802684000	2.771781000	1.003303000
6	-2.802028000	2.967536000	-2.623211000
1	-3.860604000	3.178706000	-2.816703000
1	-2.221308000	3.831439000	-2.967341000
1	-2.521640000	2.103419000	-3.236634000
6	-2.917256000	3.955802000	-0.306087000
1	-3.963087000	4.250386000	-0.452007000
1	-2.764605000	3.775741000	0.763471000
1	-2.287532000	4.803101000	-0.602812000
6	-2.935416000	-1.479854000	2.955379000
1	-2.399559000	-2.237307000	3.540497000
1	-2.561524000	-0.493904000	3.253207000
1	-3.996770000	-1.535514000	3.227160000
6	-3.190918000	-3.118499000	1.050843000
1	-4.258844000	-3.266164000	1.252285000
1	-3.016925000	-3.299672000	-0.014690000
1	-2.641082000	-3.878401000	1.619446000
6	5.204764000	-0.558409000	-2.607163000
1	5.039391000	0.401269000	-3.109381000
1	6.222542000	-0.558125000	-2.199993000
1	5.159533000	-1.349720000	-3.363874000
6	4.462317000	-2.119632000	-0.773348000
1	5.383105000	-2.019915000	-0.185599000
1	3.648327000	-2.388739000	-0.093441000

1	4.598667000	-2.946741000	-1.480452000
6	0.678599000	3.366334000	0.915778000
1	-0.333458000	3.566911000	1.285839000
1	1.316892000	4.212398000	1.200746000
1	0.639359000	3.327560000	-0.178838000
6	1.230516000	2.083869000	3.025712000
1	1.605858000	1.139927000	3.437027000
1	1.862132000	2.894701000	3.408421000
1	0.217173000	2.241657000	3.413002000

S4 References

[1] X. Bantreil, S. P. Nolan, *Nature Protocols* **2010**, *6*, 69.

[2] E. Merino, E. Poli, U. Díaz, D. Brunel, *Dalton Transactions* **2012**, *41*, 10913.

[3] I. E. Buys, S. Elgafi, L. D. Field, T. W. Hambley, B. A. Messerle, *Inorg. Chem.* **1994**, *33*, 1539.

[4] Y.-C. Chang, Y.-C. Lee, M.-F. Chang, F.-E. Hong, *J. Organomet. Chem.* **2016**, *808*, 23.

[5] H. Nöth, H. Schick, W. Meister, *J. Organomet. Chem.* **1964**, *1*, 401.

[6] M. Arrowsmith, H. Braunschweig, K. Radacki, T. Thiess, A. Turkin, *Chem. Eur. J.* **2017**, *23*, 2179.

[7] G. Sheldrick, *Acta Crystallogr. Sect. A: Found. Crystallogr.* **2015**, *71*, 3.

[8] G. Sheldrick, *Acta Crystallogr. Sect. A: Found. Crystallogr.* **2008**, *64*, 112.

[9] M. J. Frisch *et al.*, *Gaussian09*, *Revision E.01*, Gaussian Inc., Wallingford CT, USA, **2009**.

[10] J.-D. Chai, M. Head-Gordon, *Phys. Chem. Che. Phys.* **2008**, *10*, 6615.
